# New Members of the *Cinchona* Alkaloids Family: Assembly of the Triazole Heterocycle at the 6′ Position

**DOI:** 10.3390/molecules26113357

**Published:** 2021-06-02

**Authors:** Catalin Vasile Maftei, Martin Heiko Franz, Christian Kleeberg, Ion Neda

**Affiliations:** 1 InnoChemTech GmbH, Hagenring 30, 38106 Braunschweig, Germany; catalinv.maftei@gmail.com (C.V.M.); franz@innochemtech.de (M.H.F.); 2 Institut für Anorganische und Analytische Chemie, Technische Universität ‘Carola-Wilhelmina’ Braunschweig, Hagenring 30, 38106 Braunschweig, Germany; ch.kleeberg@tu-braunschweig.de

**Keywords:** [3 + 2] cycloaddition, *Cinchona* alkaloids, triazoles, quincorine, quincoridine

## Abstract

The substance class of the well-known *Cinchona* alkaloids is widened by 6′-Amino-cinchonine and 6′-Amino-cinchonidine, novel compounds which incorporate a primary amino function in the quinolinic ring system. These key intermediates open the field for a range of fruitful chemistry. Here is described a short and direct pathway for the synthesis of triazole containing derivatives of the above-mentioned substances using the [3 + 2] Huisgen cycloaddition. For this purpose, the amines were first converted into the corresponding azides. Based on this, non-substituted and silyl-protected triazoles were synthesized as examples. Furthermore, didehydrated derivatives of quincorine and quincoridine were used as addition partners, resulting in compounds that carry the quinuclidine ring of the cinchona alkaloids at both ends. Some of these compounds were examined radiographically to investigate the position of the quinuclidine ring to the triazole. The solid-state structures of compounds **10**, **11** and **28** were determined by X-ray diffraction analyses.

## 1. Introduction

**Figure 1:**
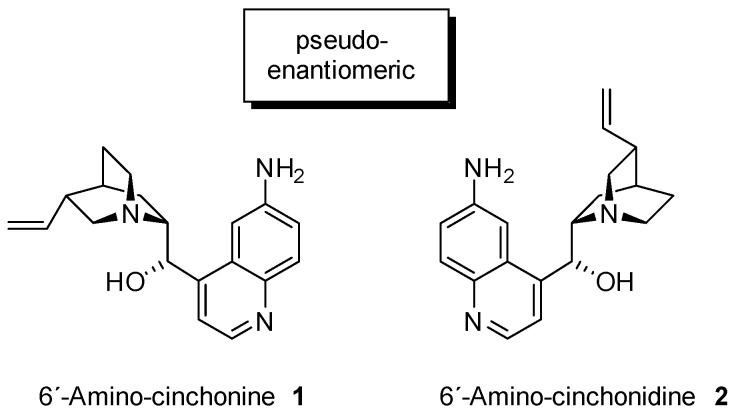
6′-Amino-cinchonine **1** and 6′-Amino-cinchonidine **2**, a pair of pseudo-enantiomeric derivatives of the *Cinchona* alkaloids quinidine and quinine.

To the best of our knowledge, the pseudo-enantiomeric compounds 6′-Amino-cinchonine **1** and 6′-Amino-cinchonidine **2** (Figure 1) are not described in the literature. However, there are a few references to the hydrogenated derivatives, the most recent of which dates from 1960 [1]. It is therefore all the more gratifying that these forgotten derivatives of the *Cinchona* alkaloids are now rationally available.

The benefits of *Cinchona* alkaloids and their derivatives are widely known. Quinine is still used as an anti-malarial in medicine and as a bittering agent in the beverage industry. Cupreins and cupreidines, hydroxy analogues of amines 1 and 2, are currently very popular in catalyst research [2,3,4]. Derivatives of 6′-Amino-cinchonine **1** and 6′-Amino-cinchonidine **2**, as well as the compounds themselves, could have an interesting place in the addressed fields of medicine and catalysis. With our experience [5,6,7,8,9,10,11,12,13,14,15] in the field of this substance class, we began a project to investigate the feasible chemistry more deeply and, thus, generate potentially interesting compounds. Since our group has experience in the general area of cycloadditions [16] and, in particular, the Husigen [3 + 2] cycloaddition [17], we decided to use this technique for the establishment of a triazole unit. The synthesis plan was to convert the amines into the corresponding azides and then couple them to terminal alkynes in a Husigen [3 + 2] cycloaddition. This well-established chemistry, also known as click chemistry, offers a rich pool of diverse reaction possibilities [18,19,20,21,22,23] and thus opens up access to this interesting substitution pattern.

## 2. Results

### 2.1. Chemistry

**Figure 2:**
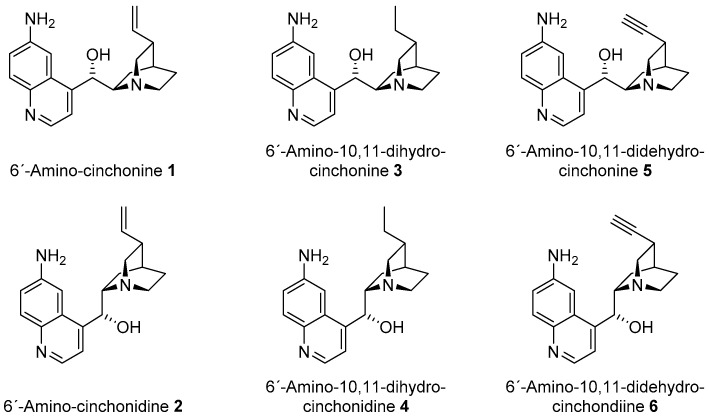
Starting materials: 6′-Amino-cinchonine **1** and 6′-Amino-cinchonidine **2,** plus their hydrogenated derivatives (**3** and **4**) and their dehydrogenated derivatives (**5** and **6**).

In addition to the aforementioned 6′-Amino-cinchonine **1** and 6′-Amino-cinchonidine **2**, these also used the vinyl side chain manipulated derivatives (Figure 2). Moreover, the chemistry was carried out on the hydrogenated compounds **3** and **4** as well as on the dehydrogenated derivatives **5** and **6**. In the case of the latter compounds, the question becomes whether inter- or intramolecular [3 + 2] cycloadditions can be observed.

#### 2.1.1. Introduction of the Azido Functionality

**Scheme 1:**
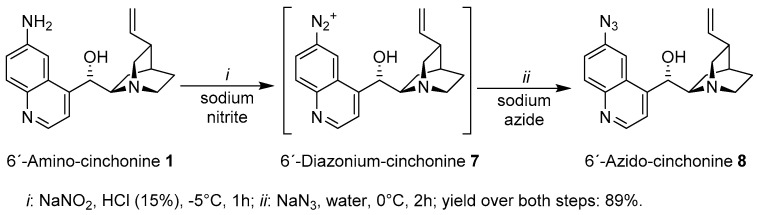
Synthesis of 6′-Azido-cinchonine **8**, starting from 6′-Amino-cinchonine **1** via the diazonium species **7**.

Azide functionality is introduced by a diazotization protocol [24]. Scheme 1 details the conversion of amine **1** to the azido derivative **8**. In the first step, the amino group is converted into the diazo group under acidic conditions and the intermediate diazonium species **7** is formed. Without intermediate work-up, the diazo group is substituted by addition of sodium azide to form the 6′-azido-cinchonine **8** in good yield (89%) and purity. The protocol could also be successfully applied to amines **2** to **6** with similarly good yields (Table 1). Under the conditions chosen, alkynes **5** and **6** also gave no indication of any recognizable spontaneous triazole ring closing. The yields were within the range of the other conversions.

**Scheme 2:**
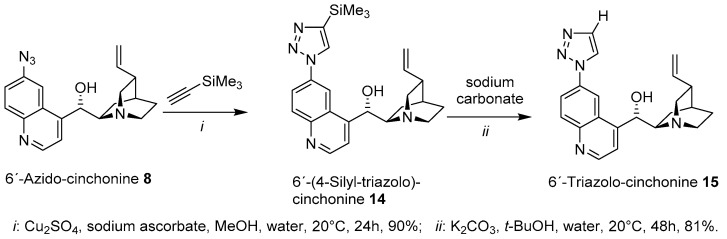
Synthesis of 6′-Triazlo-cinchonine **15**, starting from 6′-Azido-cinchonine **8**. The intermediate was the silylated compound **14**, isolated and characterized.

To establish triazole functionality, azides **8** to **11** were reacted with trimethylsilylacetylene according to the protocol of Zhao et al. [18]. The synthesis protocol originally provided a method in which the cleavage of the silyl group is carried out in situ. In order to see the orientation of the Husigen [3 + 2] cycloaddition, the reaction was carried out in two steps. The silylated species was isolated and characterized. As shown in Scheme 2, only the triazole **14** silylated in position 4 is formed from the azide **8** in a yield of 90%. This was clearly detected by NMR experiments. In a final step, compound **14** was converted to the unsubstituted triazole **15** via E1cb mechanism. Here the yield was 81%.

#### 2.1.2. Assembling of the Triazole Unit

Azides **9**, **10** and **11** were converted into the corresponding silylated triazoles **16**, **18** and **20** and monosubstituted triazoles **17**, **19** and **21** using the same protocol. The yields ranged from good to excellent (89 to 94% for the cyclo-addition and 81–87% for the desilylation).

#### 2.1.3. Assembling of the Triazole Unit Containing a Further Quinuclidine Rest

**Figure 3:**
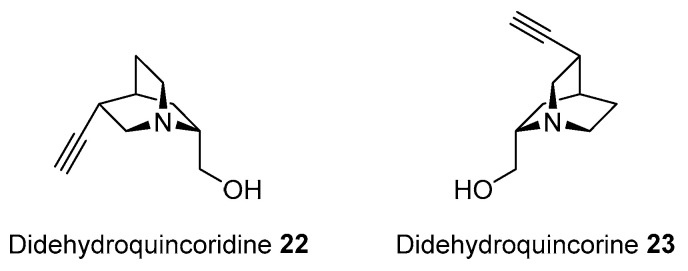
Didehydroquincoridine **22** and didehydrocincorine **23**, *Cinchona* alkaloid based compounds.

After it was shown that the triazole unit can be, in principle, introduced in good yields, more complex molecules were chosen as alkyne sources in a second phase. The alkynes didehydroquincoridine **22** and didehydrocincorine **23** (Figure 3), also compounds derived from the *Cinchona* alkaloids, were chosen.

**Scheme 3:**
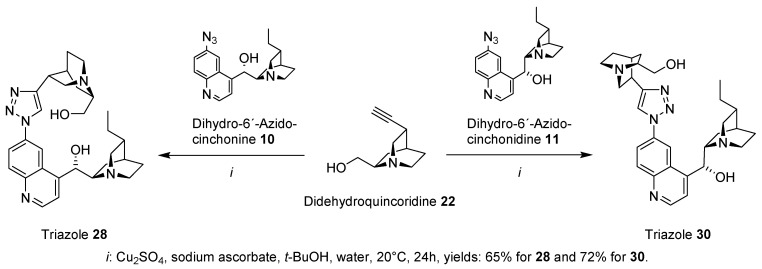
Synthesis of (*S*)-(6-(4-((1*S*,3*R*,4*S*,6*R*)-6-(hydroxymethyl)quinuclidin-3-yl)-1*H*-1,2,3-triazol-1-yl)quinolin-4-yl)((1*S*,2*R*,4*S*,5*R*)-5-ethylquinuclidin-2-yl)methanol (**28**) and one of its diastereomers **30**, starting from azides **10** and **11** using didehydroquincoridine **22**.

Another protocol by Singh et al. was used [19]. The two alkynes **22** and **23** were reacted with the four azides **8**–**11**. Scheme 3 illustrates the two reactions of didehydroquincoridine **22** with the hydrated azides **10** and **11**.

The synthesis of compounds **24** to **31** was again successful in moderate to good yields (62–82%). Two trends can be observed. The yields in the cinchonidine series (azides **9** and **11**, 72–82%) were better than in the cinchonine series (azides **8** and **10**, 62–78%). Although the difference is not strikingly large, it cannot be ignored. Furthermore, in three pairs, the yields of the hydrogenated compounds were higher than the yields of the corresponding vinyl species.

### 2.2. X-ray

**Figure 4:**
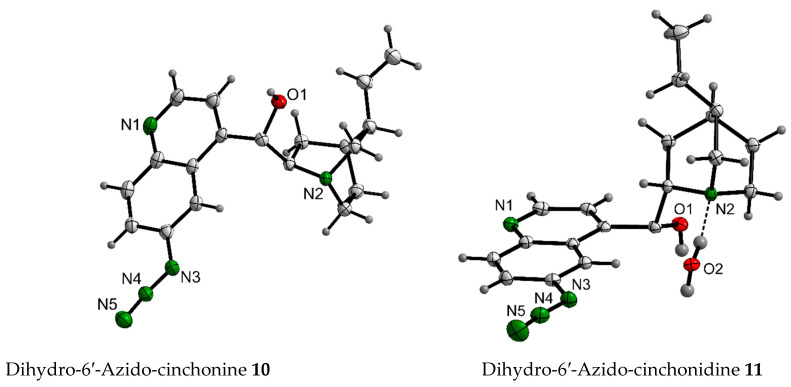
The X-ray analysis for azide **10** and **11**.

**Figure 5:**
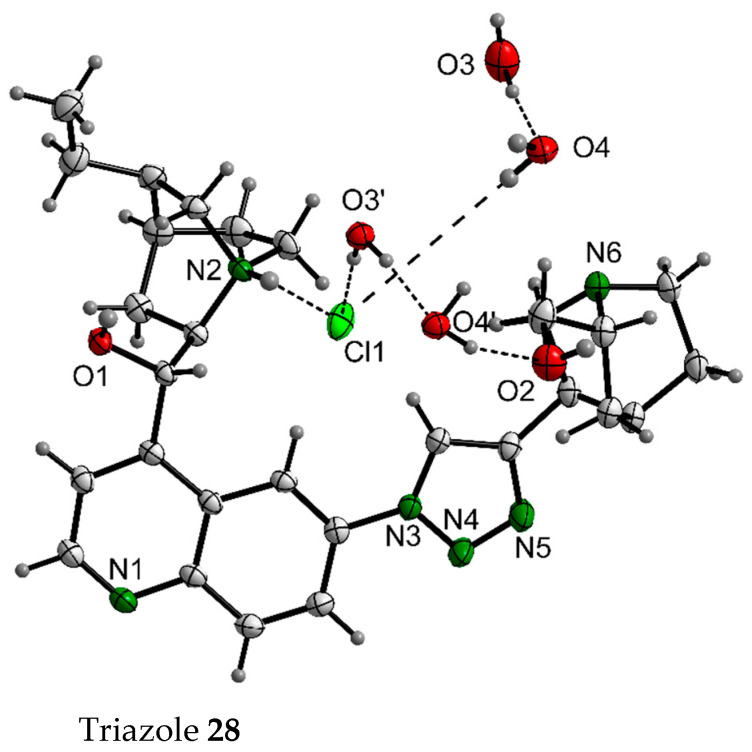
The X-ray analysis for compound **28**.

We investigated three of the synthesized compounds by crystal structure analysis: azides **10** and **11** (Figure 4) and the compound **28** (Figure 5). The compounds **10** and **11** crystalized in their anti-open conformer. Crystallographic data collection parameter of **10**, **11** and **28** can be found in the Supplementary Matericals.

### 2.3. IR Spectroscopy, Melting Point and NMR Experiments

The substances were investigated by infrared spectroscopy. Azides **8** to **13** showed a characteristic band at a wavenumber of *ν* = 2104 to 2108 cm^−1^. The silylated triazoles **14**, **16**, **18** and **20** had a prominent band at a wavenumber of *ν* = 837 to 839 cm^−1^. When the melting point was determined, all synthesized compounds decomposed and gas evolved. In the ^1^H- and ^13^C-NMR experiments, the signals were in the range expected for *Cinchona* alkaloids. Hydroxy protons were not detected at all or only as very broad signals.

### 2.4. Further Experiments

We gave special attention to azide-alkyne **12** in order to perform an intramolecular Husigen [3 + 2] cycloaddition. Simple observations on the model showed that this compound can be built up without significant ring stresses. The only condition is that azide **12** turns into a certain possible conformation, however, this compact molecule eluded our grasp. Protocols [25,26] at elevated temperature led to the formation of amine **5**. In dilute solution [21,27], we observed a non-specific decomposition over time. Using the protocols described in this study, there was mass spectroscopic evidence that oligomeric units were formed.

## 3. Discussion

The aim of this work was to investigate the synthetic potential of 6′-Amino-cinchonine 1, 6′-Amino-cinchonidine **2** and their derivatives and whether they could be successfully converted into the azides **8**–**13**. Using the well-established Husigen [3 + 2] cycloaddition method, the construction of the triazoles **14**, **16**, **18**, **20**, **24**–**31** was successfully performed. Only one addition product was observed in all cases. According to current knowledge, [3 + 2] cycloaddition reaction can proceed via single step-mechanism or stepwise mechanism with a zwitterionic/biradical intermediate. The stepwise mechanism was recently documented for a reaction involving different types of three-atom components, including azides [28]. Since the observed products are stereochemically less hindered, it is likely a main reason for the observed stereochemistry. For considered reactions, the mechanism cannot be proposed a priori since no mechanistic studies were made. The formal addition-products of ethyne to the azide functionality (Triazoles **15**, **17**, **19**, **21**) can be generated by desilylation of the compounds **14**, **16**, **18**, **20**. Overall, all reactions proceeded with good purity and good to very good yields. The aim of future work will be to investigate the potential of these compounds in, for example, catalysis or medicinal chemistry. In terms of structural chemistry, the intramolecular cyclisation of azide alkynes **5** and **6** is a challenge for every synthetic chemist.

## 4. Materials and Methods

### 4.1. Chemistry

#### 4.1.1. General Information

All reagents were purchased from commercial sources (Sigma-Aldrich, TCI, Acros or Buchler) and used without further purification. The solvents used were of analytical grade. The 1H and 13C NMR spectra were recorded at room temperature on a Bruker Avance 300, Bruker Avance 400 or Bruker Avance 600, operating at 300 MHz, 400 MHz or 600 MHz for 1H and 75 MHz, 100 MHz or 150 MHz for 13C. Chemical shifts (δ) were reported as relative to the tetramethylsilane peak set at 0.00 ppm. In the case of multiplets, the signals were reported as intervals. The signals were abbreviated as s, singlet; d, doublet; t, triplet; q, quadruplet; and m, multiplet. Mass spectra were recorded on a Finnigan MAT 8400-MSS and Finnigan MAT 4515. High resolution mass spectra were recorded on a Finnigan MAT 95 XP. The reactions were monitored by TLC and performed on silica gel plates 40 × 80 mm Polygram Sil G/UV254 (Macherey-Nagel). Visualization on TLC was achieved by UV light. Column chromatography was performed with Merck silica gel 60 (70–200 mesh).

The numbering of the *Cinchona* core followed the rules of Paul Rabe, with the side chain numbered based on the IUPAC rules.

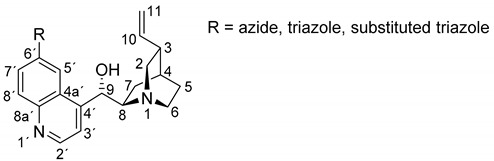



#### 4.1.2. Synthesis of the Azido-Derivatives **8**–**13**

General procedure: a dropwise a solution of NaNO_2_ (1.2 eq) in H_2_O (0.33 g/mL) was added to a mixture of *Cinchona* amine (1.0 eq) and 15% HCl (0.1 g amine/mL) cooled at −5 °C. After the addition was complete, the reaction mixture was stirred at this temperature for 60 min. A solution of NaN_3_ (1.7 eq) in H_2_O (0.2 g/mL) was added dropwise to the reaction mixture at 0 °C. After the addition was finished, the reaction mixture was maintained at 0 °C for 2 h and then stirred at room temperature for 12 h. The product was precipitated by addition of NaOH (20%) until pH 10. Upon filtration, the solid was washed with distilled water and dried. Crystallization from DCM presented the *Cinchona* azides as yellow solids in good yields (70–90%)

*(S)-(6-Azidoquinolin-4-yl)((1S,2R,4S,5R)-5-vinylquinuclidin-2-yl)methanol* (**8**): the title compound was synthesized from 1 (25.0 g, 81 mmol) to afford 8 (25.8 g, yield: 95%) as a yellow-orange solid. ^1^H-NMR (MeOH-d4, 400 MHz) δ 8.79 (d, *J* = 4.6 Hz, H-2′), 8.08 (d, *J* = 9.05 Hz, H-8′), 7.82 (d, *J* = 2.4 Hz, H-5′), 7.74 (d, *J* = 4.6 Hz, H-3′), 7.52 (dd, *J* = 9.05 Hz, *J* = 2.45 Hz, H-7′), 6.17 (ddd, *J* = 17.4 Hz, *J* = 10.3 Hz, *J* = 7.7 Hz, H-10), 5.66–5.52 (m, H-9), 5.17–5.08 (m, H-11, H-11), 3.50–3.43 (m, H-2), 3.35–3.32 (m, H-8), 2.96–2.74 (m, H-2, H-6, H-6), 2.37–2.29 (m, H-3), 2.23–2.16 (m, H-7), 1.93–1.68 (m, H-4), 1.66–1.51 (m, H-5, H-5), and 1.31–1.18 (m, H-7); ^13^C-NMR (MeOH-*d*4, 100 MHz) δ 151.27 (C_q_, C-4′), 150.32 (CH, C-2′), 146.64 (C_q_, C-8a′); 141.73 (CH, C-10); 140.31 (C_q_, C-6′), 132.14 (CH; C-8′), 128.03 (C_q_, C-4a′), 123.87 (CH, C-7′), 120.87 (CH; C-3′), 115.15 (CH_2_, C-11), 112.90 (CH, C-5′), 72.49 (CH; C-9), 61.27 (CH, C-8), 50.82 (CH_2_, C-6), 50.19 (CH_2_, C-2), 41.29 (CH, C-3), 29.65 (CH, C-4), 27.20 (CH_2_, C-5), and 22.25 (CH_2_, C-7); H,H-Cosy, HSQC and HMBC were made. Melting point: the substance decomposed at T = 173 °C; TLC (TBME: MeOH: ammonia 25%/100:10:1): rf-value = 0.60; IR: see spectrum in the supporting information; HR-MS (ESI, MeOH) was calculated for C_19_H_21_N_5_O_1_ + H^+^: 336.18189 Da, found 336.18185 Da.

*(R)-(6-Azidoquinolin-4-yl)((1S,2S,4S,5R)-5-vinylquinuclidin-2-yl)methanol* (**9**): the title compound was synthesized from 2 (25.0 g, 81 mmol) to afford 9 (24.1 g, yield: 89%) as a yellow-orange solid. ^1^H-NMR (MeOH-*d*4, 400 MHz) δ 8.76 (d, *J* = 4.57 Hz, H-2′), 8.05 (d, *J* = 9.05 Hz, H-8′), 7.82 (d, *J* = 2.4 Hz, H-5′), 7.71 (d, *J* = 4.57 Hz, H-3′), 7.52 (dd, *J* = 9.05 Hz, *J* = 2.4 Hz, H-7′), 5.78 (ddd, *J* = 17.7 Hz, *J* = 10.7 Hz, *J* = 7.7 Hz, H-10), 5.53–5.49 (m, H-9), 5.02–4.94 (m, H-11, H-11), 3.61–3.51 (m, H-6), 3.12–2.94 (m, H-2, H-8), 2.72–2.49 (m, H-2, H-6), 2.48–2.18 (m; H-3), 1.93–1.81 (m,H-5, H-7), 1.80–1.78 (m, H-4), and 1.72–1.50 (m, H-5, H-7); ^13^C-NMR (MeOH-*d*4, 100 MHz) δ 151.22 (C_q_, C-4′), 150.27 (CH, C-2′), 146.62 (C_q_, C-8a′); 142.78 (CH, C-10); 140.24 (C_q_, C-6′), 132.15 (CH, C-8′), 127.95 (C_q_, C-4a′), 123.25 (CH, C-7′), 120.93 (CH, C-3′), 114.90 (CH_2_, C-11), 112.99 (CH, C-5′), 72.40 (CH, C-9), 61.53 (CH, C-8), 57.59 (CH_2_, C-2), 43.82 (CH_2_, C-6), 40.95 (CH, C-3), 29.18 (CH, C-4), 28.25 (CH_2_, C-5), and 22.61 (CH_2_, C-7); H,H-Cosy, HSQC and HMBC were made. Melting point: the substance decomposed at T = 144 °C; TLC (TBME: MeOH: ammonia 25%/100:10:1): rf-value = 0.62; IR: see spectrum in the supporting information; HR-MS (ESI, MeOH) was calculated for C_19_H_21_N_5_O_1_ + H^+^: 336.18189 Da, found 336.18183 Da.

*(S)-(6-Azidoquinolin-4-yl)((1S,2R,4S,5R)-5-ethylquinuclidin-2-yl)methanol* (**10**): the title compound was synthesized from 3 (5.0 g, 16.0 mmol) to afford 10 (4.3 g, yield: 79%) as a yellowish solid. ^1^H-NMR (DMSO-*d*6, 400 MHz) δ 8.78 (d, *J* = 4.5 Hz, H-2′), 8.04 (d, *J* = 9.0 Hz, H-8′), 7.86 (d, *J* = 2.5 Hz, H-5′), 7.54 (d, *J* = 4.5 Hz, H-3′), 7.48 (dd, *J* = 9.0 Hz, *J* = 2.5 Hz, H-7′), 5.20–5.16 (m, H-9), 2.96–2.88 (m, H-8), 2.68–2.62 (m, H-6, H-6), 2.52–2.44 (m, H-2, H-2, H-3), 2.00–2.76 (m, H-7), 1.65–1.62 (m, H-4); 1.53–1.30 (m, H-5, H-5, H-7, H-10, H-10), and 0.86 (t, *J* = 7.2 Hz, H-11, H-11, H-11); ^13^C-NMR (DMSO-*d*6, 100 MHz) δ 150.30 (C_q_, C-4′), 149.50 (CH, C-2′), 145.62 (C_q_, C-8a′), 136.85 (C_q_, C-6′), 131.70 (CH, C-8′), 126.94 (C_q_, C-4a′), 121.56 (CH, C-7′), 119.76 (CH, C-3′), 112.15 (CH, C-5′), 70.73 (CH, C-9), 61.14 (CH, C-8), 49.89 (CH_2_, C-6), 49.11 (CH_2_, C-2), 37.08 (CH, C-3), 27.11 (CH_2_, C-5), 26.01 (CH, C-4), 25.03 (CH_2_, C-10), 24.01 (CH_2_, C-7), and 11.90 (CH_3_, C-11); H,H-Cosy, HSQC and HMBC were made. Melting point: the substance decomposed at T = 191 °C; TLC (TBME: MeOH: ammonia 25%/100:10:1): rf-value = 0.50; *IR*: see spectrum in the supporting information; HR-MS (ESI, MeOH) calculated for C_19_H_23_N_5_O_1_ + H^+^: 338.19754 Da, found 338.19756 Da.

*(R)-(6-Azidoquinolin-4-yl)((1S,2S,4S,5R)-5-ethylquinuclidin-2-yl)methanol* (**11**): the title compound was synthesized from 4 (5.0 g, 16 mmol) to afford 8 (4.2 g, yield: 78%) as a yellow solid. ^1^H-NMR (DMSO-*d*6, 400 MHz) δ 8.78 (d, *J* = 4.45 Hz, H-2′), 8.05 (d, *J* = 9.0 Hz, H-8′), 7.91 (d, *J* = 2.4 Hz, H-5′), 7.55 (d, *J* = 4.45 Hz, H-3′), 7.51 (dd, *J* = 9.0 Hz, *J* = 2.4 Hz, H-7′), 5.18–5.13 (m, H-9), 3.21–2.98 (m, H-6, H-8), 2.87–2.77 (m, H-2), 2.45–2.31 (m, H-6), 2.18–2.06 (m, H-2), 1.71–1.50 (m; H-4, H-5, H-7, H-7), 1.40–1.15 (m, H-3, H-5, H-10, H-10), and 0.86 (t, *J* = 7.1 Hz, H-11, H-11, H-11); ^13^C-NMR (DMSO-d6, 100 MHz) δ 149.87 (CH, C-2′), 149.50 (C_q_, C-4′), 145.75 (C_q_, C-8a′); 136.92 (C_q_, C-6′), 131.79 (CH, C-8′), 126.87 (C_q_, C-4a′), 121.45 (CH, C-7′), 119.97 (CH, C-3′), 112.73 (CH, C-5′), 71.44 (CH, C-9),60.59 (CH, C-8), 57.53 (CH_2_, C-2), 41.64 (CH_2_, C-6), 37.14 (CH, C-3), 28.18 (CH_2_, C-5), 27.20 (CH_2_, C-10), 25.14 (CH, C-4), 24.35 (CH_2_, C-7), and 12.04 (CH_3_, C-11); H,H-Cosy, HSQC and HMBC were made. Melting point: the substance decomposed at T = 163 °C; TLC (TBME: MeOH: ammonia 25%/100:10:1): rf-value = 0.54; IR: see spectrum in the supporting information; HR-MS (ESI, MeOH) calculated for C_19_H_23_N_5_O_1_ + H^+^: 338.19754 Da, found 338.19757 Da.

*(S)-(6-Azidoquinolin-4-yl)((1S,2R,4S,5S)-5-ethynylquinuclidin-2-yl)methanol* (**12**): the title compound was synthesized from 5 (1.7 g, 5.5 mmol) to afford 12 (1.8 g, yield: 97%) as a yellow solid. ^1^H-NMR (CDCl_3_, 300MHz) δ 8.73 (d, *J* = 4.5 Hz, H-2′), 8.07 (d, *J* = 9.0 Hz, H-8′), 7.68 (d, *J* = 2.3 Hz, H-5′), 7.53 (d, *J* = 4.5 Hz, H-3′), 7.36 (dd, *J* = 9.0 Hz, *J* = 2.3 Hz, H-7′), 5.58–5.48 (m, H-9), 3.28–3.20 (m, H-2), 3.18–3.09 (m, H-8), 3.03–2.94 (m, H-2), 2.84–2.74 (m, H-6), 2.68–2.55 (m, H-6), 2.51–2.44 (m, H-3), 2.28–2.16 (m, H-7), 2.15 (d, *J* = 2.45 Hz, H-11), 2.07–1.93 (m, H-4), and 1.60–1.41 (m, H-5, H-5, H-7); ^13^C-NMR (CDCl_3_, 75MHz) δ 149.50 (CH, C-2′), 147.93 (C_q_, C-4′), 146.11 (C_q_, C-8a′), 138.16 (C_q_, C-6′), 132.22 (CH, C-8′), 126.81 (C_q_, C-4a′), 121.60 (CH, C-7′), 119.47 (CH, C-3′), 111.73 (CH, C-5′), 87.31 (CH, C-11), 72.01 (CH, C-9), 69.31 (C_q_, C-10), 60.25 (CH, C-8), 50.28 (CH_2_, C-6), 49.41 (CH_2_, C-2), 28.03 (CH, C-3), 27.86 (CH, C-4), 25.11 (CH_2_, C-5), and 23.51 (CH_2_, C-7); H,H-Cosy, HSQC and HMBC were made. Melting point: the substance decomposed at T = 112 °C; TLC (TBME: MeOH: ammonia 25%/100:10:1): rf-value = 0.55; IR: see spectrum in the supporting information; HR-MS (ESI, MeOH) calculated for C_19_H_19_N_5_O_1_ + H^+^: 334.16624 Da, found 334.16647 Da.

*(R)-(6-Azidoquinolin-4-yl)((1S,2S,4S,5S)-5-ethynylquinuclidin-2-yl)methanol* (**13**): the title compound was synthesized from 6 (1.7 g, 5.5 mmol) to afford 13 (1.4 g, yield: 76%) as a yellow solid. ^1^H-NMR (CDCl_3_, 300 MHz) δ 8.74 (d, *J* = 4.5 Hz, H-2′), 8.06 (d, *J* = 9.0 Hz, H-8′), 7.95 (d, *J* = 2.5 Hz, H-5′), 7.51 (d, *J* = 4.5 Hz, H-3′), 7.21 (dd, *J* = 9.0 Hz, *J* = 2.5 Hz, H-7′), 5.61–5.55 (m, H-9), 3.42–3–3.34 (m, H-6), 3.24–3.16 (m, H-8), 2.98–2.88 (m, H-2), 2.75–2.61 (m, H-6), 2.61–2.48 (m, H-2), 2.44–2.37 (m, H-3), 2.16 (d, *J* = 2.4 Hz, H-11), 2.06–1.94 (m,H-5, H-7), 1.90–1.89 (m, H-4), and 1.81–1.59 (m, H-5, H-7); ^13^C-NMR (CDCl_3_, 75MHz) δ 149.65 (CH, C-2′), 148.02 (C_q_, C-4′), 146.31 (C_q_, C-8a′), 138.27 (C_q_, C-6′), 131.97 (CH, C-8′), 126.86 (C_q_, C-4a′), 121.53 (CH, C-7′), 119.67 (CH, C-3′), 111.68 (CH, C-5′), 87.26 (CH, C-11), 71.87 (CH, C-9), 69.21 (C_q_, C-10), 60.62 (CH, C-8), 56.31 (CH_2_, C-2), 49.42 (CH_2_, C-6), 28.17 (CH, C-3), 27.96 (CH, C-4), 24.94 (CH_2_, C-5), and 23.47 (CH_2_, C-7); H,H-Cosy, HSQC and HMBC were made. Melting point: the substance decomposed at T = 96 °C; TLC (TBME: MeOH: ammonia 25%/100:10:1): rf-value = 0.57; IR: see spectrum in the supporting information; *HR-MS* (ESI, MeOH) calculated for C_19_H_19_N_5_O_1_ + H^+^: 334.16624 Da, found 334.16633 Da.

#### 4.1.3. Synthesis of the Silylated Triazole-Derivatives **14**, **16**, **18** and **20**

General procedure: in a round-bottom flask the corresponding *Cinchona* azide (1 eq.) and trimethylsilyl acetylene (1.5 eq.) were suspended in an H_2_O: MeOH (1:1) mixture to deliver a substrate concentration of 0.1 M Cu_2_SO_4_ (0.2 eq., 20 mol%) and sodium ascorbate (0.4 eq., 40 mol%). The reaction mixture was stirred at room temperature until complete conversion of the azide (12–24 h). After that, the reaction was quenched by adding an aqueous solution of ammonium chloride and ammonium hydroxide (pH 10/12). The mixture was extracted with DCM (three times), and the combined organic layers were dried over anhydrous sodium sulphate, filtered and concentrated in vacuo. The residues obtained were purified by flash column chromatography using MTBE: MeOH as a gradient elution to afford the corresponding TMS-triazole derivatives.

*(S)-(6-(4-(Trimethylsilyl)-1H-1,2,3-triazol-1-yl)quinolin-4-yl)((1S,2R,4S,5R)-5-vinylquinuclidin-2-yl)methanol* (**14**): the title compound was synthesized from 8 (1.0 g, 3.0 mmol) to afford 14 (1.15 g, yield: 90%) as a colourless wax. ^1^H-NMR (CDCl_3_, 300 MHz) δ 8.77 (bs, H-Triazole-5), 8.49 (d, *J* = 4.4 Hz, H-2′), 8.10–8.06 (m, H-5′, H-7′), 7.87 (d, *J* = 9.7 Hz, H-8′), 7.35 (d, *J* = 4.4 Hz, H-3′), 6.32 (bs, H-9), 6.06 (ddd, *J* = 17.5 Hz, *J* = 10.1 Hz, *J* = 7.4 Hz, H-10), 5.24–5.18 (m, H-11, H-11), 4.22–3.96 (m, H-2), 3.34–3.08 (m, H-2, H-8), 3.05–2.88 (m, H-6), 2.58–2.42 (m, H-3), 2.37–2.21 (m, H-6), 1.96–1.87 (m, H-4), 1.85–1.71 (m, H-7), 1.69–1.54 (m, H-5), 1.15–1.00 (m, H-5), and 0.93–0.79 (m, H-7) 0.46 (s, 9H, -Si(C*H*_3_)_3_); ^13^C-NMR (CDCl_3_, 75MHz) δ 150.13 (CH, C-2′), 147.41 (C_q_, C-4′), 146.93 (C_q_, C-Triazole-4), 146.62 (C_q_, C-8a′); 137.55 (CH, C-10); 134.68 (C_q_, C-6′), 131.88 (CH; C-8′), 128.35 (CH, C-Triazole-5), 124.52 (C_q_, C-4a′), 121.47 (CH, C-7′), 119.27 (CH; C-3′), 116.78 (CH_2_, C-11), 110.91 (CH, C-5′), 67.67 (CH, C-9), 60.37 (CH, C-8), 49.61 (CH_2_, C-6), 48.71 (CH_2_, C-2), 38.28 (CH, C-3), 27.67 (CH, C-4), 24.31 (CH_2_, C-5), 19.00 (CH_2_, C-7), and -1.06 (CH_3_, -Si*Me*_3_); H,H-Cosy, HSQC and HMBC were made. Melting point: the substance decomposed at T = 147 °C; TLC (TBME: MeOH: ammonia 25%/100:10:1): rf-value = 0.19; IR: see spectrum in the supporting information; HR-MS (ESI, MeOH) calculated for C_24_H_31_N_5_O_1_Si_1_ + H^+^: 434.23706 Da, found 434.23733 Da.

*(R)-(6-(4-(Trimethylsilyl)-1H-1,2,3-triazol-1-yl)quinolin-4-yl)((1S,2S,4S,5R)-5-vinylquinuclidin-2-yl)methanol* (**16**): the title compound was synthesized from 9 (1.0 g, 3.0 mmol) to afford 16 (1.2 g, yield: 92%) as a colourless wax. ^1^H-NMR (CDCl_3_, 300 MHz) δ 8.61 (d, *J* = 4.4 Hz, H-2′), 8.34 (bs, H-Triazol*e*-5), 8.28 (d, *J* = 1.6 Hz, H-5′), 7.97 (d, *J* = 9.1 Hz, H-8′), 7.90 (dd, *J* = 9.1 Hz, *J* = 1.6 Hz, H-7′), 7.52 (d, *J* = 4.4 Hz, H-3′), 5.87–5.75 (m, H-9), 5.69 (ddd, *J* = 17.5 Hz, *J* = 10.3 Hz, *J* = 7.4 Hz, H-10), 5.00–4.85 (m, H-11, H-11), 3.77–3.57 (m, H-6), 3.17–3.03 (m, H-2, H-8), 2.78–2.62 (m, H-2, H-6), 2.41–2.28 (m; H-3), 1.92–1.79 (m, H-4, H-7), 1.62–1.48 (m, H-5, H-5), 0.92–0.81 (m, H-7) 0.41 (s, 9H, -Si(C*H*_3_)_3_); *^13^C-NMR* (CDCl_3_, 75 MHz) δ 150.44 (CH, C-2′), 149.23 (C_q_, C-4′), 147.56 (C_q_, C-Triazole-4), 146.96 (C_q_, C-8a′); 140.20 (CH, C-10); 134.34 (C_q_, C-6′), 131.78 (CH, C-8′), 127.79 (CH, C-Triazole-5), 125.50 (C_q_, C-4a′), 121.54 (CH, C-7′), 119.57 (CH, C-3′), 115.03 (CH_2_, C-11), 112.93 (CH, C-5′), 69.83 (CH, C-9), 60.52 (CH, C-8), 56.12 (CH_2_, C-2), 43.07 (CH_2_, C-6), 39.15 (CH, C-3), 29.63 (CH, C-4), 27.52 (CH_2_, C-5), 21.12 (CH_2_, C-7), and -1.14 (CH_3_, -Si*Me*_3_); H,H-Cosy, HSQC and HMBC were made. Melting point: the substance decomposed at T = 113 °C; TLC (TBME: MeOH: ammonia 25%/100:10:1): rf-value = 0.15; IR: see spectrum in the supporting information; HR-MS (ESI, MeOH) calculated for C_24_H_31_N_5_O_1_Si_1_ + H^+^: 434.23706 Da, found 434.23742 Da.

*(S)-(6-(4-(Trimethylsilyl)-1H-1,2,3-triazol-1-yl)quinolin-4-yl)((1S,2R,4S,5R)-5-ethylquinuclidin-2-yl)methanol* (**18**): the title compound was synthesized from 10 (0.5 g, 1.5 mmol) to afford 18 (0.6 g, yield: 94%) as a colourless honey-type liquid. ^1^H-NMR (CDCl_3_, 300 MHz) δ 8.77 (bs, H-Triazole-5), 8.47 (d, *J* = 4.4 Hz, H-2′), 8.13–8.09 (m, H-5′, H-7′), 7.81 (d, *J* = 9.7 Hz, H-8′), 7.34 (d, *J* = 4.4 Hz, H-3′), 6.38 (bs, H-9), 4.19–3.92 (m, H-2), 3.28–3.04 (m, H-2, H-8), 3.08–2.91 (m, H-6), 2.52–2.37 (m, H-3), 2.31–2.17 (m, H-6), 1.92–1.85 (m, H-4), 1.81–1.70 (m, H-7), 1.61–1.49 (m, H-5), 1.29–1.15 (m, H-5, H-10, H-10), 0.95–0.83 (m, H-7) 0.79 (t, *J* = 7.2 Hz, H-11, H-11, H-11), and 0.42 (s, 9H, -Si(CH_3_)_3_); *^13^C-NMR* (CDCl_3_, 75MHz) δ 150.20 (CH, C-2′), 148.27 (C_q_, C-4′), 147.43 (C_q_, C-*Triazole*-4), 146.70 (C_q_, C-8a′); 134.39 (C_q_, C-6′), 131.74 (CH; C-8′), 128.03 (CH, C-*Triazole*-5), 124.98 (C_q_, C-4a′), 121.33 (CH, C-7′), 119.33 (CH; C-3′), 111.88 (CH, C-5′), 68.77 (CH, C-9), 60.33 (CH, C-8), 50.42 (CH_2_, C-6), 49.67 (CH_2_, C-2), 36.38 (CH, C-3), 26.88 (CH_2_, C-10), 25.58 (CH, C-4), 24.69 (CH_2_, C-5), 19.48 (CH_2_, C-7), 11.75 (CH_3_, C-11), and -1.15 (CH_3_, -Si*Me*_3_); H,H-Cosy, HSQC and HMBC were made. Melting point: the substance decomposed at T = 166 °C; TLC (TBME: MeOH: ammonia 25%/100:10:1): rf-value = 0.11; IR: see spectrum in the supporting information; HR-MS (ESI, MeOH) calculated for C_24_H_33_N_5_O_1_Si_1_ + H^+^: 436.25271 Da, found 436.25296 Da.

*(R)-(6-(4-(Trimethylsilyl)-1H-1,2,3-triazol-1-yl)quinolin-4-yl)((1S,2S,4S,5R)-5-ethylquinuclidin-2-yl)methanol* (**20**): the title compound was synthesized from 11 (0.5 g, 1.5 mmol) to afford 20 (0.57 g, yield: 89%) as a honey-type liquid. ^1^H-NMR (CDCl_3_, 300MHz) δ 8.63 (d, *J* = 4.5 Hz, H-2′), 8.35 (bs, H-Triazole-5), 8.24 (d, *J* = 1.9 Hz, H-5′), 8.00 (d, *J* = 9.1 Hz, H-8′), 7.95 (dd, *J* = 9.1 Hz, *J* = 1.9 Hz, H-7′), 7.51 (d, *J* = 4.5 Hz, H-3′), 5.84–5.73 (m, H-9), 5.69 3.73–3.57 (m, H-6), 3.16–3.02 (m, H-2, H-8), 2.77–2.63 H-6), 2.47–2.33 (m, H-2), 1.93–1.77 (m; H-4, H-7), 1.57–1.40 (m, H-3, H-5, H-7), 1.29–1.15 (m, H-5, H-10, H-10), 0.78 (t, *J* = 7.3 Hz, H-11, H-11, H-11), and 0.43 (s, 9H, -Si(CH_3_)_3_); ^13^C-NMR (CDCl_3_, 75 MHz) δ 150.50 (CH, C-2′), 149.18 (C_q_, C-4′), 147.58 (C_q_, C-Triazole-4), 147.04 (C_q_, C-8a′); 134.45 (C_q_, C-6′), 131.89 (CH, C-8′), 127.83 (CH, C-Triazole-5), 125.49 (C_q_, C-4a′), 121.64 (CH, C-7′), 119.53 (CH, C-3′), 112.84 (CH, C-5′), 69.96 (CH, C-9), 60.36 (CH, C-8), 57.97 (CH_2_, C-2), 43.23 (CH_2_, C-6), 36.95 (CH, C-3), 27.39 (CH_2_, C-5), 27.37 (CH_2_, C-10), 25.18 (CH, C-4), 20,76 (CH_2_, C-7), 11.88 (CH_3_, C-11), and -1.11 (CH_3_, -Si*Me*_3_); H,H-Cosy, HSQC and HMBC were made. Melting point: the substance decomposed at T = 136 °C; TLC (TBME: MeOH: ammonia 25%/100:10:1): rf-value = 0.16; IR: see spectrum in the supporting information; HR-MS (ESI, MeOH) calculated for C_24_H_33_N_5_O_1_Si_1_ + H^+^: 436.25271 Da, found 436.25305 Da.

#### 4.1.4. Synthesis of the Triazole-Derivatives **15**, **17**, **19** and **21** via Desilylation

General procedure: a purified *Cinchona* TMS-triazole (1.00 eq.) was suspended in a mixture *t*Bu-OH/H_2_O (1:1 ratio) and K_2_CO_3_ (2.0 equiv) was added. The reaction mixture was rigorously stirred for 48 h. Upon completion of the reaction, EtOAc was added and the organic layer was separated, washed with water, brine, dried over Na_2_SO_4_, filtered and concentrated in vacuo. The residues obtained were purified by flash column chromatography using MTBE: MeOH as a gradient elution to afford the corresponding triazole products.

*(S)-(6-(1H-1,2,3-Triazol-1-yl)quinolin-4-yl)((1S,2R,4S,5R)-5-vinylquinuclidin-2-yl)methanol* (**15**): the title compound was synthesized from 14 (1.0 g, 2.3 mmol) to afford 15 (672 mg, yield: 81%) as a white solid. ^1^H-NMR (CDCl_3_, 600 MHz) δ 8.68 (d, *J* = 4.5 Hz, H-2′), 8.34 (d, *J* = 2.2 Hz, H-5′), 8.23 (d, *J* = 0.8 Hz, H-Triazole-5), 8.04 (d, *J* = 9.0 Hz, H-8′), 7.93 (dd, *J* = 9.0 Hz, *J* = 2.2 Hz, H-7′), 7.77 (d, *J* = 0.8 Hz, H-Triazole-4), 7.58 (d, *J* = 4.5 Hz, H-3′), 6.04 (ddd, *J* = 16.7 Hz, *J* = 10.3 Hz, *J* = 7.5 Hz, H-10), 5.69–5.66 (m, H-9), 5.08–5.01 (m, H-11, H-11), 3.33–3.25 (m, H-2), 3.06–3.00 (m, H-8), 2.88–2.78 (m, H-2, H-6), 2.72–2.64 (m, H-6), 2.26–2.19 (m, H-3), 2.12–2.03 (m, H-7), 1.80–1.75 (m, H-4), 1.56–1.45 (m, H-5, H-5), and 1.28–1.20 (m, H-7); ^13^C-NMR (CDCl_3_, 150 MHz) δ 150.71 (CH, C-2′), 150.24 (C_q_, C-4′), 147.13 (C_q_, C-8a′); 140.30 (CH, C-10); 134.54 (CH, C-Triazole-4), 134.22 (C_q_, C-6′), 131.86 (CH; C-8′), 125.78 (C_q_, C-4a′), 122.07 (CH, C-Triazole-5), 121.41 (CH, C-7′), 119.60 (CH; C-3′), 114.73 (CH_2_, C-11), 113.70 (CH, C-5′), 71.11 (CH, C-9), 60.25 (CH, C-8), 49.73 (CH_2_, C-6), 49.11 (CH_2_, C-2), 39.78 (CH, C-3), 28.01 (CH, C-4), 26.16 (CH_2_, C-5), and 21.42 (CH_2_, C-7); H,H-Cosy, HSQC and HMBC were made. Melting point: the substance decomposed at T = 117 °C; TLC (TBME: MeOH: ammonia 25%/100:10:1): rf-value = 0.56; IR: see spectrum in the supporting information; HR-MS (ESI, MeOH) calculated for C_21_H_23_N_5_O_1_ + H^+^: 362.19754 Da, found 362.19785 Da.

*(R)-(6-(1H-1,2,3-Triazol-1-yl)quinolin-4-yl)((1S,2S,4S,5R)-5-vinylquinuclidin-2-yl)methanol* (**17**): the title compound was synthesized from 16 (1.0 g, 2.3 mmol) to afford 17 (725 mg, yield: 87%) as a white solid. ^1^H-NMR (CDCl_3_, 600 MHz) δ 8.59 (d, *J* = 4.5 Hz, H-2′), 8.49 (bs, H-Triazole-5), 8.37 (d, *J* = 1.8 Hz, H-5), 8.00 (dd, *J* = 9.1 Hz, *J* = 1.8 Hz, H-7′), 7.95 (d, *J* = 9.1 Hz, H-8′), 7.74 (bs, H-Triazole-4), 7.59 (d, *J* = 4.5 Hz, H-3′), 5.77–5.68 (m, H-9), 5.70 (ddd, *J* = 17.4 Hz, *J* = 10.3 Hz, *J* = 7.5 Hz, H-10), 4.96–4.85 (m, H-11, H-11), 3.67–3.53 (m, H-6), 3.12–2.98 (m, H-2, H-8), 2.73–2.58 (m, H-2, H-6), 2.35–2.25 (m; H-3), 1.92–1.76 (m, H-4, H-5, H-7), and 1.60–1.48 (m, H-5, H-7); ^13^C-NMR (CDCl_3_, 150 MHz) δ 150.19 (CH, C-2′), 150.09 (C_q_, C-4′), 146.62 (C_q_, C-8a′); 140.77 (CH, C-10); 134.14 (CH, C-Triazole-4), 134.12 (C_q_, C-6′), 131.31 (CH; C-8′), 125.31 (C_q_, C-4a′), 122.07 (CH, C-Triazole-5), 121.14 (CH, C-7′), 119.27 (CH; C-3′), 114.41 (CH_2_, C-11), 112.73 (CH, C-5′), 70.03 (CH, C-9), 60.22 (CH, C-8), 56.06 (CH_2_, C-2), 42.70 (CH_2_, C-6), 39.06 (CH, C-3), 27.29 (CH, C-4), 26.73 (CH_2_, C-5), and 20.92 (CH_2_, C-7); H,H-Cosy, HSQC and HMBC were made. Melting point: the substance decomposed at T = 92 °C; TLC (TBME: MeOH: ammonia 25%/100:10:1): rf-value = 0.63; IR: see spectrum in the supporting information; HR-MS (ESI, MeOH) calculated for C_21_H_23_N_5_O_1_ + H^+^: 362.19754 Da, found 362.19796 Da.

*(S)-(6-(1H-1,2,3-Triazol-1-yl)quinolin-4-yl)((1S,2R,4S,5R)-5-ethylquinuclidin-2-yl)methanol* (**19**): the title compound was synthesized from 18 (0.5 g, 1.15 mmol) to afford 19 (359 mg, yield: 86%) as an off-white solid. ^1^H-NMR (CDCl_3_, 300 MHz) δ 8.54 (d, *J* = 4.5 Hz, H-2′), 8.26 (d, *J* = 2.2 Hz, H-5′), 8.35 (d, *J* = 0.8 Hz, H-Triazole-5), 8.98 (d, *J* = 9.0 Hz, H-8′), 7.65 (dd, *J* = 9.0 Hz, *J* = 2.2 Hz, H-7′), 7.98 (d, *J* = 0.8 Hz, H-Triazole-4), 7.94 (d, *J* = 4.5 Hz, H-3′), 5.87–5.77 (m, H-9), 3.31–3.20 (m, H-2), 3.11–3.08 (m, H-8), 2.85–2.73 (m, H-2, H-6), 2.69–2.58 (m, H-6), 2.21–2.15 (m, H-3), 2.32–2.23 (m, H-7), 1.85–1.80 (m, H-4), 1.51–1.40 (m, H-5, H-5, H-10, H-10), 1.22–1.14 (m, H-7), and 0.81 (t, *J* = 7.2 Hz, H-11, H-11, H-11); ^13^C-NMR (CDCl_3_, 75 MHz) δ *^13^C-NMR* (CDCl_3_, 75MHz) δ 150.81 (CH, C-2′), 149.80 (C_q_, C-4′), 147.27 (C_q_, C-8a′), 134.62 (CH, H-*Triazole*-4), 134.30 (C_q_, C-6′), 132.07 (CH; C-8′), 125.75 (C_q_, C-4a′), 122.12 (CH, H-*Triazole*-5), 121.40 (CH, C-7′), 119.60 (CH; C-3′), 113.56 (CH, C-5′), 70.93 (CH, C-9), 60.38 (CH, C-8), 50.74 (CH_2_, C-6), 49.87 (CH_2_, C-2), 37.12 (CH, C-3), 26.76 (CH, C-4), 26.02 (CH_2_, C-10), 25.08 (CH_2_, C-5), 21.09 (CH_2_, C-7), and 11.92 (CH_3_, C-11); H,H-Cosy, HSQC and HMBC were made. Melting point: Substance decompose at T = 143 °C; TLC (TBME: MeOH: ammonia 25%/100:10:1): rf-value = 0.45; IR: see spectrum in the supporting information; HR-MS (ESI, MeOH) calculated for C_21_H_25_N_5_O_1_ + H^+^: 364.21319 Da, found 364.21347 Da.

*(R)-(6-(1H-1,2,3-Triazol-1-yl)quinolin-4-yl)((1S,2S,4S,5R)-5-ethylquinuclidin-2-yl)methanol* (**21**): the title compound was synthesized from 20 (0.5 g, 1.15 mmol) to afford 21 (346 mg, yield: 84%) as an off-white solid. ^1^H-NMR (CDCl_3_, 300 MHz) δ 8.68 (d, *J* = 4.5 Hz, H-2′), 8.37(d, *J* = 2.3 Hz, H-5), 8.29 (d, *J* = 1.0 Hz, H-Triazole-5), 8.05 (d, *J* = 9.1 Hz, H-8′), 7.96 (dd, *J* = 9.1 Hz, *J* = 2.3 Hz, H-7′), 7.80 (d, *J* = 1.0 Hz, H-Triazole-4), 7.57 (d, *J* = 4.57 Hz, H-3′), 5.69–5.59 (m, H-9), 3.47–3.41 (m, H-6), 3.11–2.96 (m, H-2, H-8), 2.65–2.55 (m, H-6), 2.35–2.29 (m, H-2), 1.79–1.71 (m; H-4, H-5, H-7), 1.60–1.41 (m, H-3, H-10, H-10), 1.34–1.18 (m, H-5, H-7), and 0.79 (t, *J* = 7.3 Hz, H-11, H-11, H-11); ^13^C-NMR (CDCl_3_, 75MHz) δ 150.79 (CH, C-2′), 150.07 (C_q_, C-4′), 147.25 (C_q_, C-8a′); 134.60 (CH, H-Triazole-4), 134.36 (C_q_, C-6′), 132.01 (CH, C-8′), 125.80 (C_q_, C-4a′), 122.27 (CH, H-Triazole-5), 121.49 (CH, C-7′), 119.65 (CH, C-3′), 113.51 (CH, C-5′), 70.85 (CH, C-9), 60.39 (CH, C-8), 58.18 (CH_2_, C-2), 43.03 (CH_2_, C-6), 37.20 (CH, C-3), 27.88 (CH_2_, C-5), 27.52 (CH_2_, C-10), 25.27 (CH, C-4), 21.70 (CH_2_, C-7), and 11.96 (CH_3_, C-11); H,H-Cosy, HSQC and HMBC were made. Melting point: the substance decomposed at T = 106 °C; TLC (TBME: MeOH: ammonia 25%/100:10:1): rf-value = 0.58; *IR*: see spectrum in the supporting information; HR-MS (ESI, MeOH) calculated for C_21_H_25_N_5_O_1_ + H^+^: 364.21319 Da, found 364.21358 Da.

#### 4.1.5. Synthesis of the Quinuclidine Containing Triazole-Derivatives **24**–**31**

General procedure: a dropwise a solution of NaNO_2_(1.2 eq) in H_2_O (0.33 g/mL) was added to a mixture of Cinchona amine (1.0 eq) and 15% HCl (0.1g amine/mL) cooled at −5 °C. After the completion of the addition, the reaction mixture was stirred at this temperature for 60 min. A solution of NaN_3_ (1.7 eq) in H_2_O (0.2 g/mL) was added dropwise to the reaction mixture at 0 °C. After the addition was finished, the reaction mixture was maintained at 0 °C for 2 h and then stirred at room temperature for 12 h. The product was precipitated by the addition of NaOH (20%) until pH 10. Upon filtration, the solid was washed with distilled water and dried. Crystallization from DCM presented the *Cinchona* azides as yellow solids in good yields (70–90%).

*(S)-(6-(4-((1S,3R,4S,6R)-6-(Hydroxymethyl)quinuclidin-3-yl)-1H-1,2,3-triazol-1-yl)quinolin-4-yl)((1S,2R,4S,5R)-5-vinylquinuclidin-2-yl)methanol* (**24**): the title compound was synthesized from 8 (1.0 g, 3.0 mmol) to afford 24 (925 mg, yield: 62%) as a reddish solid. ^1^H-NMR (CDCl_3_, 400 MHz) δ 8.87–8.72 (1 H), 8.41–8.28 (2 H), 8.39–7.54 (2 H), 7.69–7.58 (1 H), 6.27–6.56 (1 H), 5.73–5.65 (1 H), 5.21–5.11 (2 H), 3.75–3.29 (4 H), 3.11–2.75 (6 H), 2.89–2.75 (2 H), 2.46–2.33 (1 H), 2.11–2.01 (2 H), 1.78–1.35 (7 H), 1.29–1.15 (1 H), and 0.89–0.76 (1 H); *^13^C-NMR* (CDCl_3_, 100 MHz) δ 151.20 (C_q_), 150.54 (CH), 150.34 (C_q_), 146.96 (C_q_); 140.45 (CH), 134.55 (CH), 131.79 (CH), 125.49 (C_q_), 121.19 (CH), 119.36 (CH), 119.22 (CH), 114.73 (CH_2_), 112.50 (CH), 70.67 (CH), 62.64 (CH_2_), 59.94 (CH), 57.30 (CH), 49.83 (CH_2_), 49.30 (CH_2_), 48.89 (CH_2_), 49.77 (CH_2_), 39.98 (CH), 33.36 (CH), 28.15 (CH), 27.55 (CH_2_), 26.35 (CH), 26.16 (CH_2_), 23.71 (CH_2_), and 20.08 (CH_2_); Melting point: the substance decomposed at T = 126 °C; TLC (TBME: MeOH: ammonia 25%/100:10:1): rf-value = 0.21; IR: see spectrum in the supporting information; HR-MS (ESI, MeOH) calculated for C_29_H_36_N_6_O_2_ + H^+^: 501.29725 Da, found 501.29739 Da.

*(S)-(6-(4-((1S,3R,4S,6S)-6-(Hydroxymethyl)quinuclidin-3-yl)-1H-1,2,3-triazol-1-yl)quinolin-4-yl)((1S,2R,4S,5R)-5-vinylquinuclidin-2-yl)methanol* (**25**): the title compound was synthesized from 8 (1.0 g, 3.0 mmol) to afford 25 (1.0 g, yield: 68%) as a pink solid. ^1^H-NMR (CDCl_3_, 400 MHz) δ 8.71–8.64 (1 H), 8.37–8.25 (2 H), 8.00–7.88 (2 H), 7.62–7.56 (1 H), 6.16–6.04 (1 H), 5.82–5.70 (1 H), 5.13–5.01 (2 H), 3.64–3.19 (4 H), 3.16–2.82 (6 H), 2.80–2.69 (2 H), 2.32–2.21 (1 H), 2.19–2.08 (2 H), 1.82–1.43 (7 H), 1.25–1.13 (1 H), and 0.94–0.83 (1 H); ^13^C-NMR (CDCl_3_, 100 MHz) δ 151.73 (C_q_), 150.36 (CH), 150.24 (C_q_), 146.82 (C_q_); 140.17 (CH), 134.35 (CH), 131.42 (CH), 125.60 (C_q_), 121.08 (CH), 119.35 (CH), 119.12 (CH), 114.71 (CH_2_), 112.86 (CH), 70.3 (CH), 63.33 (CH_2_), 59.59 (CH), 57.28 (CH), 54.75 (CH_2_), 49.61 (CH_2_), 49.20 (CH_2_), 40.44 (CH_2_), 39.75 (CH), 33.02 (CH), 27.96 (CH), 27.20 (CH_2_), 27.06 (CH), 25.97 (CH_2_), 24.89 (CH_2_), and 20.92 (CH_2_). Melting point: the substance decomposed at T = 119 °C; TLC (TBME: MeOH: ammonia 25%/100:10:1): rf-value = 0.20; IR: see spectrum in the supporting information; HR-MS (ESI, MeOH) calculated for C_29_H_36_N_6_O_2_ + H^+^: 501.29725 Da, found 501.29742 Da.

*(R)-(6-(4-((1S,3R,4S,6R)-6-(Hydroxymethyl)quinuclidin-3-yl)-1H-1,2,3-triazol-1-yl)quinolin-4-yl)((1S,2S,4S,5R)-5-vinylquinuclidin-2-yl)methanol* (**26**): the title compound was synthesized from 9 (1.0 g, 3.0 mmol) to afford 26 (1.1 g, yield: 74%) as a light red solid.^1^ H-NMR (CDCl_3_, 300 MHz) δ 8.61–8.54 (1 H), 8.67–8.54 (2 H), 8.13–7.87 (2 H), 7.68–7.46 (1 H), 6.96–6.64 (1 H), 5.34–5.23 (1 H), 5.17–5.03 (2 H), 3.78–3.35 (4 H), 3.26–2.92 (6 H), 2.63–2.46 (2 H), 2.39–2.26 (1 H), 2.12–2.01 (2 H), 1.76–1.32 (7 H), 1.19–1.07 (1 H), and 0.97–0.85 (1 H); ^13^C-NMR (CDCl_3_, 75 MHz) δ 152.08 (C_q_), 150.67 (CH), 148.43 (C_q_), 147.67 (C_q_); 141.21 (CH), 133.96 (CH), 132.54 (CH), 126.31 (C_q_), 122.45 (CH), 120.08 (CH), 119.23 (CH), 115.76 (CH_2_), 111.47 (CH), 69.93 (CH), 62.48 (CH_2_), 61.13 (CH_2_), 60.67 (CH), 57.79 (CH_2_), 56.36 (CH_2_), 54.76 (CH_2_), 42.98 (CH_2_), 40.54 (CH), 34.65 (CH), 27.87 (CH), 26.03 (CH), 25.53 (CH_2_), 24.52 (CH_2_), 21.73 (CH), and 19.26 (CH_2_). Melting point: the substance decomposed at T = 110 °C; TLC (TBME: MeOH: ammonia 25%/100:10:1): rf-value = 0.16; IR: see spectrum in the supporting information; HR-MS (ESI, MeOH) calculated for C_29_H_36_N_6_O_2_ + H^+^: 501.29725 Da, found 501.29747 Da.

*(R)-(6-(4-((1S,3R,4S,6S)-6-(Hydroxymethyl)quinuclidin-3-yl)-1H-1,2,3-triazol-1-yl)quinolin-4-yl)((1S,2S,4S,5R)-5-vinylquinuclidin-2-yl)methanol* (**27**): the title compound was synthesized from 9 (1.0 g, 3.0 mmol) to afford 27 (1.15 mg, yield: 76%) as a pink solid. ^1^H-NMR (CDCl_3_, 300 MHz) δ 8.71–8.64 (1 H), 8.37–8.25 (2 H), 8.00–7.88 (2 H), 7.62–7.56 (1 H), 6.16–6.04 (1 H), 5.82–5.70 (1 H), 5.13–5.01 (2 H), 3.64–3.19 (4 H), 3.16–2.82 (6 H), 2.80–2.69 (2 H), 2.32–2.21 (1 H), 2.19–2.08 (2 H), 1.82–1.43 (7 H), 1.25–1.13 (1 H), and 0.94–0.83 (1 H); ^13^C-NMR (CDCl_3_, 75 MHz) δ 151.99 (C_q_), 150.65 (CH), 147.30 (C_q_), 147.26 (C_q_); 140.48 (CH), 133.16 (CH), 132.17 (CH), 125.47 (C_q_), 121.58 (CH), 119.79 (CH), 119.33 (CH), 115.07 (CH_2_), 112.09 (CH), 69.33 (CH), 63.43 (CH_2_), 60.54 (CH_2_), 60.34 (CH), 57.43 (CH_2_), 55.89 (CH_2_), 54.16 (CH_2_), 43.25 (CH_2_), 40.19 (CH), 33.11 (CH), 27.49 (CH), 26.96 (CH), 26.79 (CH_2_), 24.71 (CH_2_), 21.00 (CH), and 19.88 (CH_2_). Melting point: the substance decomposed at T = 103 °C; TLC (TBME: MeOH: ammonia 25%/100:10:1): rf-value = 0.12; IR: see spectrum in the supporting information; HR-MS (ESI, MeOH) calculated for C_29_H_36_N_6_O_2_ + H^+^: 501.29725 Da, found 501.29753 Da.

*(S)-(6-(4-((1S,3R,4S,6R)-6-(Hydroxymethyl)quinuclidin-3-yl)-1H-1,2,3-triazol-1-yl)quinolin-4-yl)((1S,2R,4S,5R)-5-ethylquinuclidin-2-yl)methanol* (**28**): the title compound was synthesized from 10 (0.5 g, 1.5 mmol) to afford 28 (490 mg, yield: 65%) as an off-white solid. ^1^H-NMR (CDCl_3_, 300 MHz) δ 8.75–8.54 (1 H), 8.28–8.14 (2 H), 8.01–7.82 (2 H), 7.59–7.54 (1 H), 5.73–5.61 (1 H), 3.79–3.27 (4 H), 3.21–2.88 (6 H), 2.81–2.61 (2 H), 2.41–2.32 (1 H), 2.26–2.18 (2 H), 1.89–1.31 (9 H), 1.21–1.12 (1 H), and 0.99–0.81 (4 H); ^13^C-NMR (CDCl_3_, 75 MHz) δ 152.71 (C_q_), 150.98 (CH), 150.37 (C_q_), 147.63 (C_q_), 135.01 (CH), 132.23 (CH), 126.29 (C_q_), 121.78 (CH), 119.73 (CH), 118.61 (CH), 113.93 (CH), 71.35 (CH), 62.67 (CH_2_), 60.59 (CH), 58.79 (CH), 55.23 (CH_2_), 50.69 (CH_2_), 49.13 (CH_2_), 41.65 (CH_2_), 40.72 (CH), 33.19 (CH), 28.17 (CH), 27.64 (CH_2_), 27.13 (CH), 26.27 (CH_2_), 25.93 (CH_2_), 24.63 (CH_2_), 21.47 (CH_2_), and 12.65 (CH_3_). Melting point: the substance decomposed at T = 145 °C; TLC (TBME: MeOH: ammonia 25%/100:10:1): rf-value = 0.19; IR: see spectrum in the supporting information; HR-MS (ESI, MeOH) calculated for C_29_H_38_N_6_O_2_ + H^+^: 503.31290 Da, found 503.31304 Da.

*(S)-(6-(4-((1S,3R,4S,6S)-6-(Hydroxymethyl)quinuclidin-3-yl)-1H-1,2,3-triazol-1-yl)quinolin-4-yl)((1S,2R,4S,5R)-5-ethylquinuclidin-2-yl)methanol* (**29**): the title compound was synthesized from 10 (0.5 g, 1.5 mmol) to afford 29 (580 mg, yield: 78%) as an off-white solid; ^1^H-NMR (CDCl_3_, 400 MHz) δ 9.03–8.73 (1 H), 8.41–8.29 (2 H), 8.13–7.91 (2 H), 7.68–7.59 (1 H), 6.23–5.81 (1 H), 3.69–3.23 (4 H), 3.17–2.81 (6 H), 2.75–2.34 (2 H), 2.32–2.23 (1 H), 2.16–2.01 (2 H), 1.87–1.34 (9 H), 1.27–1.14 (1 H), and 0.91–0.87 (4 H); ^13^C-NMR (CDCl_3_, 100 MHz) δ 151.54 (C_q_), 151.12 (CH), 150.27 (C_q_), 146.12(C_q_), 135.69 (CH), 130.19 (CH), 125.94 (C_q_), 121.98 (CH), 120.15 (CH), 119.72 (CH), 113.26 (CH), 71.3 (CH), 63.99 (CH_2_), 60.57 (CH), 57.52 (CH), 55.75 (CH_2_), 49.69 (CH_2_), 49.13 (CH_2_), 41.64 (CH_2_), 40.73 (CH), 33.92 (CH), 27.56 (CH), 27.21 (CH_2_), 27.16 (CH), 25.47 (CH_2_), 25.22 (CH_2_), 24.83 (CH_2_), 21.62 (CH_2_), and 12.35 (CH_3_). Melting point: the substance decomposed at T = 138 °C; TLC (TBME: MeOH: ammonia 25%/100:10:1): rf-value = 0.21; IR: see spectrum in the supporting information; HR-MS (ESI, MeOH) calculated for C_29_H_38_N_6_O_2_ + H^+^: 503.31290 Da, found 503.31298 Da.

*(R)-(6-(4-((1S,3R,4S,6R)-6-(Hydroxymethyl)quinuclidin-3-yl)-1H-1,2,3-triazol-1-yl)quinolin-4-yl)((1S,2S,4S,5R)-5-ethylquinuclidin-2-yl)methanol* (**30**): the title compound was synthesized from 11 (0.5 g, 1.5 mmol) to afford 30 (535 mg, yield: 72%) as an off-white solid. ^1^H-NMR (CDCl_3_, 300 MHz) δ 8.62–8.49 (1 H), 8.38–8.27 (2 H), 7.99–7.81 (2 H), 7.71–7.60 (1 H), 5.89–5.65 (1 H), 5.17–4.89 (2 H), 3.73–3.29 (4 H), 3.21–2.94 (6 H), 2.81–2.73 (2 H), 2.54–2.36 (1 H), 2.28–2.17 (2 H), 1.93–1.49 (9 H), 1.29–1.14 (1 H), and 0.97–0.73 (4 H); ^13^C-NMR (CDCl_3_, 75 MHz) δ 152.21 (C_q_), 151.25 (CH), 147.91 (C_q_), 146.66 (C_q_); 133.73 (CH), 131.17 (CH), 125.97 (C_q_), 122.68 (CH), 120.19 (CH), 119.13 (CH), 112.34 (CH), 69.78 (CH), 64.41 (CH_2_), 61.64 (CH_2_), 60.52 (CH), 58.67 (CH_2_), 56.37 (CH_2_), 54.73 (CH_2_), 44.87 (CH_2_), 40.36 (CH), 33.57 (CH), 28.19 (CH), 27.91 (CH), 26.71 (CH_2_), 25.52 (CH_2_), 24.11 (CH_2_), 21.68 (CH), 20.18 (CH_2_), and 12.37 (CH_3_). Melting point: the substance decomposed at T = 132 °C; TLC (TBME: MeOH: ammonia 25%/100:10:1): rf-value = 0.14; IR: see spectrum in the supporting information; HR-MS (ESI, MeOH) calculated for C_29_H_38_N_6_O_2_ + H^+^: 503.31290 Da, found 503.31312 Da.

*(R)-(6-(4-((1S,3R,4S,6S)-6-(Hydroxymethyl)quinuclidin-3-yl)-1H-1,2,3-triazol-1-yl)quinolin-4-yl)((1S,2S,4S,5R)-5-ethylquinuclidin-2-yl)methanol* (**31**): the title compound was synthesized from 11 (0.5 g, 1.5 mmol) to afford 31 (610 mg, yield: 82%) as an off-white solid. ^1^H-NMR (CDCl_3_, 300 MHz) δ 8.89–8.71 (1 H), 8.39–8.31 (2 H), 8.23–8.12 (2 H), 7.73–7.66 (1 H), 5.79–5.65 (1 H), 5.56–5.23 (2 H), 3.81–3.39 (4 H), 3.25–2.93 (6 H), 2.85–2.71 (2 H), 2.54–2.35 (1 H), 2.23–2.13 (2 H), 1.97–1.51 (9 H), 1.33–1.21 (1 H), and 0.89–0.68 (4 H); ^13^C-NMR (CDCl_3_, 75 MHz) δ 152.00 (C_q_), 150.69 (CH), 150.35 (C_q_), 147.34 (C_q_); 135.10 (CH), 132.16 (CH), 125.65 (C_q_), 121.59 (CH), 119.93 (CH), 119.42 (CH), 112.46 (CH), 69.27 (CH), 63.47 (CH_2_), 60.59 (CH_2_), 60.33 (CH), 57.67 (CH_2_), 57.43 (CH_2_), 54.13 (CH_2_), 40.22 (CH_2_), 36.83 (CH), 33.10 (CH), 27.40 (CH), 27.00 (CH), 26.89 (CH_2_), 25.16 (CH_2_), 24.79 (CH_2_), 20.99 (CH), 20.10 (CH_2_), and 11.83 (CH_3_). Melting point: the substance decomposed at T = 121 °C; TLC (TBME: MeOH: ammonia 25%/100:10:1): rf-value = 0.14; IR: see spectrum in the supporting information; HR-MS (ESI, MeOH) calculated for C_29_H_38_N_6_O_2_ + H^+^: 503.31290 Da, found 503.31306 Da.

#### 4.1.6. X-ray Structure Determination

The single crystals were mounted on a Hampton loop using perfluoroether oil and placed in the cold nitrogen gas stream on the diffractometer [29]. The data were collected on a Rigaku Oxford Diffraction Synergy-S using mirror-focused CuK_α_ radiation. The reflections were indexed, integrated, and appropriate absorption corrections were applied as implemented in the CrysAlisPro software package [30]. The structures were solved employing the program SHELXT and refined an isotropically for all non-hydrogen atoms by full-matrix least squares on all F2 using SHELXL software [31,32]. Carbon bound hydrogen atoms were refined employing a riding model; methyl groups were treated as rigid bodies and were allowed to rotate about the E–CH3 bond. Nitrogen and oxygen bound hydrogen atoms were located in the difference Fourier map and were refined freely for **10** and **11**, whilst for **28** geometrical restraints and constraints for the displacement parameters were employed. For **28**, the structure contains a partly occupied HCl and two partly occupied units of water. The absolute structure parameter suggests numerically a very small contribution of an inversion twined component. As the only heavy atom (Cl1) is only partly occupied, a twin refinement was considered not reliable and was not conducted. During refinement and analysis of the crystallographic data, the programs OLEX2 and Diamond were used [33,34]. Unless noted otherwise, the shown ellipsoids represent the 50% probability level and hydrogen atoms are displayed with an arbitrary radius.

## Figures and Tables

**Table 1:** Yields of the transformation of the amins **2** to **6** to the corresponding azides **9** to **13**.

Amino Compound (Starting Material)	Azido Compound (Product)	Yield
6′-Amino-cinchonidine **2**	6′-Azido-cinchonidine **9**	96%
Dihydro-6′-Amino-cinchonine **3**	Dihydro-6′-Azido-cinchonine **10**	78%
Dihydro-6′-Amino-cinchonidine **4**	Dihydro-6′-Azido-cinchonidine **11**	79%
Didehydro-6′-Amino-cinchonine **5**	Didehydro-6′-Azido-cinchonine **12**	97%
Didehydro-6′-Amino-cinchonidine **6**	Didehydro-6′-Azido-cinchonidine **13**	94%

## Data Availability

The data presented in this study are available on request from the corresponding author. Crystallographic data have been deposited with the Cambridge Crystallographic Data Centre as supplementary publications no. CCDC-2080994(**10**), -2080992(**11**), -2080993(**28**). Copies of the data can be obtained free of charge from www.ccdc.cam.ac.uk/data_request/cif (accessed on 30 April 2021).

## Supplementary Materials

The following are available online. Hardcopy from 1H-NMR and 13C-Nmr experiments; IR spectrograms and Table S1: Crystallographic data collection parameter of **10**, **11** and **28**.

## Sample Availability

Samples of the compounds **1** to **6** and **8** to **13** are available from the authors.
