# Supplementary material for: New Members of the Cinchona Alkaloids Family: Assembly of the Triazole Heterocycle at the 6′ Position
Source: Molecules. 2021 Jun 2;26(11):3357. doi: 10.3390/molecules26113357 (PMC8199664; doi:10.3390/molecules26113357)
Supplement: Supplementary file 1 [file molecules-26-03357-s001.zip › molecules-1224923-supplementary.pdf]

Supporting Information

New members of the *Cinchona* Alkaloids family: Assembly of the triazole heterocycle at the 6' position.

Catalin Vasile Maftei [1], Martin Heiko Franz [1], Christian Kleeberg [2] and Ion Neda [2,*]

[1] InnoChemTech GmbH, Hagenring 30, 38106 Braunschweig, Germany; catalinv.maftei@gmail.com (C.V.M.); franz@innochemtech.de (M.H.F.)
[2] Institut für Anorganische und Analytische Chemie, Technische Universität `Carola-Wilhelmina` Braunschweig, Hagenring 30, 38106 Braunschweig, Germany; ch.kleeberg@tu-braunschweig.de (C.K.), i.neda@tu-braunschweig.de (I.N.)
* Correspondence: i.neda@tu-braunschweig.de

Content

NMR experiments 2-21

IR spectra 22-32

x-Ray information 33

^{1}H- and ^{13}C-NMR experiment

1H- and 13C-NMR experiment for (S)-(6-azidoquinolin-4-yl)((1S,2R,4S,5R)-5-vinylquinuclidin-2-yl)methanol (**8**):

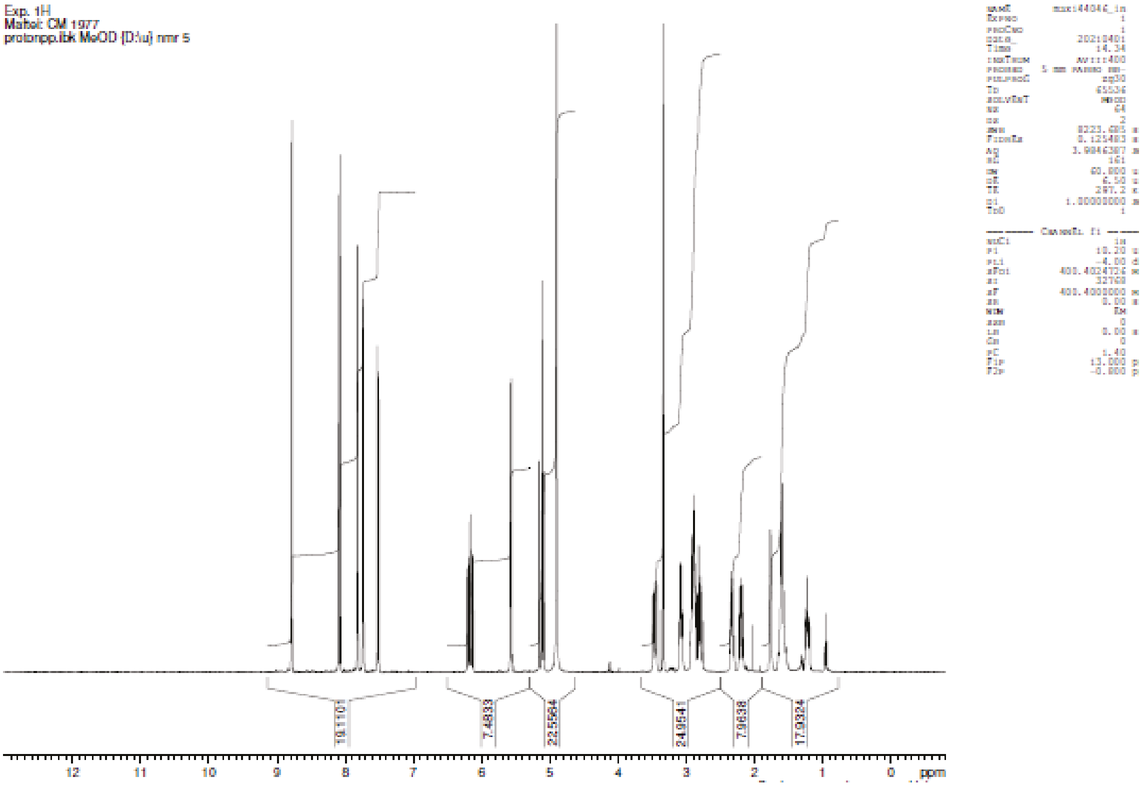

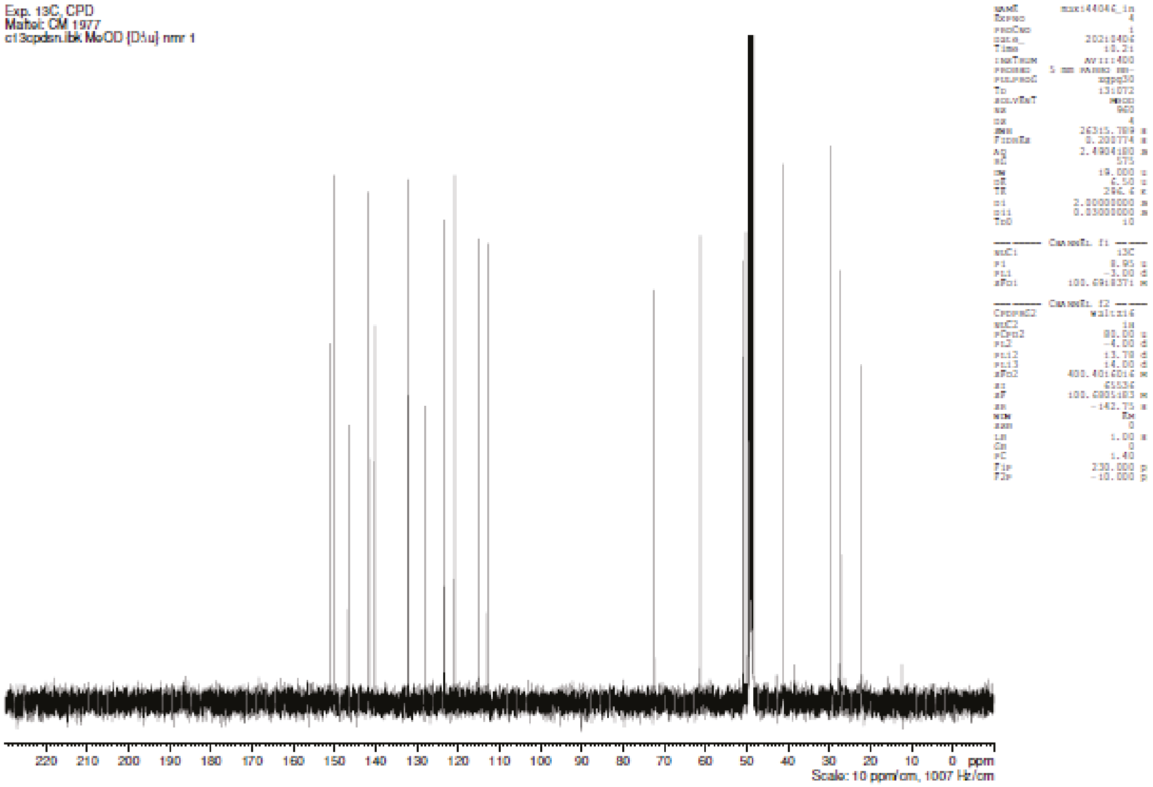

1H- and 13C-NMR experiment for (R)-(6-azidoquinolin-4-yl)((1S,2S,4S,5R)-5-vinylquinuclidin-2-yl)methanol (**9**):

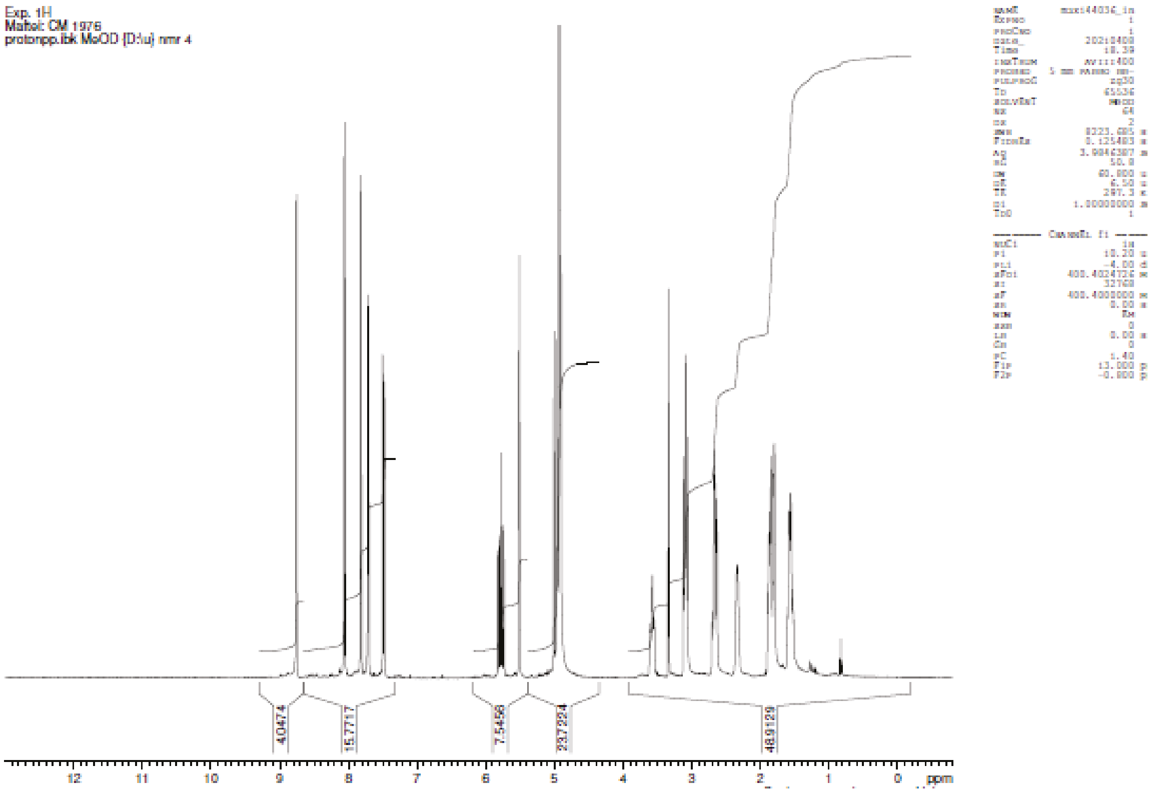

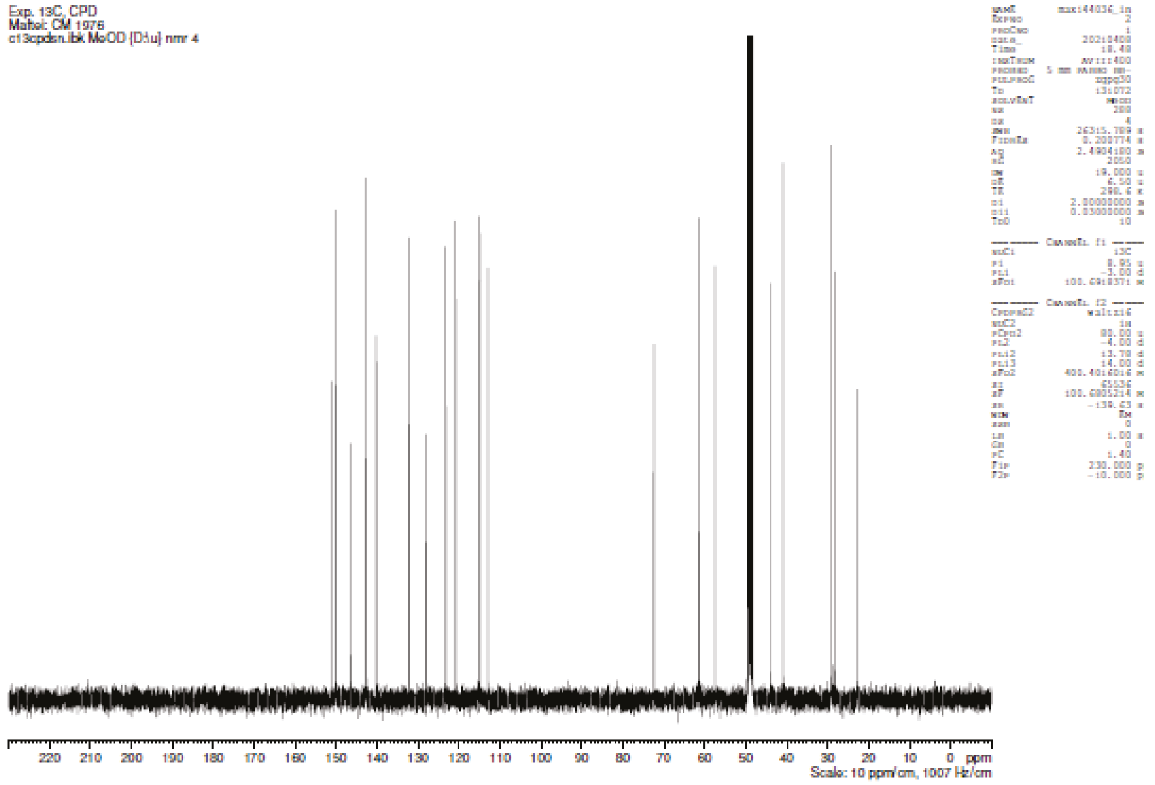

1H- and 13C-NMR experiment for (S)-(6-azidoquinolin-4-yl)((1S,2R,4S,5R)-5-ethylquinuclidin-2-yl)methanol (**10**):

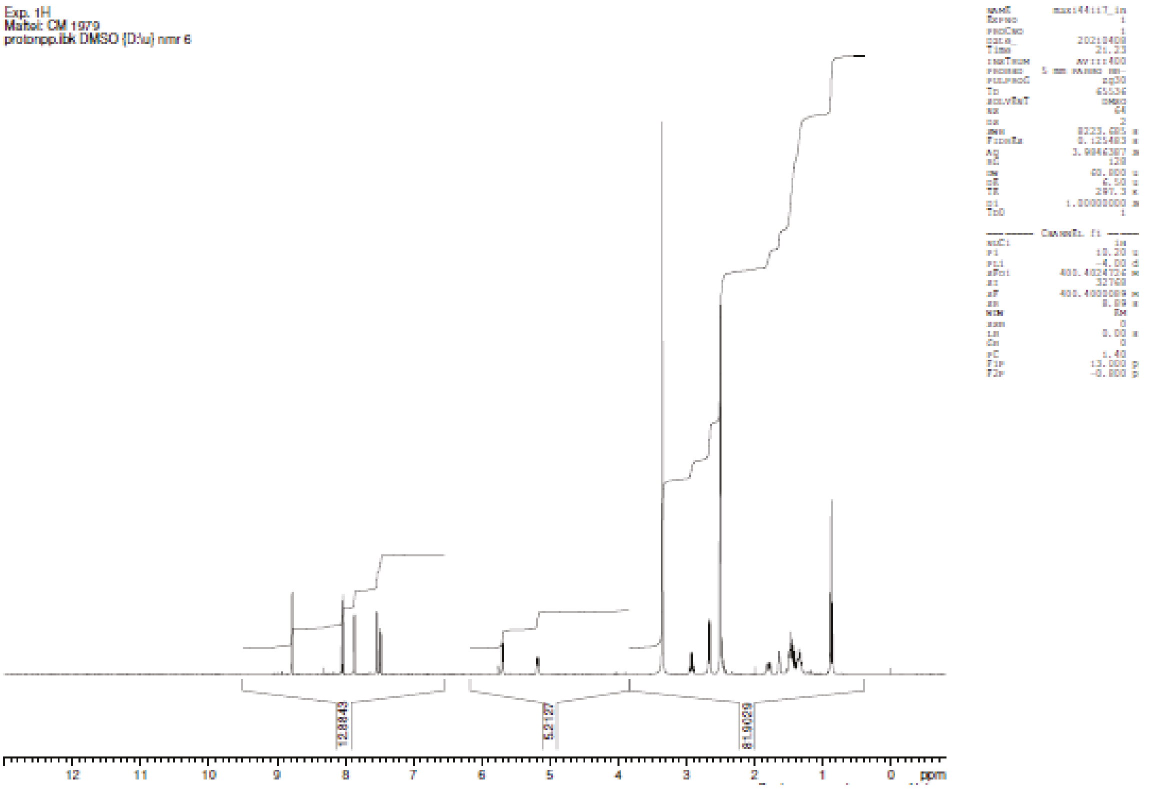

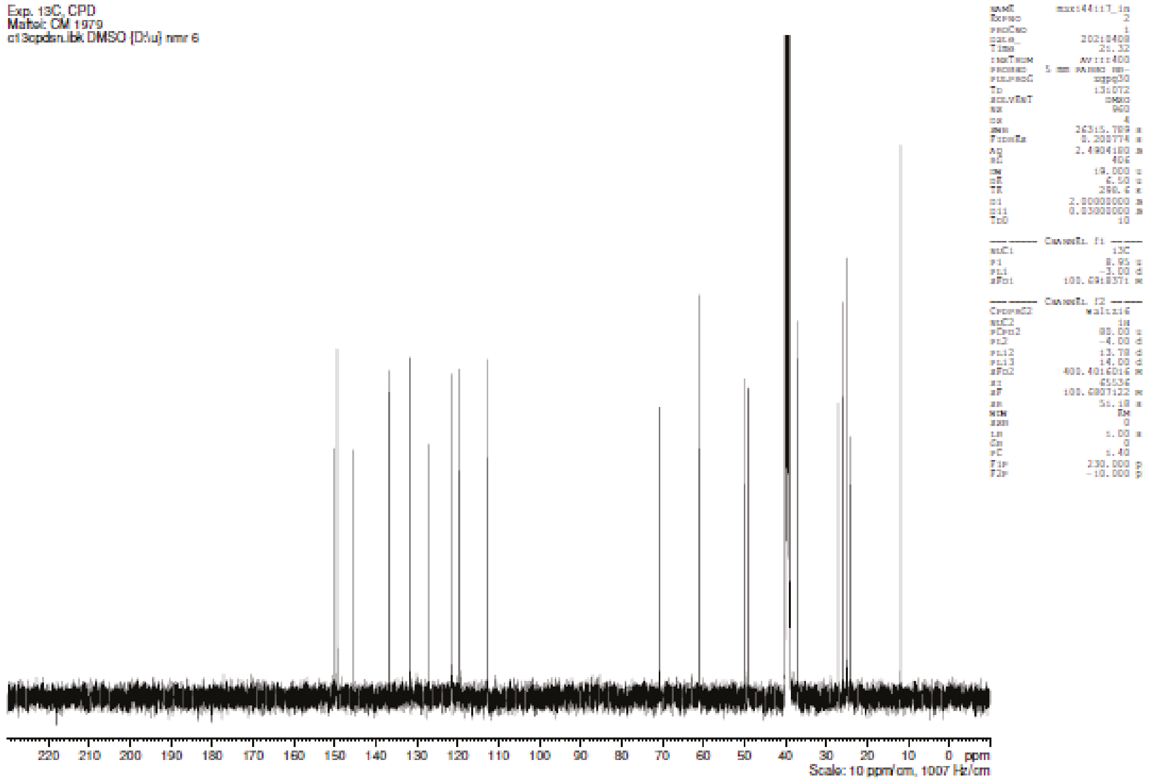

1H- and 13C-NMR experiment for (R)-(6-azidoquinolin-4-yl)((1S,2S,4S,5R)-5-ethylquinuclidin-2-yl)methanol (**11**):

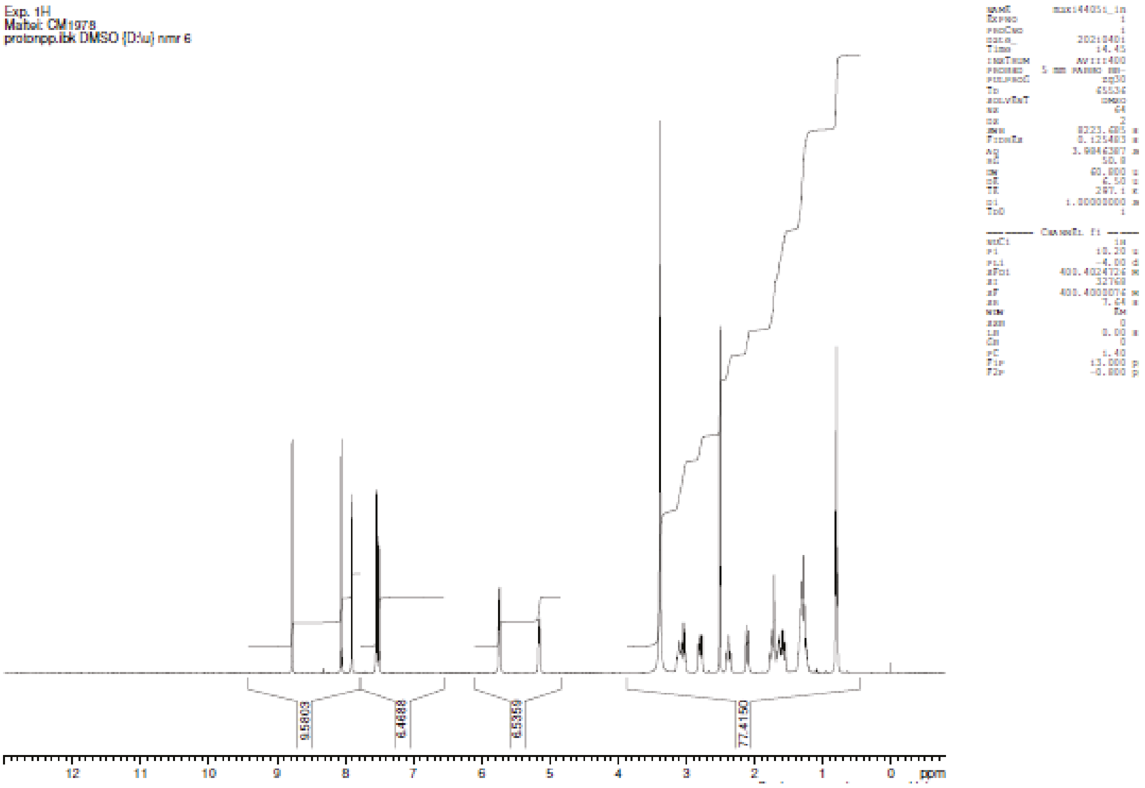

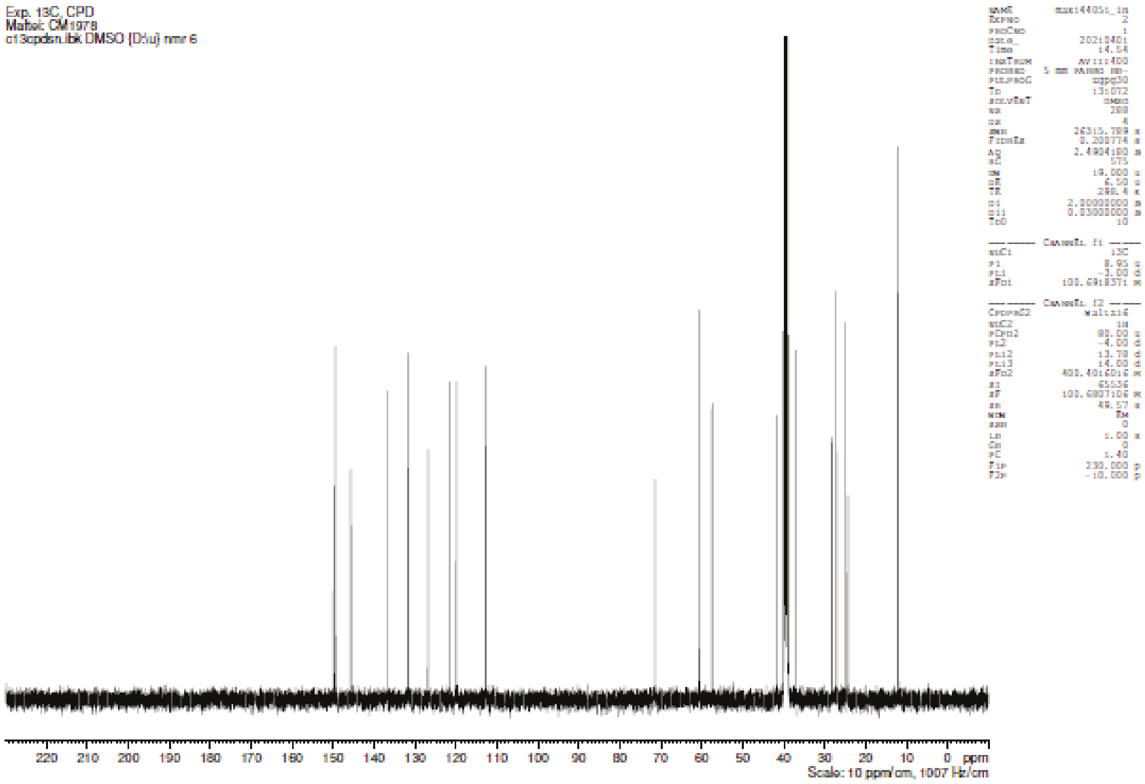

1H- and 13C-NMR experiment for (S)-(6-azidoquinolin-4-yl)((1S,2R,4S,5S)-5-ethynylquinuclidin-2-yl)methanol (**12**):

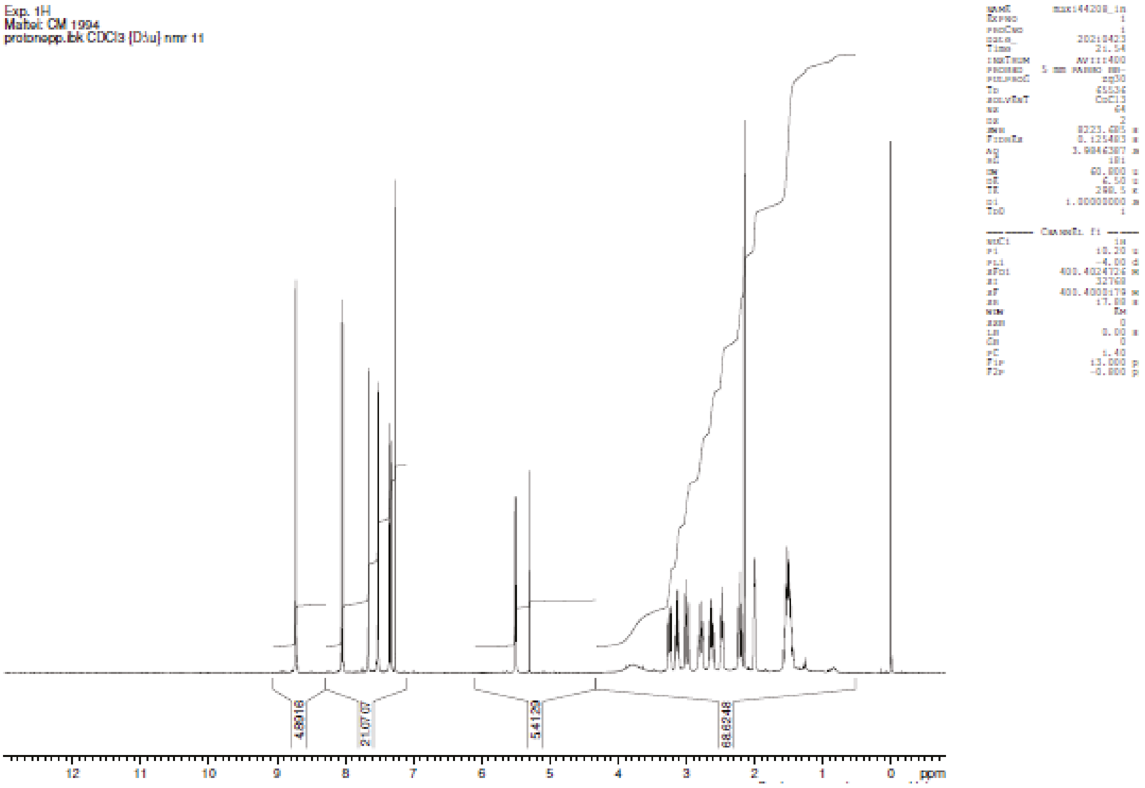

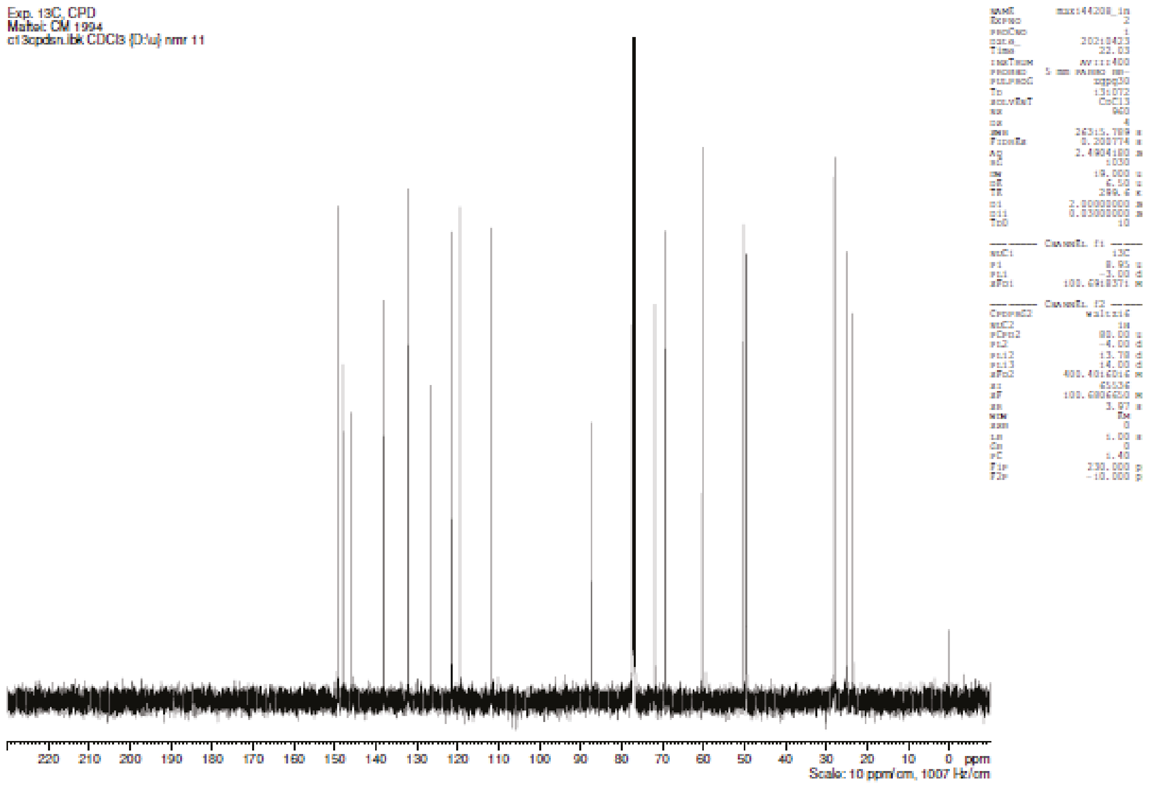

1H- and 13C-NMR experiment for (S)-(6-(4-(trimethylsilyl)-1H-1,2,3-triazol-1-yl)quinolin-4-yl)((1S,2R,4S,5R)-5-vinylquinuclidin-2-yl)methanol (**14**):

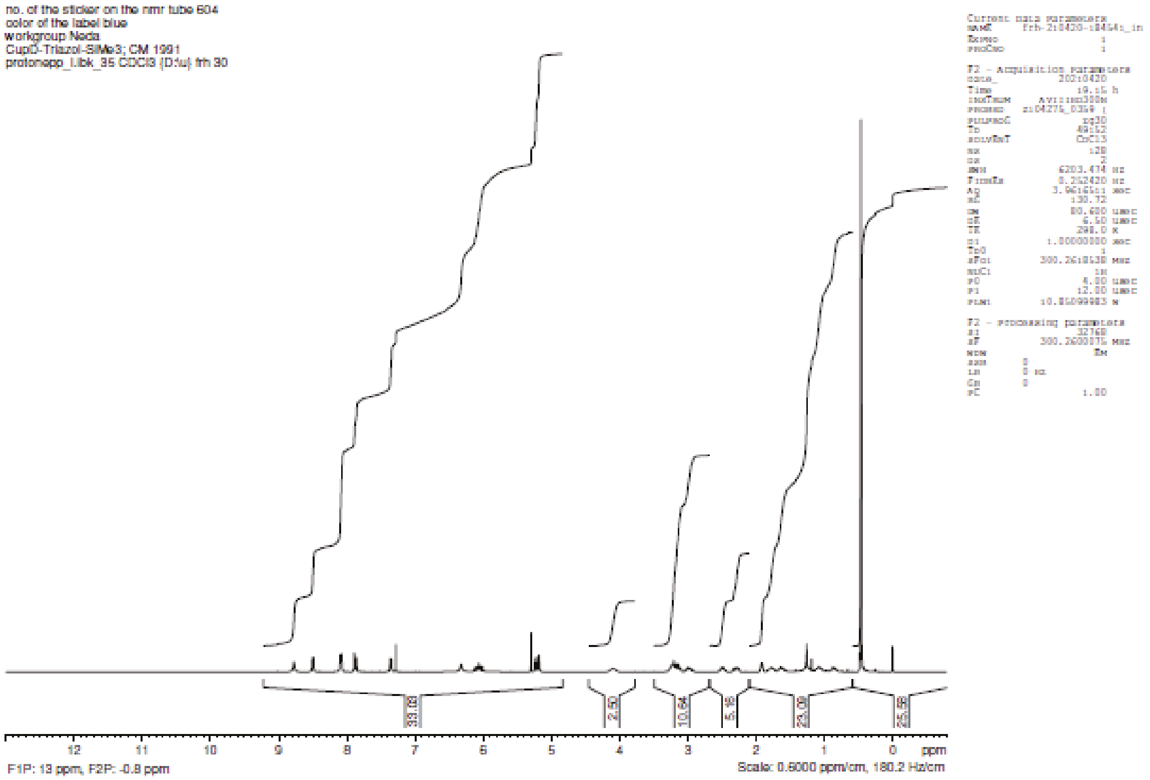

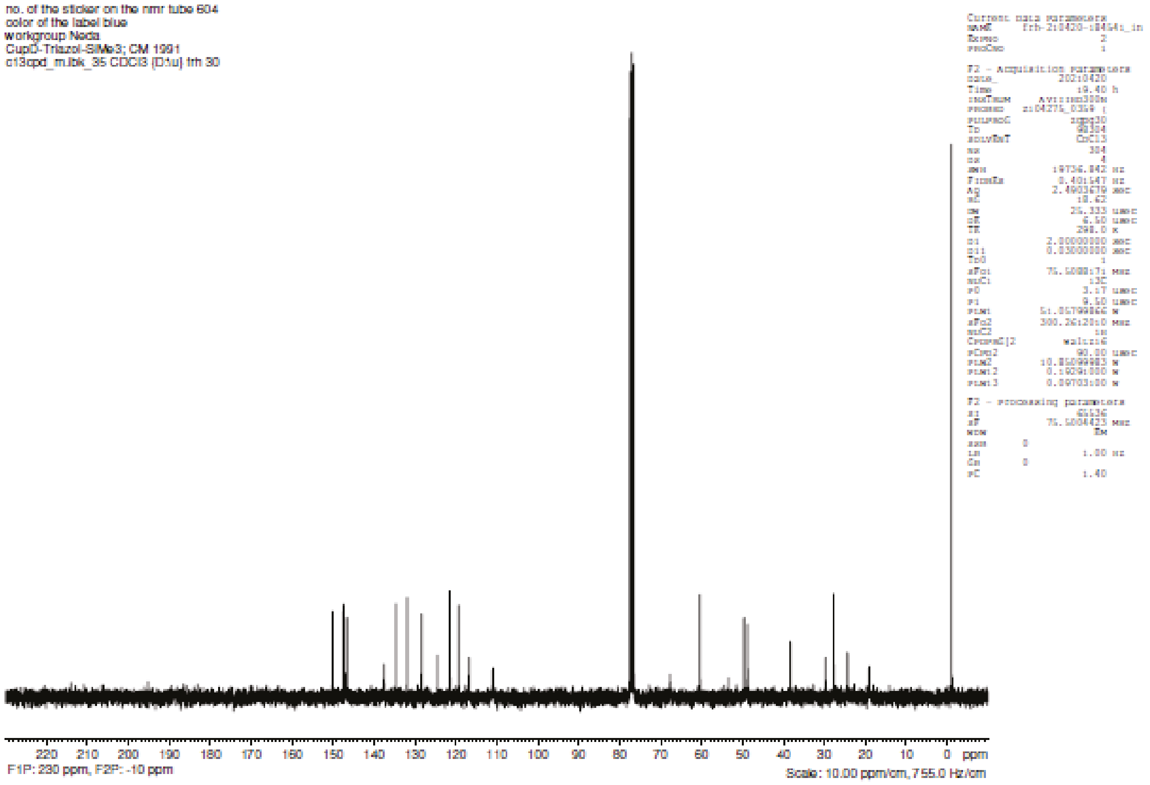

1H- and 13C-NMR experiment for (S)-(6-(1H-1,2,3-triazol-1-yl)quinolin-4-yl)((1S,2R,4S,5R)-5-vinylquinuclidin-2-yl)methanol (**15**):

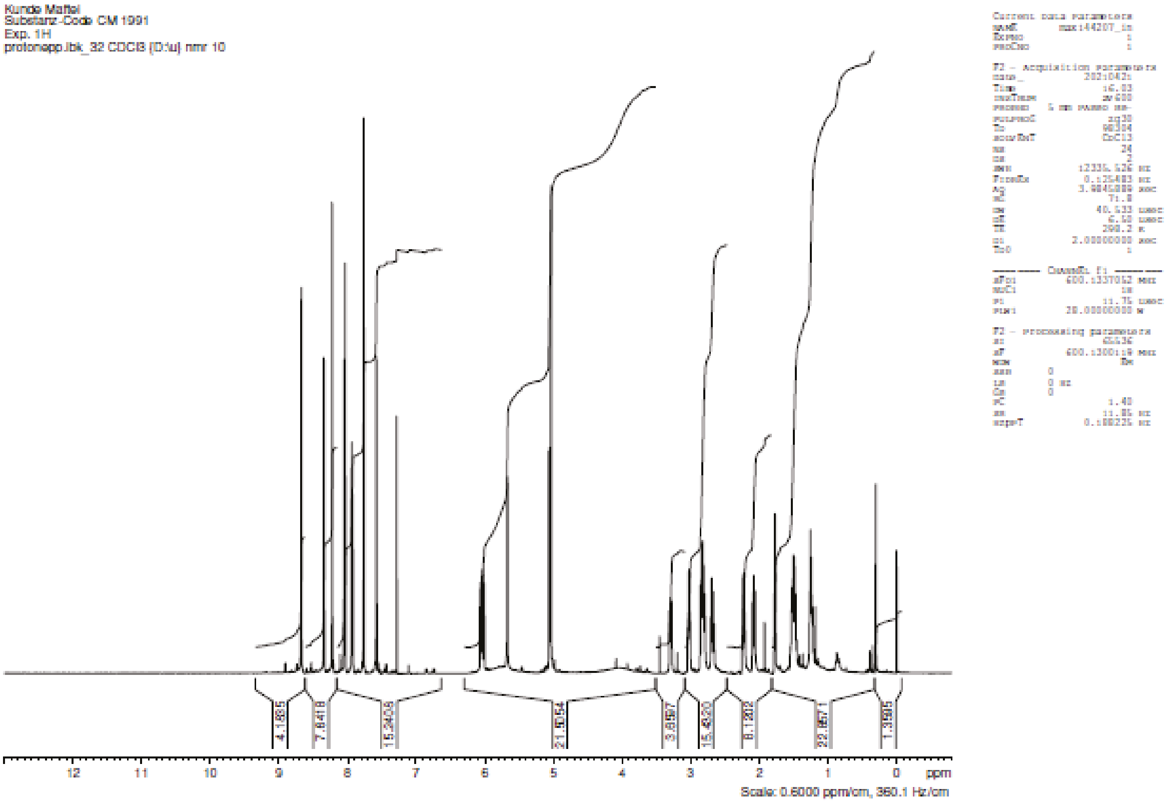

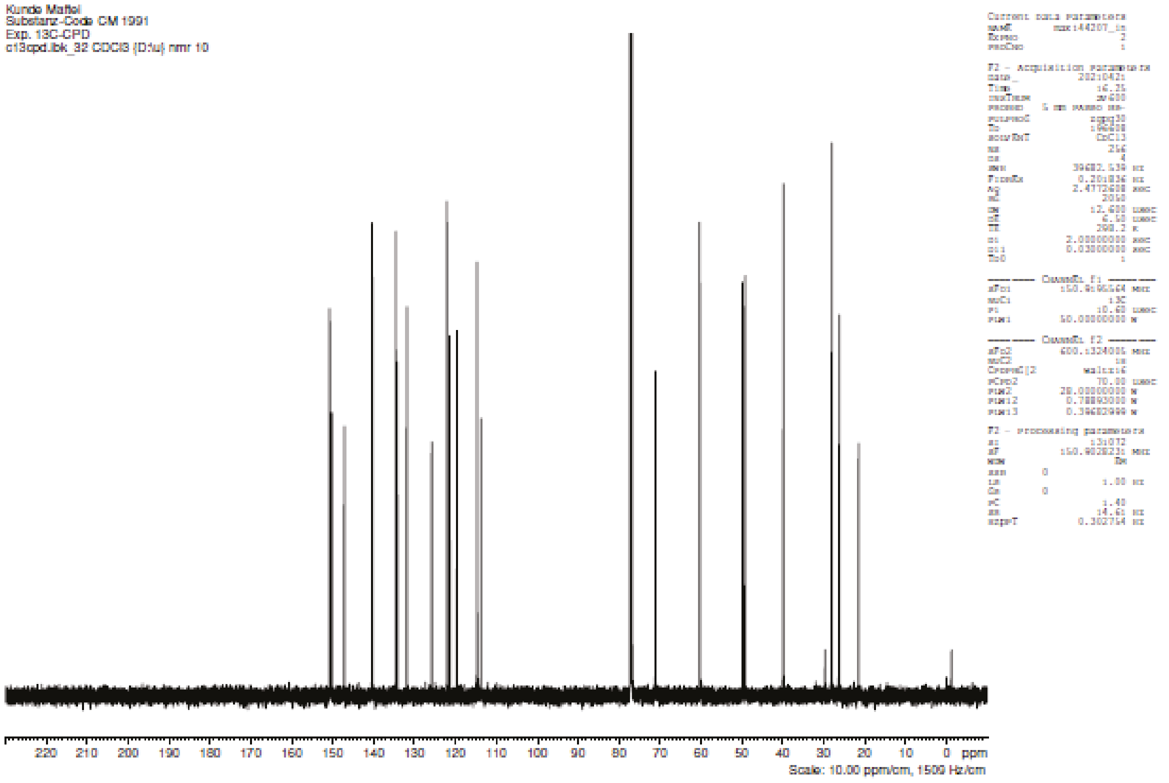

1H- and 13C-NMR experiment for (R)-(6-(4-(trimethylsilyl)-1H-1,2,3-triazol-1-yl)quinolin-4-yl)((1S,2S,4S,5R)-5-vinylquinuclidin-2-yl)methanol (**16**):

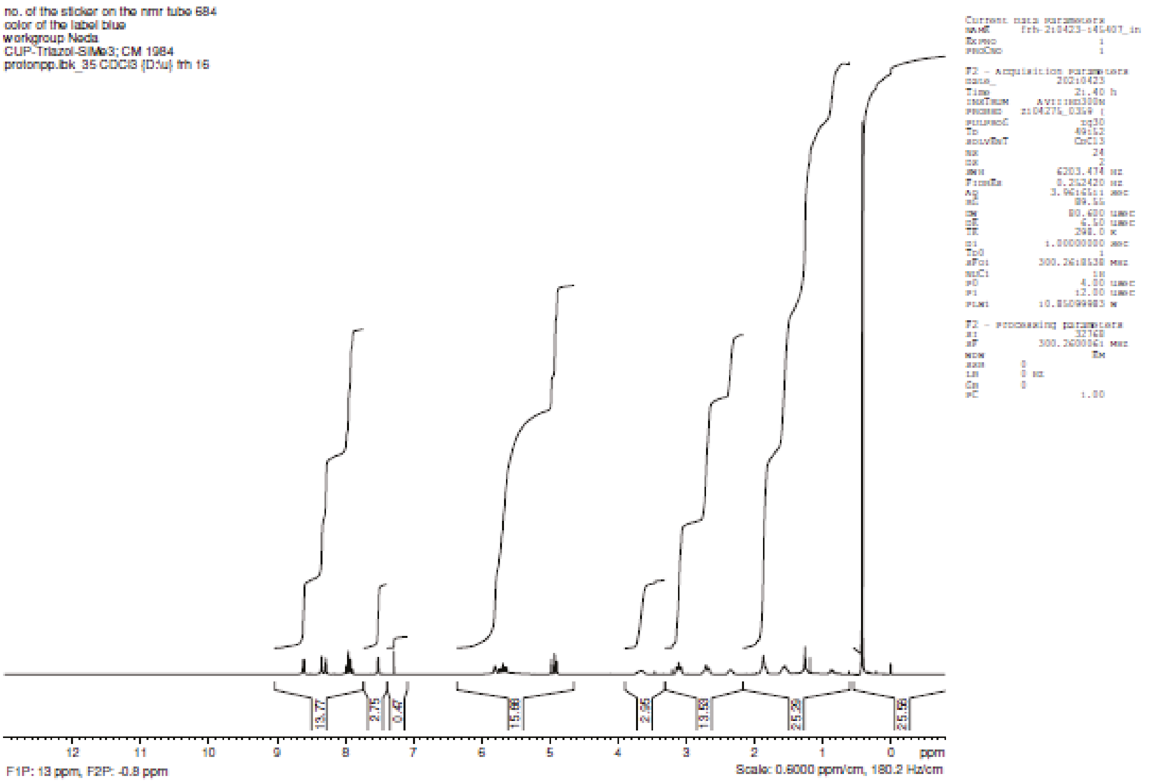

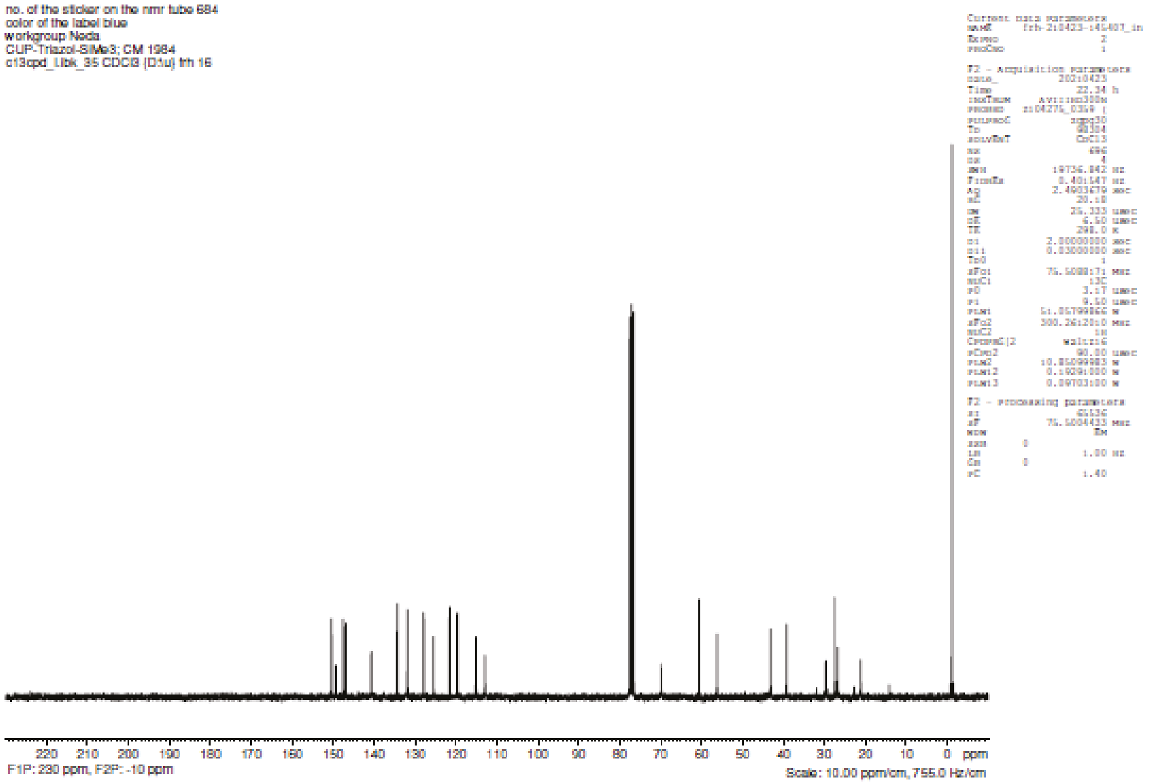

1H- and 13C-NMR experiment for (R)-(6-(1H-1,2,3-triazol-1-yl)quinolin-4-yl)((1S,2S,4S,5R)-5-vinylquinuclidin-2-yl)methanol (**17**):

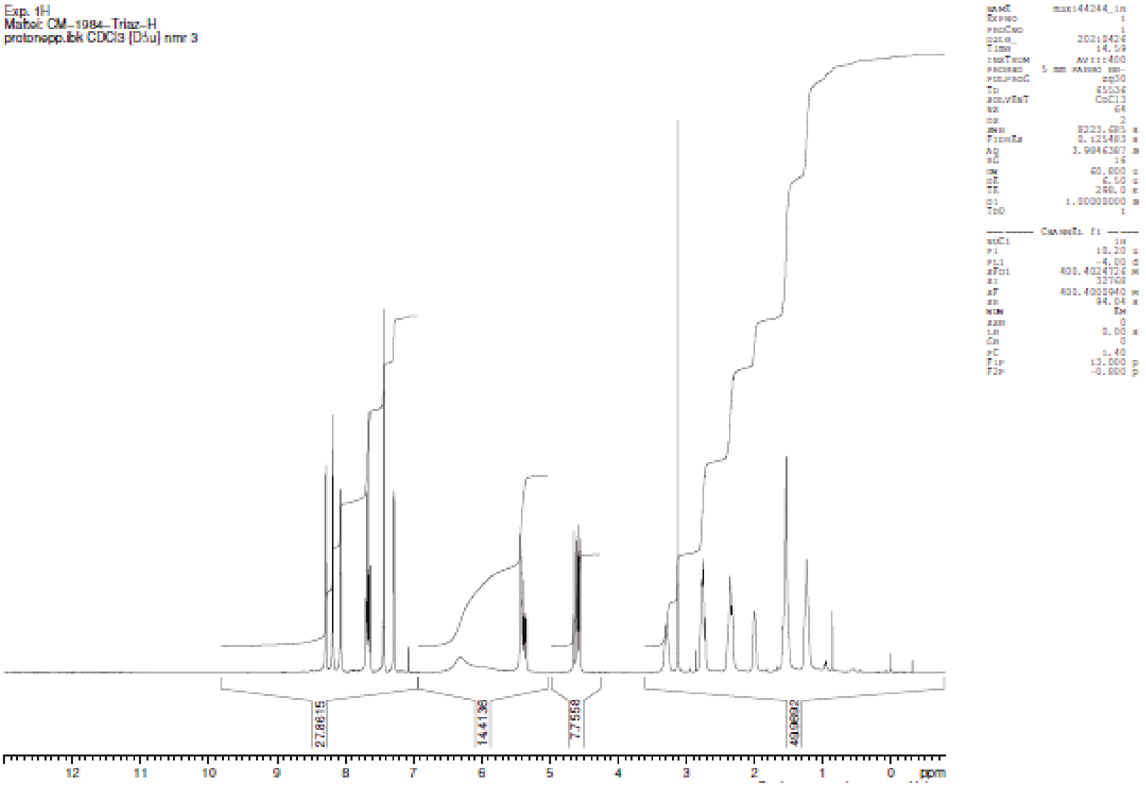

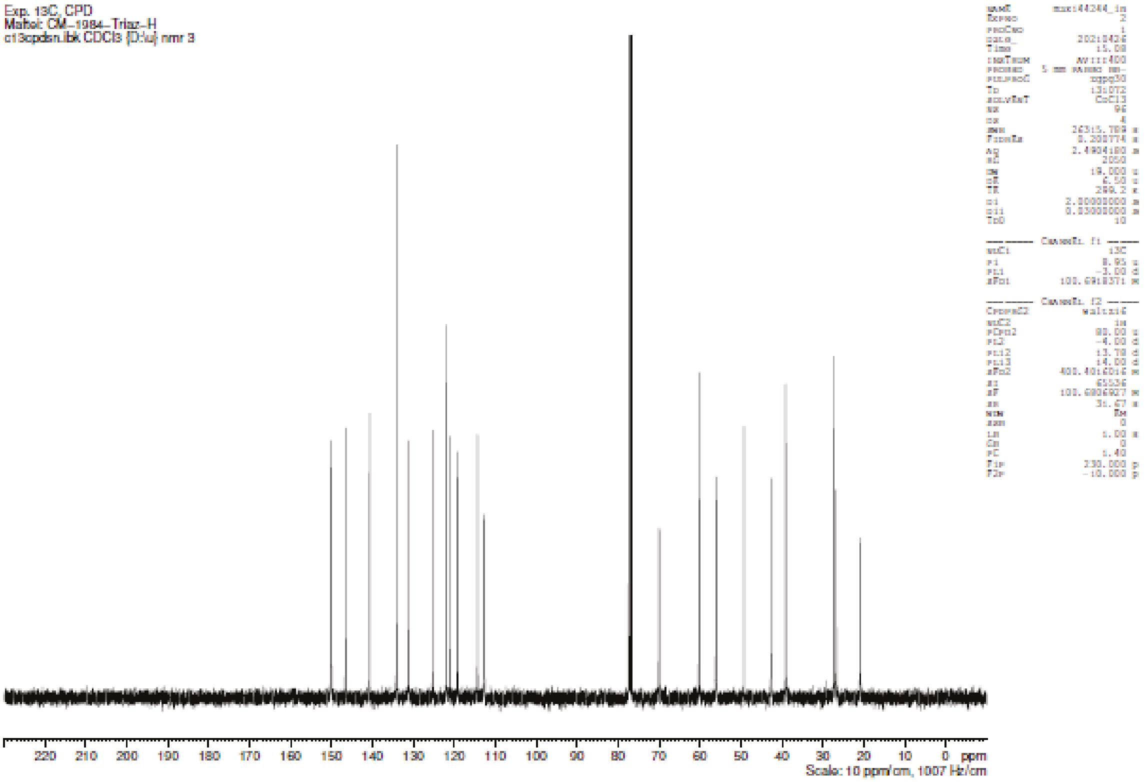

1H- and 13C-NMR experiment for (S)-(6-(4-(trimethylsilyl)-1H-1,2,3-triazol-1-yl)quinolin-4-yl)((1S,2R,4S,5R)-5-ethylquinuclidin-2-yl)methanol (**18**):

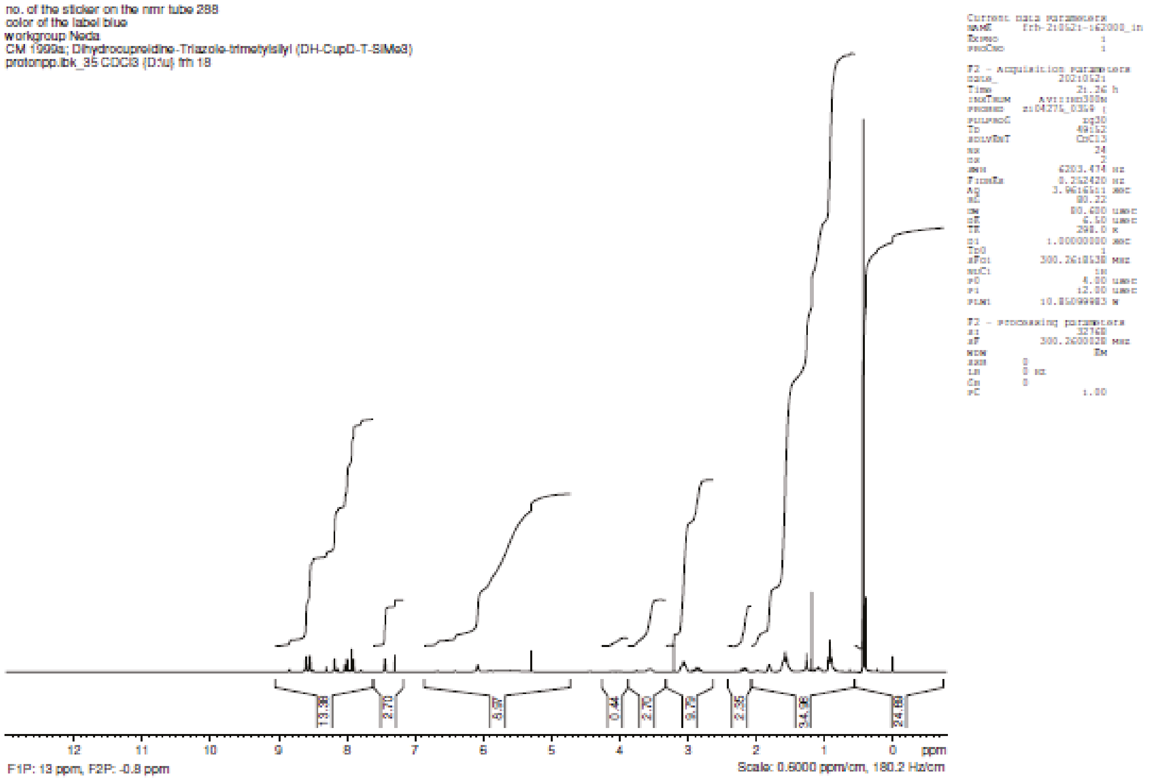

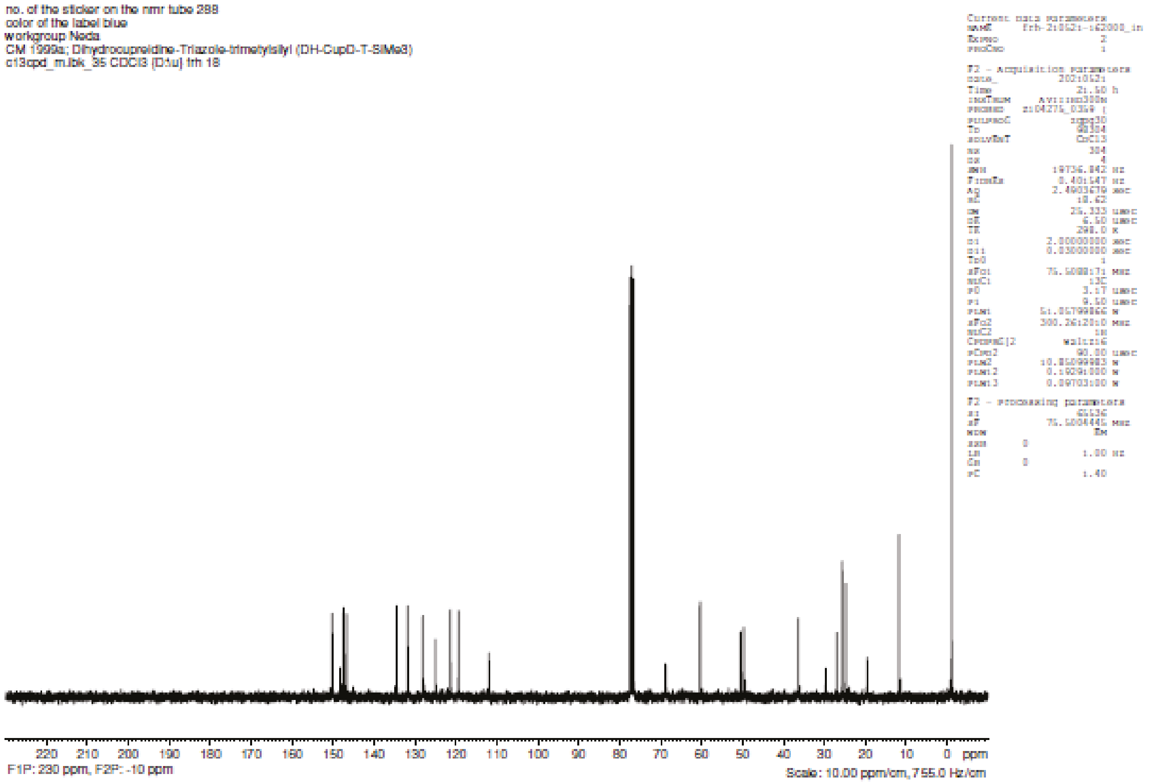

1H- and 13C-NMR experiment for (S)-(6-(1H-1,2,3-triazol-1-yl)quinolin-4-yl)((1S,2R,4S,5R)-5-ethylquinuclidin-2-yl)methanol (**19**):

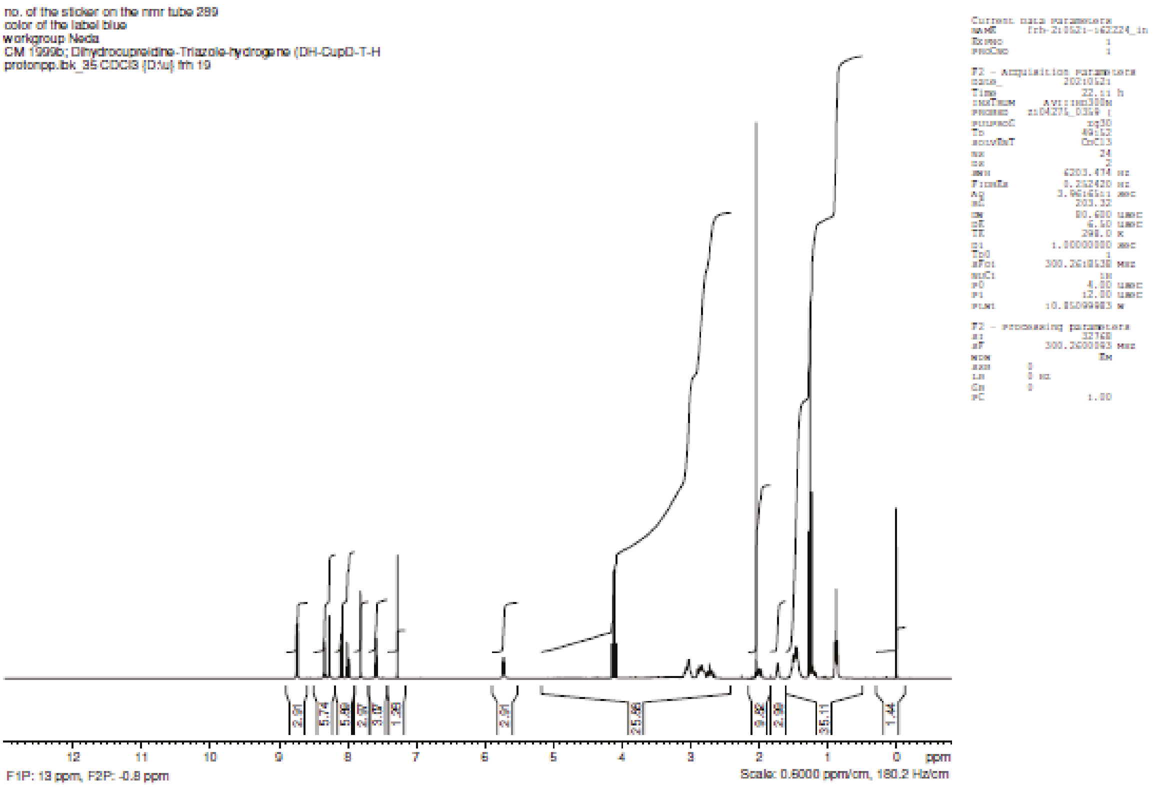

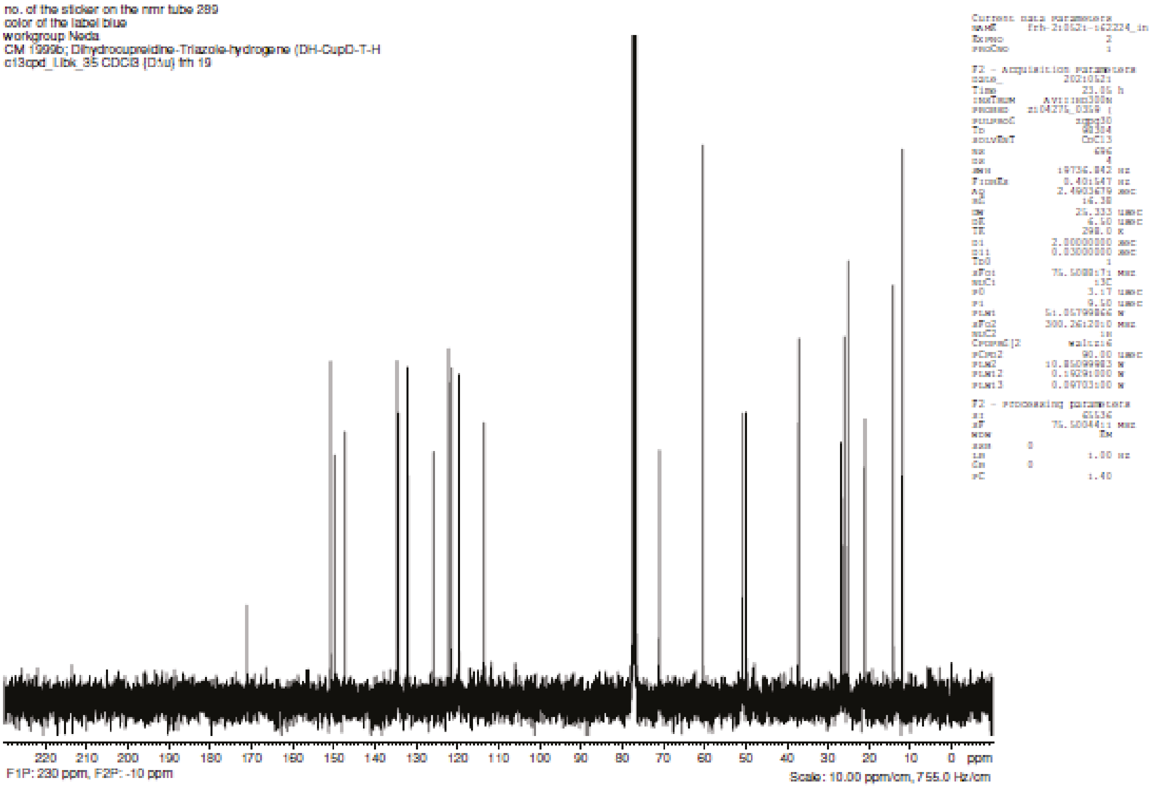

1H- and 13C-NMR experiment for (R)-(6-(4-(trimethylsilyl)-1H-1,2,3-triazol-1-yl)quinolin-4-yl)((1S,2S,4S,5R)-5-ethylquinuclidin-2-yl)methanol (**20**):

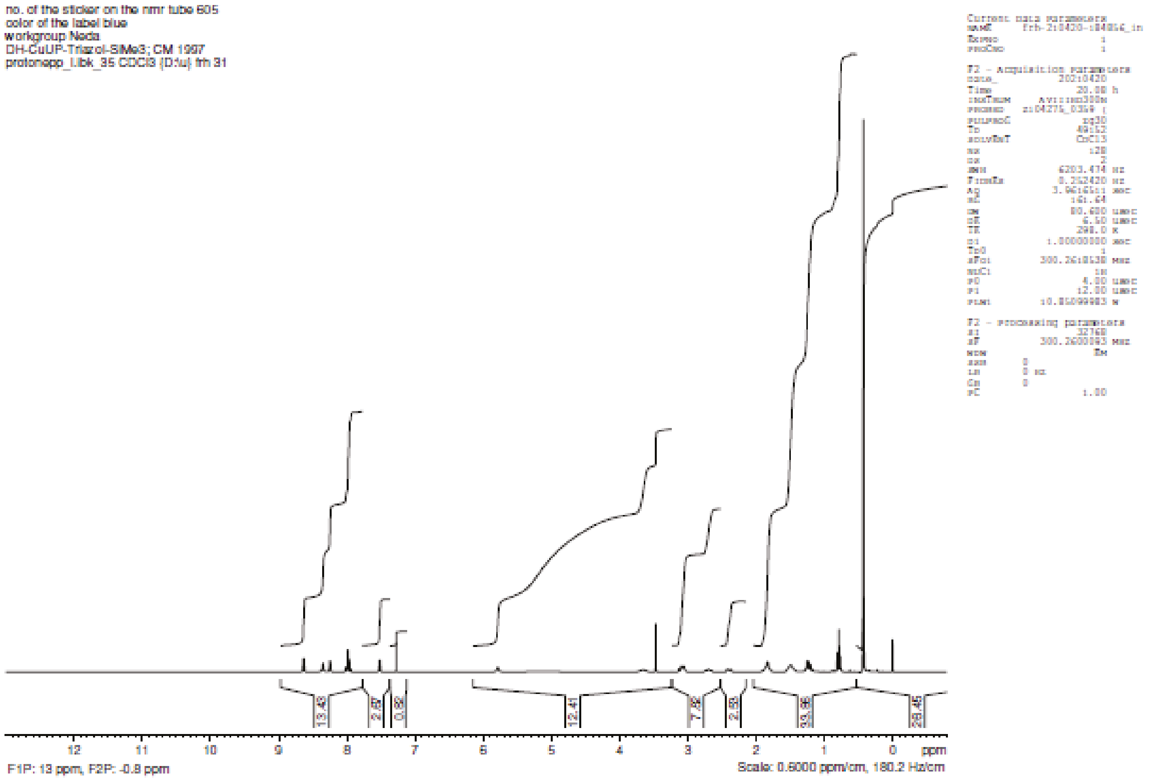

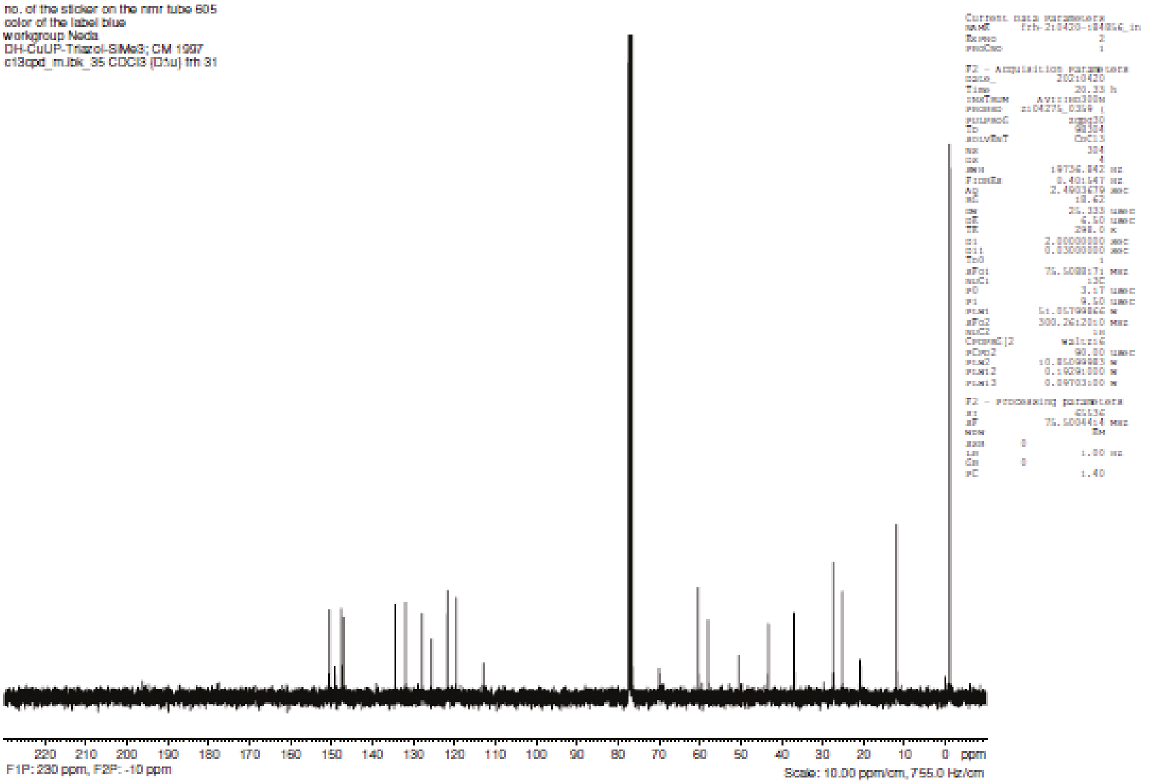

1H- and 13C-NMR experiment for (R)-(6-(1H-1,2,3-triazol-1-yl)quinolin-4-yl)((1S,2S,4S,5R)-5-ethylquinuclidin-2-yl)methanol (**21**):

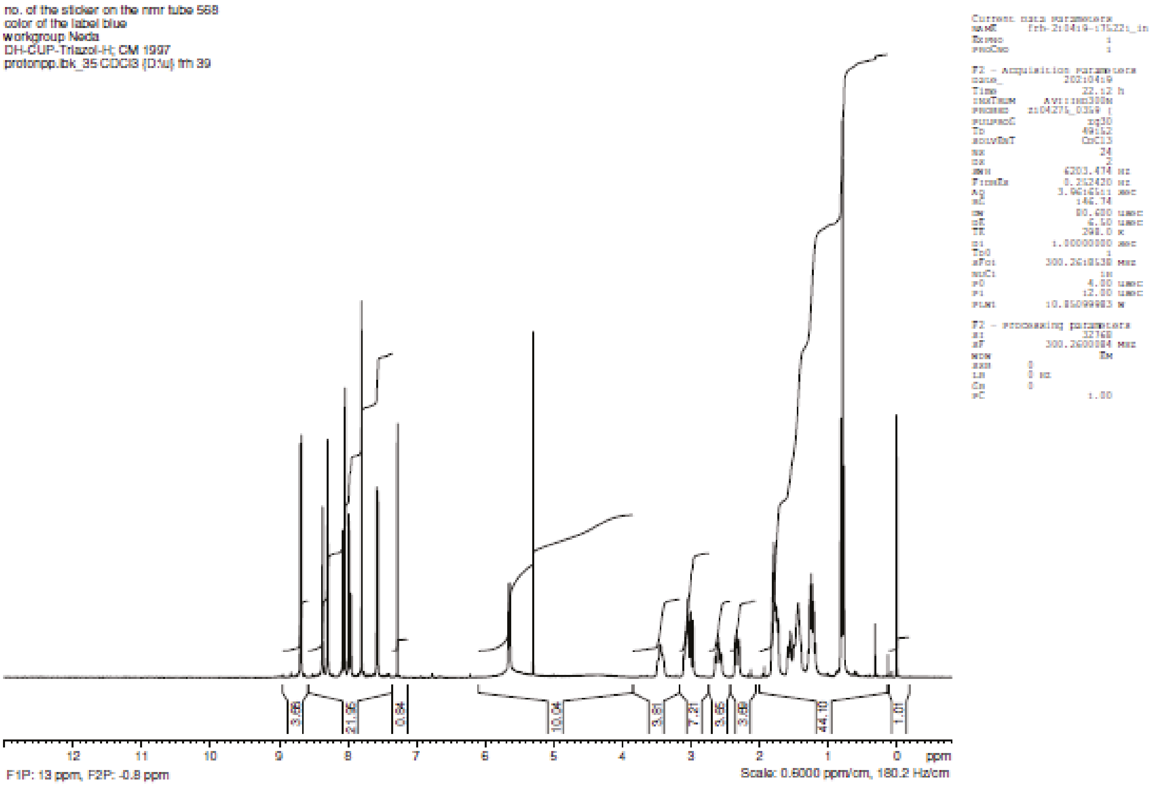

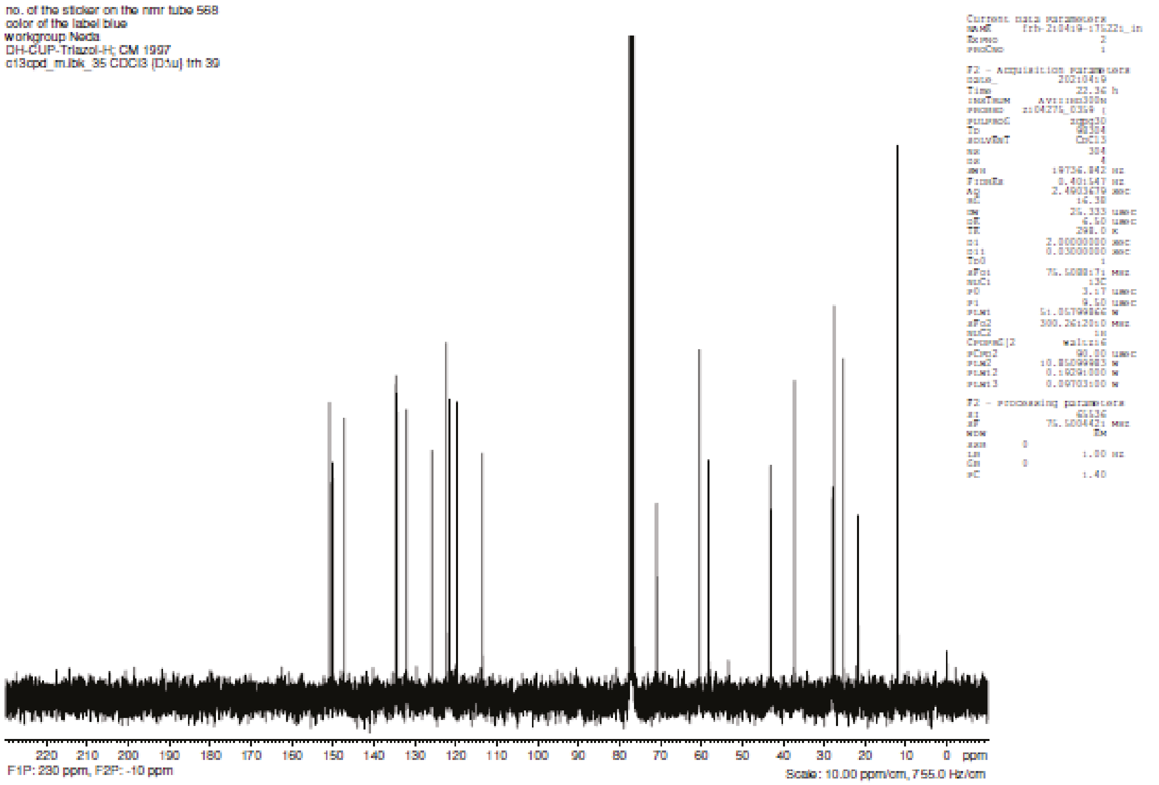

1H- and 13C-NMR experiment for (S)-(6-(4-((1S,3R,4S,6R)-6-(hydroxymethyl)quinuclidin-3-yl)-1H-1,2,3-triazol-1-yl)quinolin-4-yl)((1S,2R,4S,5R)-5-vinylquinuclidin-2-yl)methanol (**24**):

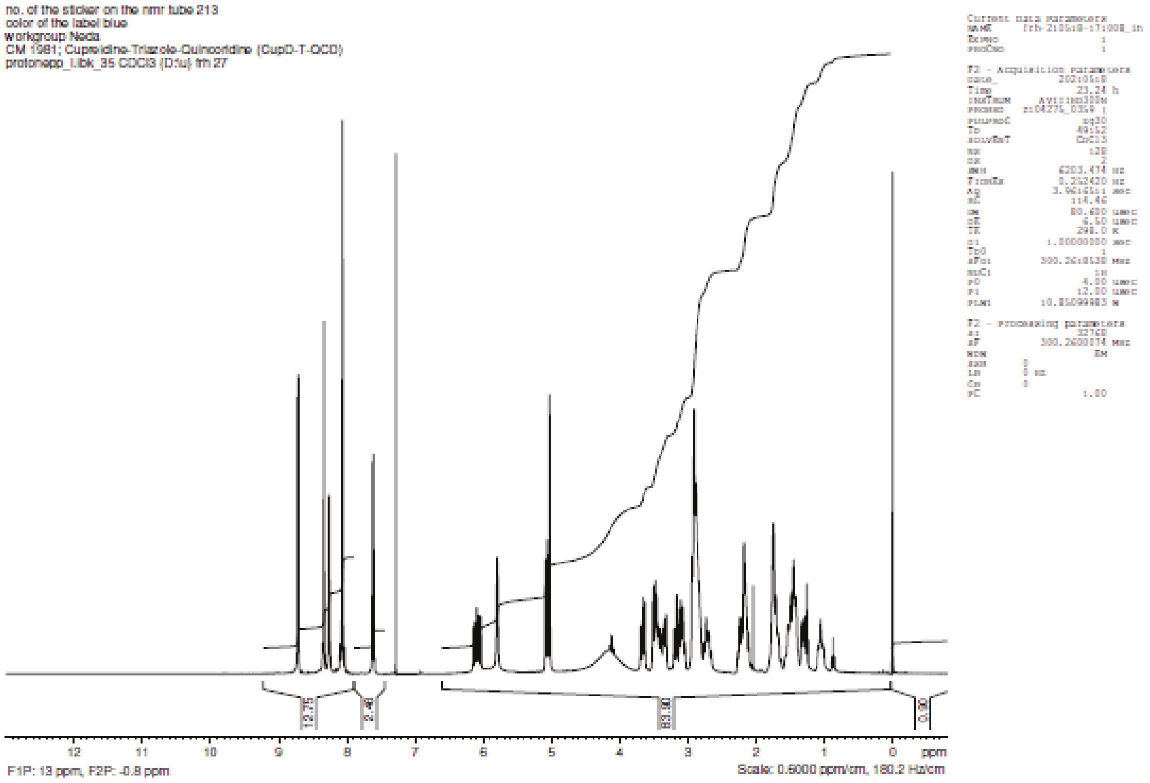

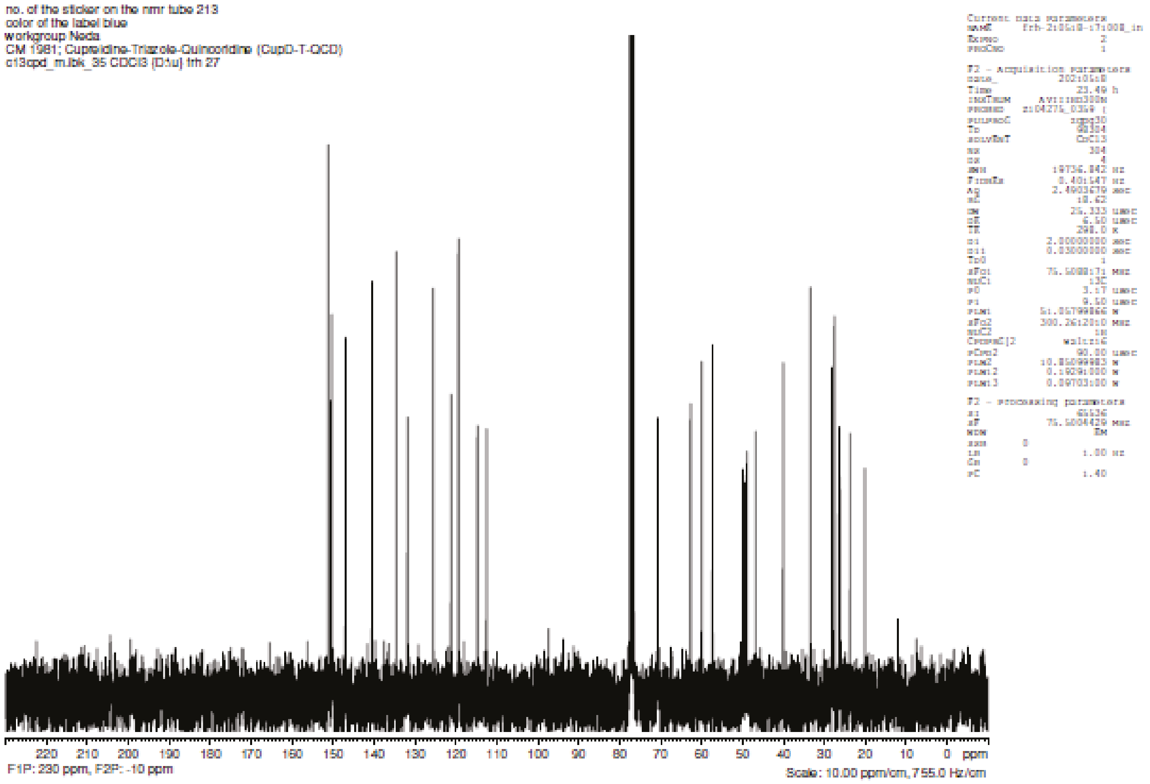

1H- and 13C-NMR experiment for (S)-(6-(4-((1S,3R,4S,6S)-6-(hydroxymethyl)quinuclidin-3-yl)-1H-1,2,3-triazol-1-yl)quinolin-4-yl)((1S,2R,4S,5R)-5-vinylquinuclidin-2-yl)methanol (**25**):

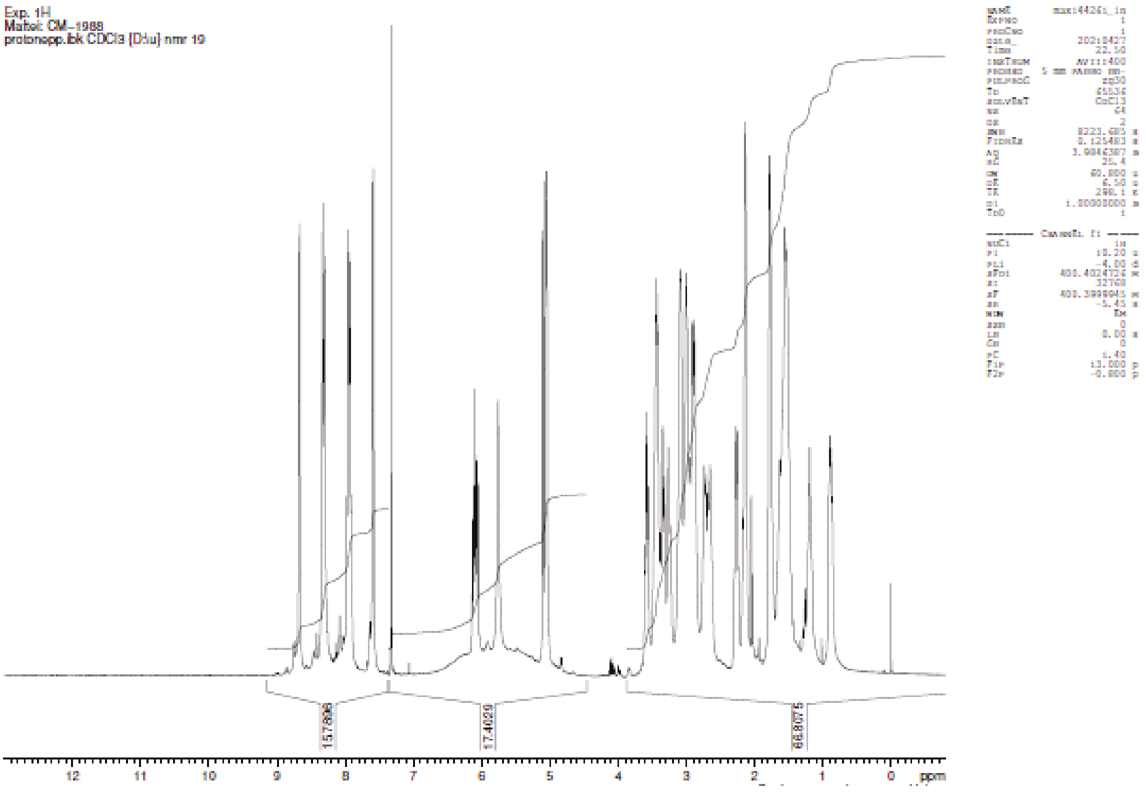

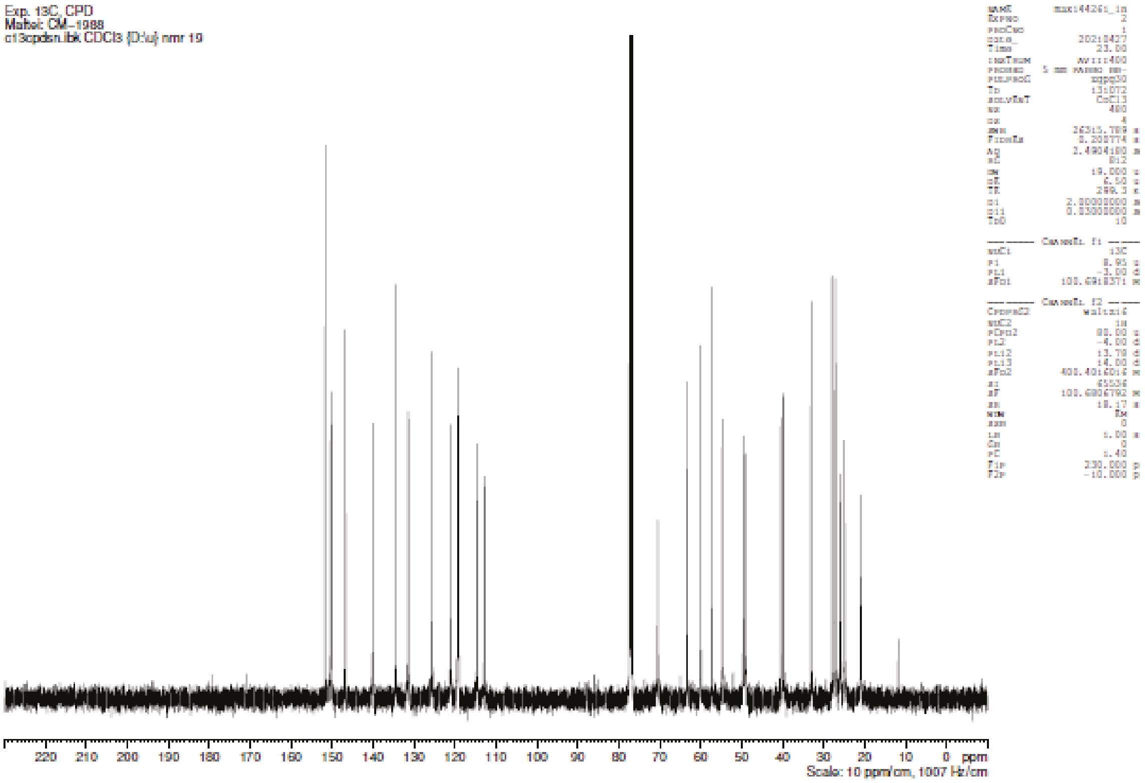

1H- and 13C-NMR experiment for (R)-(6-(4-((1S,3R,4S,6R)-6-(hydroxymethyl)quinuclidin-3-yl)-1H-1,2,3-triazol-1-yl)quinolin-4-yl)((1S,2S,4S,5R)-5-vinylquinuclidin-2-yl)methanol (**26**):

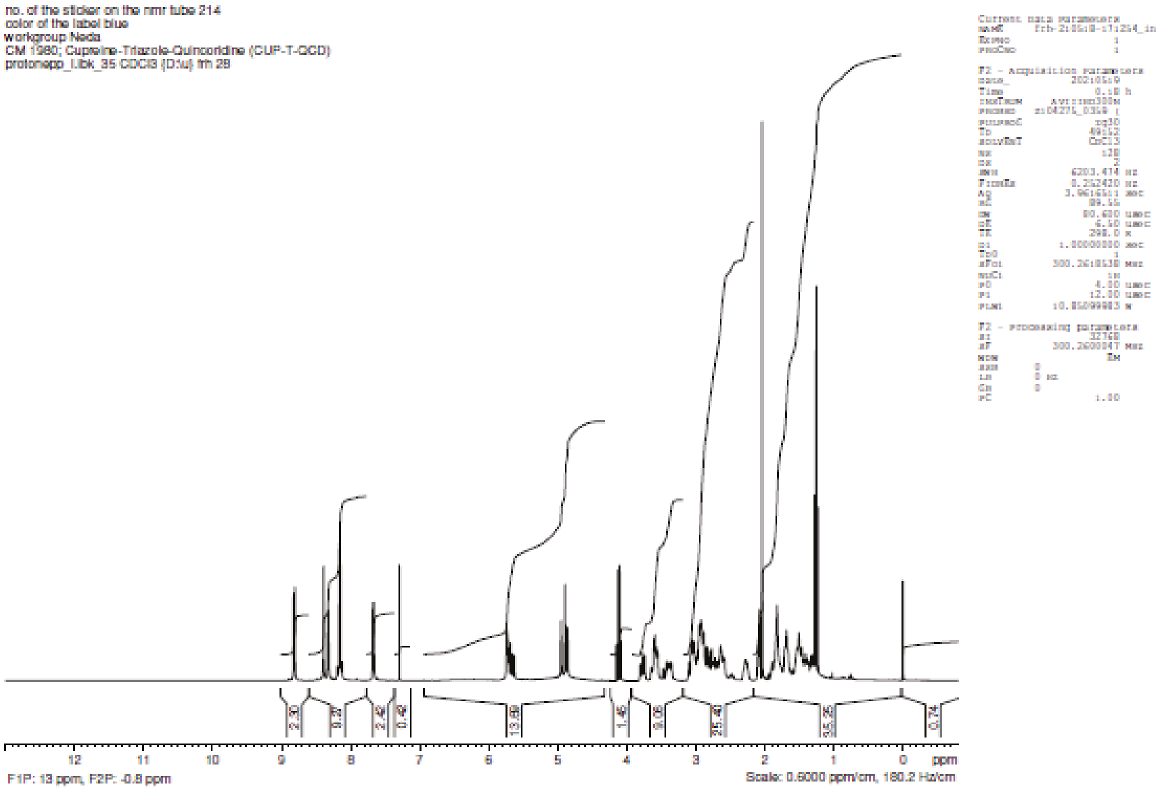

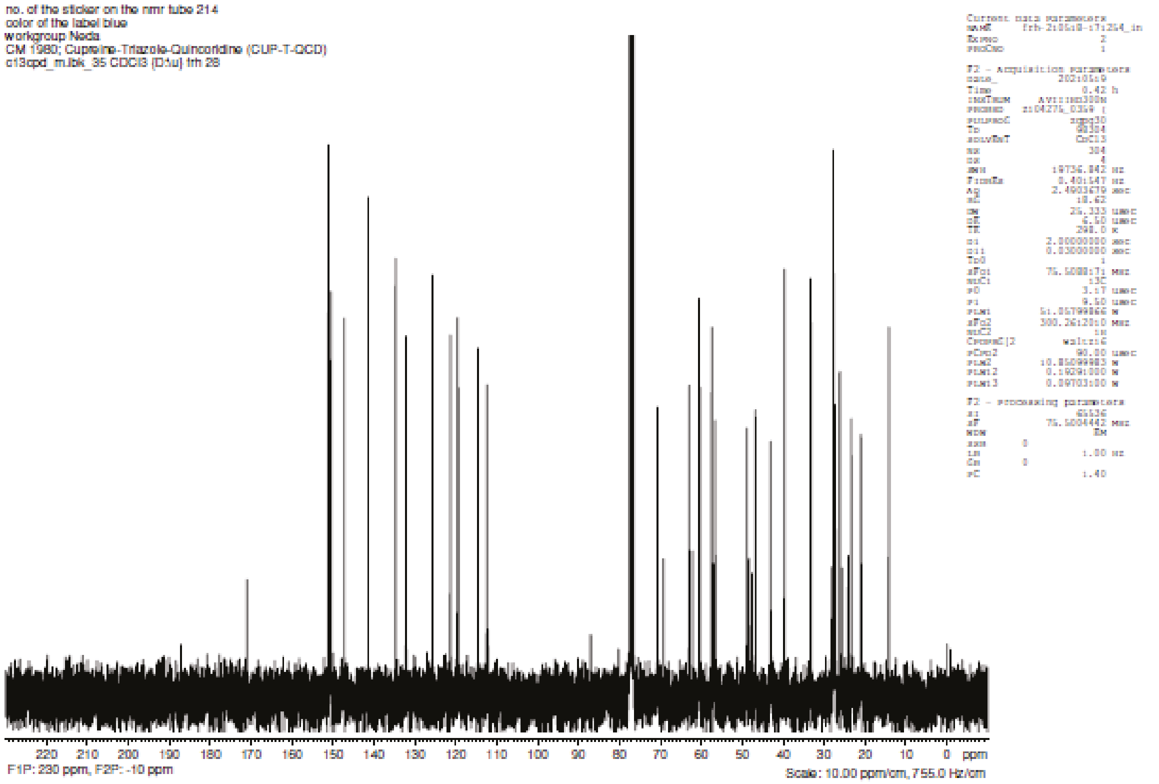

1H- and 13C-NMR experiment for (R)-(6-(4-((1S,3R,4S,6S)-6-(hydroxymethyl)quinuclidin-3-yl)-1H-1,2,3-triazol-1-yl)quinolin-4-yl)((1S,2S,4S,5R)-5-vinylquinuclidin-2-yl)methanol (**27**):

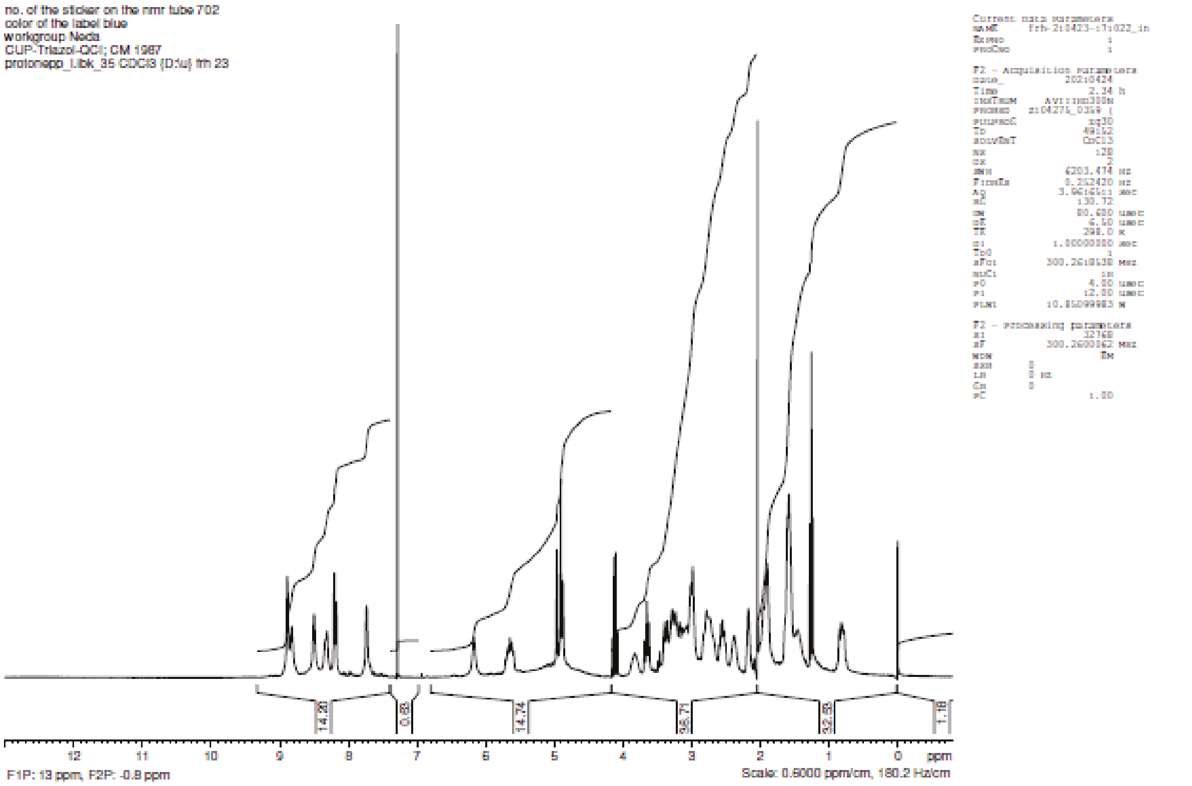

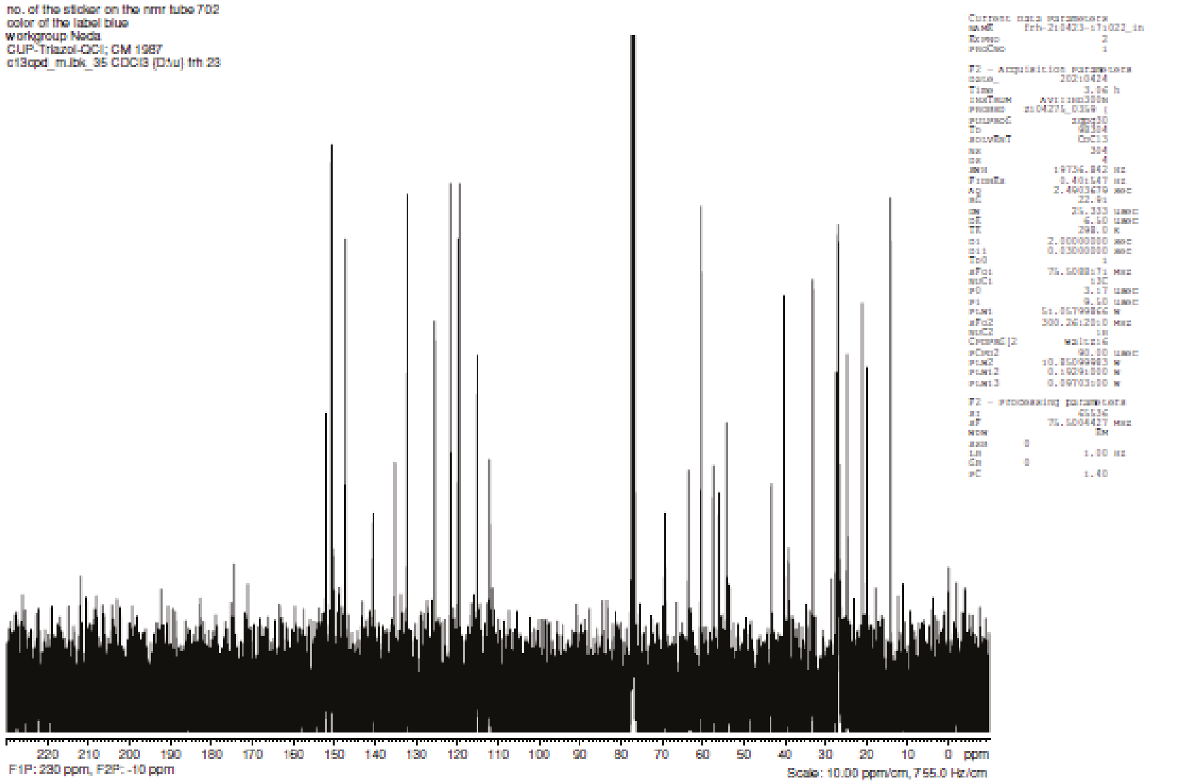

1H- and 13C-NMR experiment for (S)-(6-(4-((1S,3R,4S,6R)-6-(hydroxymethyl)quinuclidin-3-yl)-1H-1,2,3-triazol-1-yl)quinolin-4-yl)((1S,2R,4S,5R)-5-ethylquinuclidin-2-yl)methanol (**28**):

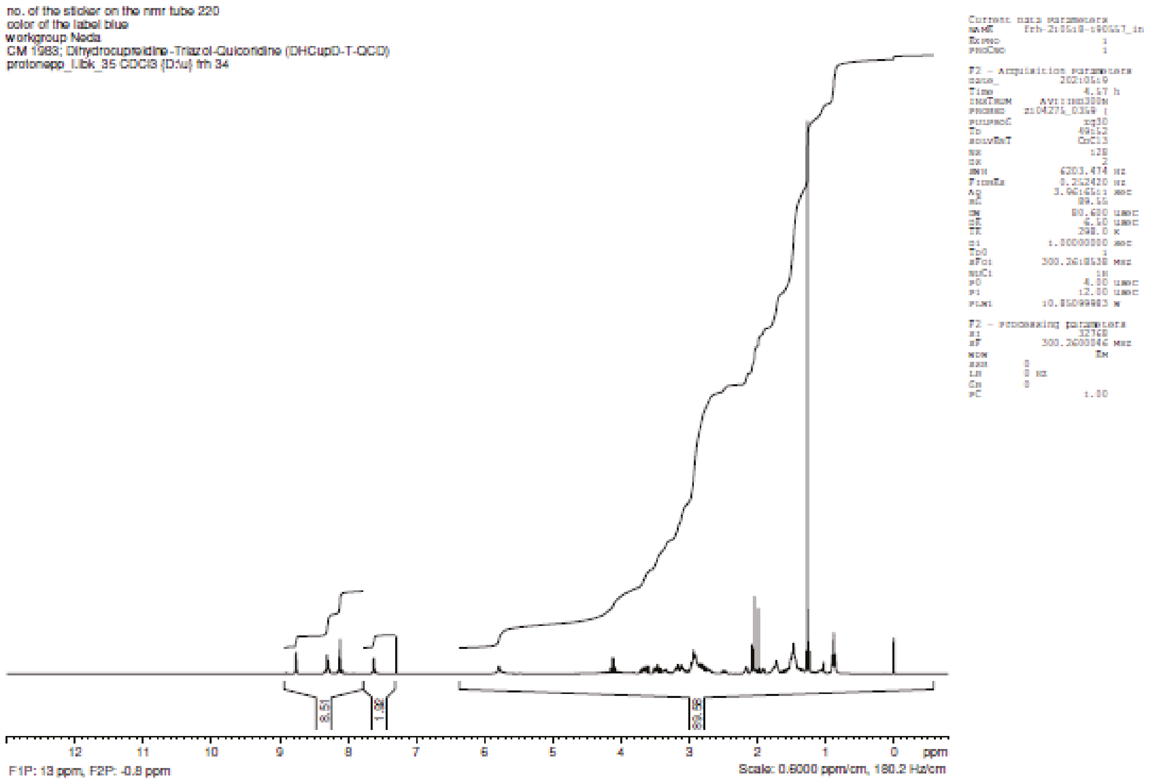

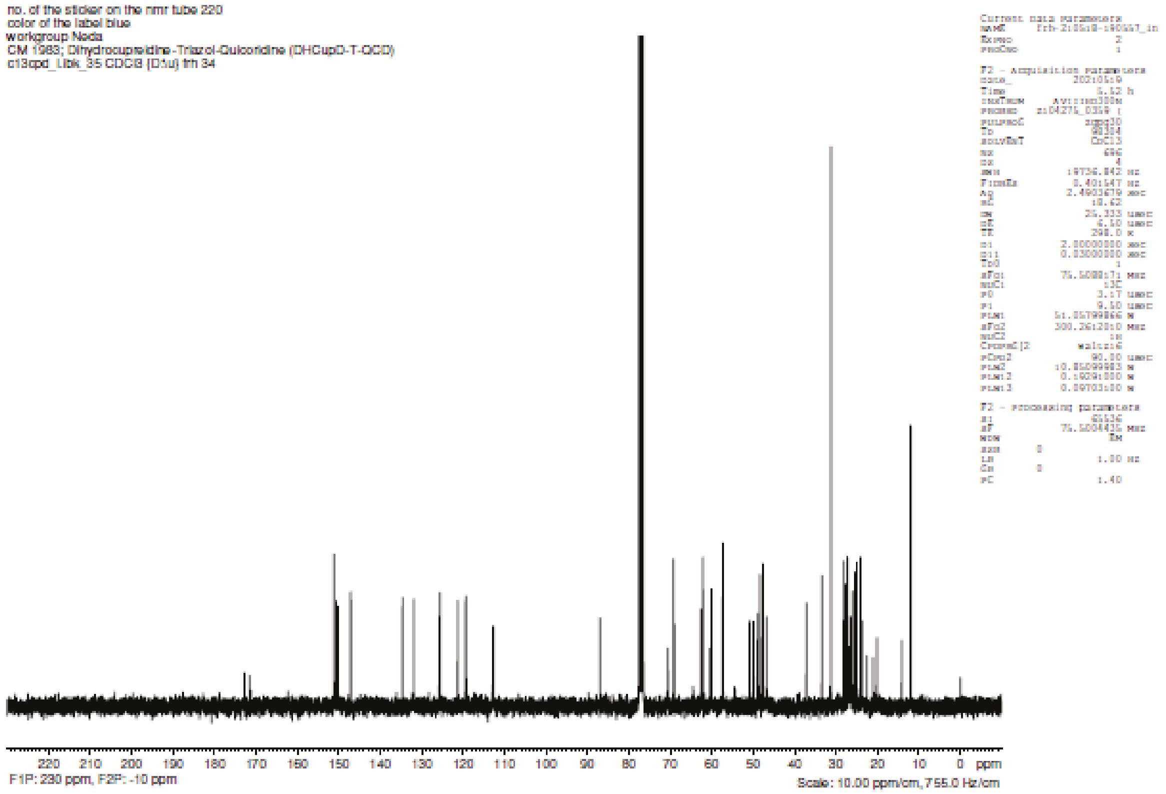

1H- and 13C-NMR experiment for (R)-(6-(4-((1S,3R,4S,6R)-6-(hydroxymethyl)quinuclidin-3-yl)-1H-1,2,3-triazol-1-yl)quinolin-4-yl)((1S,2S,4S,5R)-5-ethylquinuclidin-2-yl)methanol (**30**):

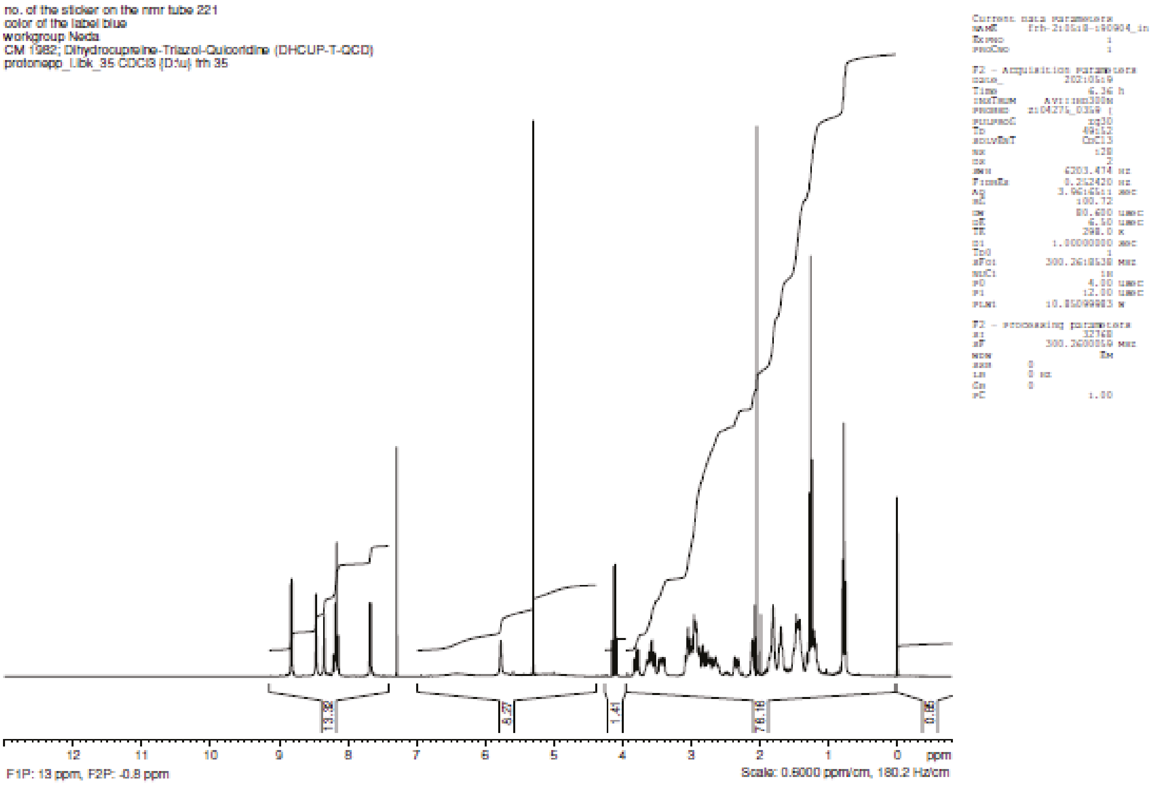

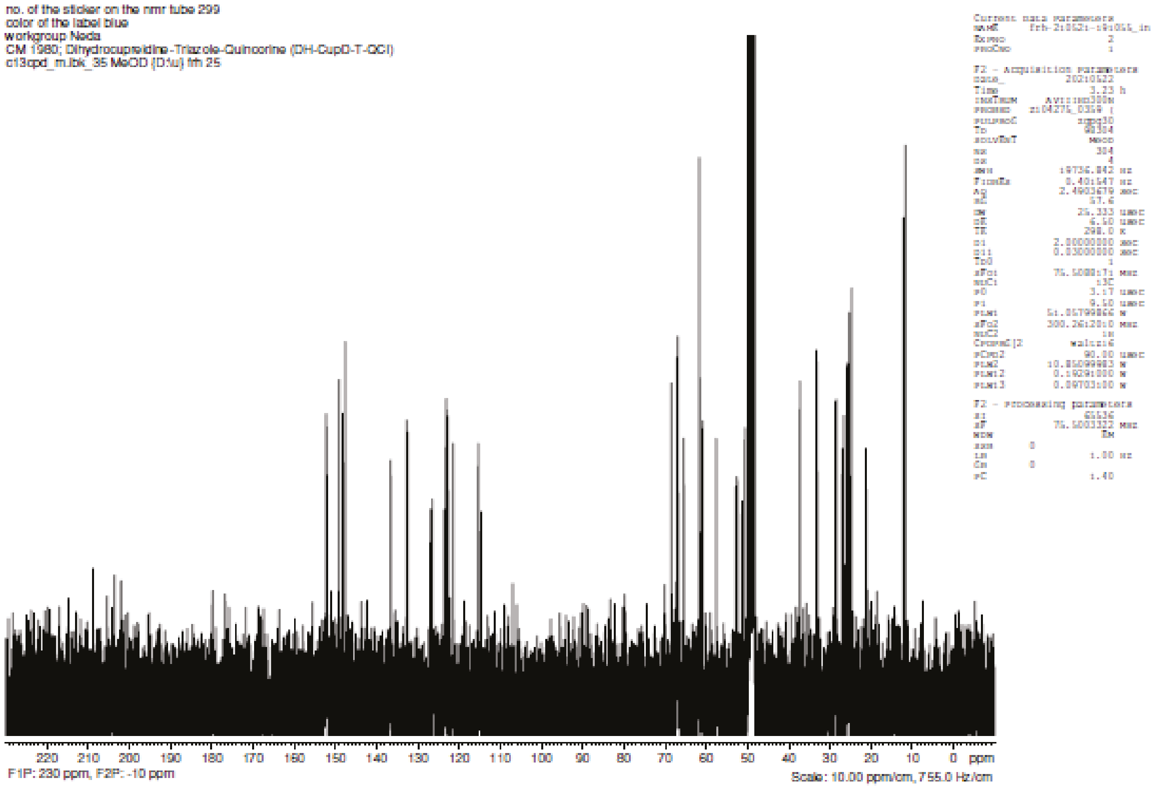

1H- and 13C-NMR experiment for (R)-(6-(4-((1S,3R,4S,6S)-6-(hydroxymethyl)quinuclidin-3-yl)-1H-1,2,3-triazol-1-yl)quinolin-4-yl)((1S,2S,4S,5R)-5-ethylquinuclidin-2-yl)methanol (**31**):

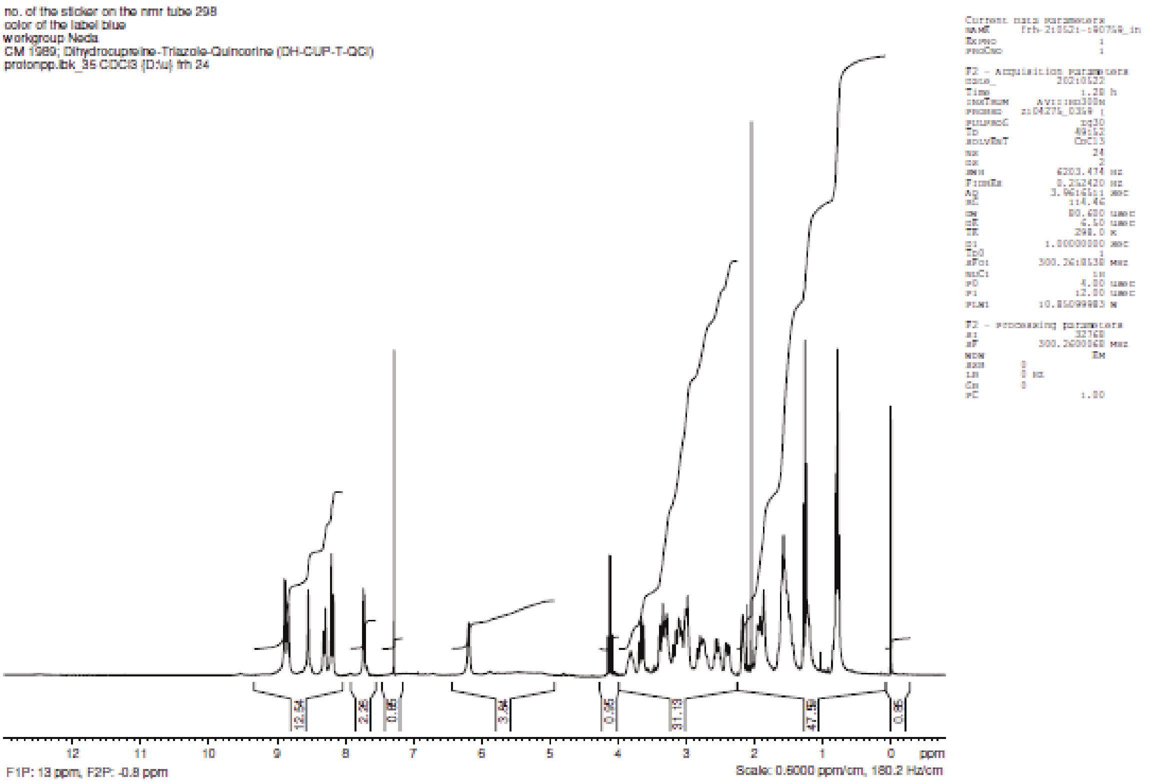

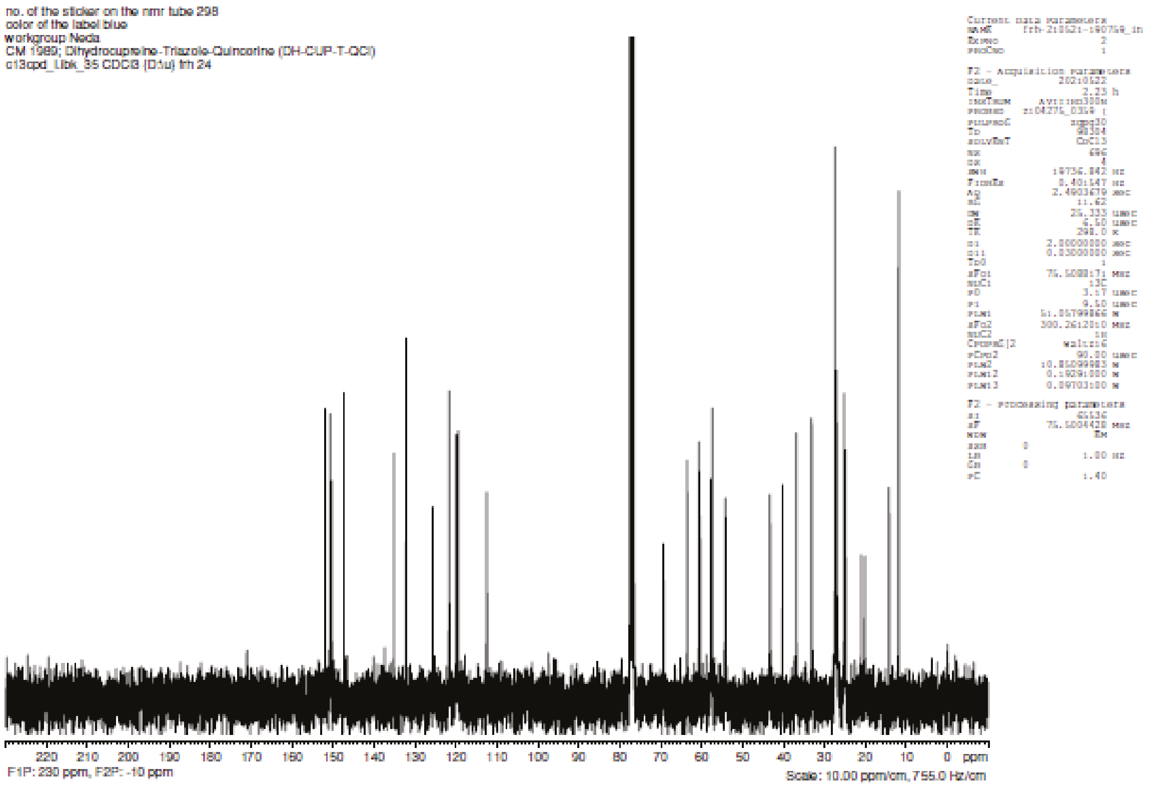

IR spectroscopy

IR spectrogram for (S)-(6-azidoquinolin-4-yl)((1S,2R,4S,5R)-5-vinylquinuclidin-2-yl)methanol (**8**):

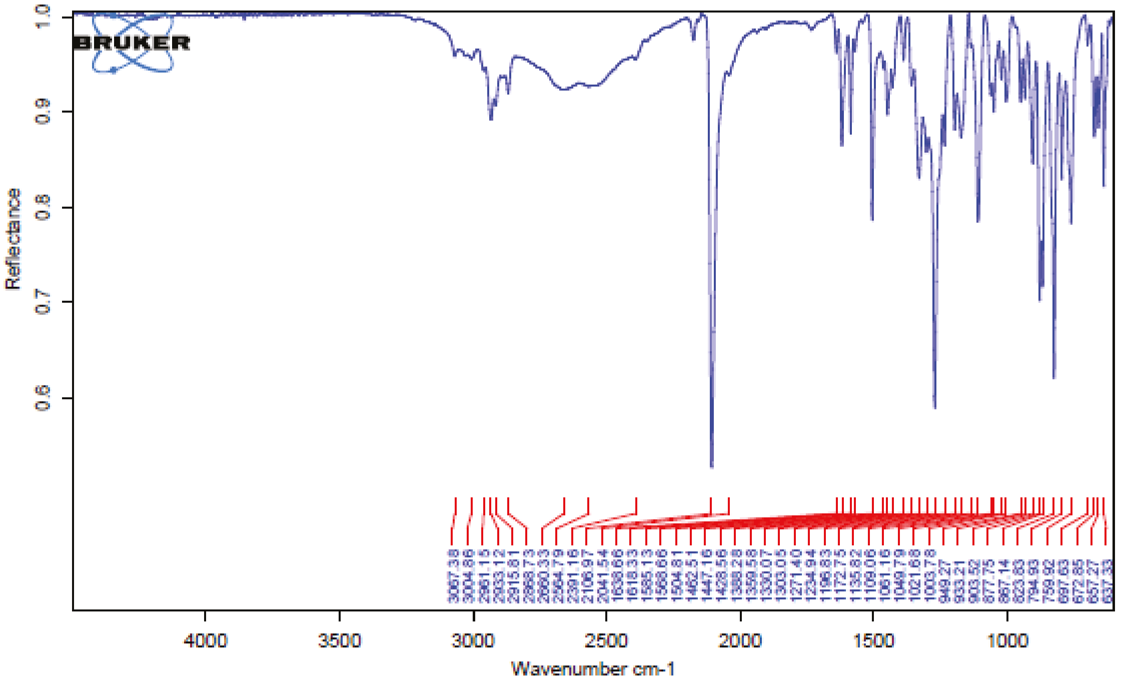

IR spectrogram for (R)-(6-azidoquinolin-4-yl)((1S,2S,4S,5R)-5-vinylquinuclidin-2-yl)methanol (**9**):

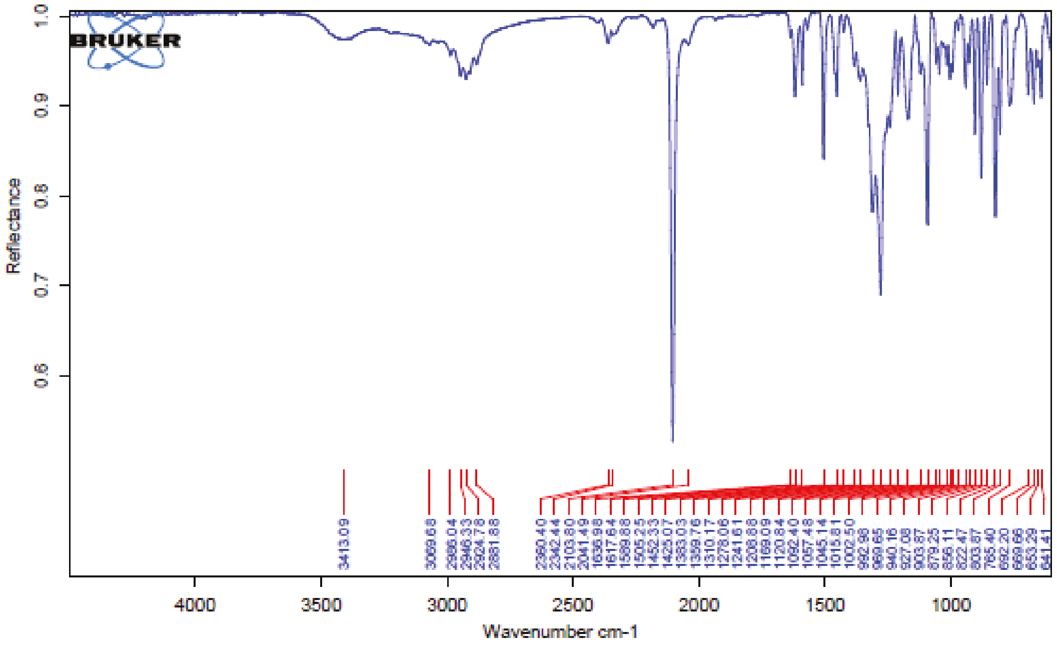

IR spectrogram for (S)-(6-azidoquinolin-4-yl)((1S,2R,4S,5R)-5-ethylquinuclidin-2-yl)methanol (**10**):

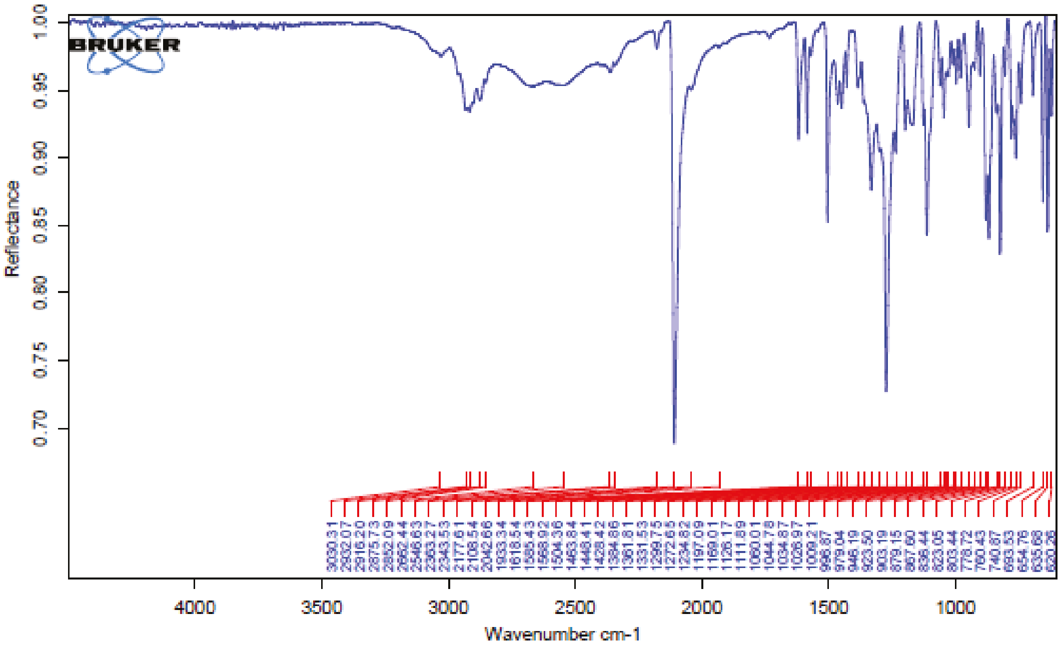

IR spectrogram for (R)-(6-azidoquinolin-4-yl)((1S,2S,4S,5R)-5-ethylquinuclidin-2-yl)methanol (**11**):

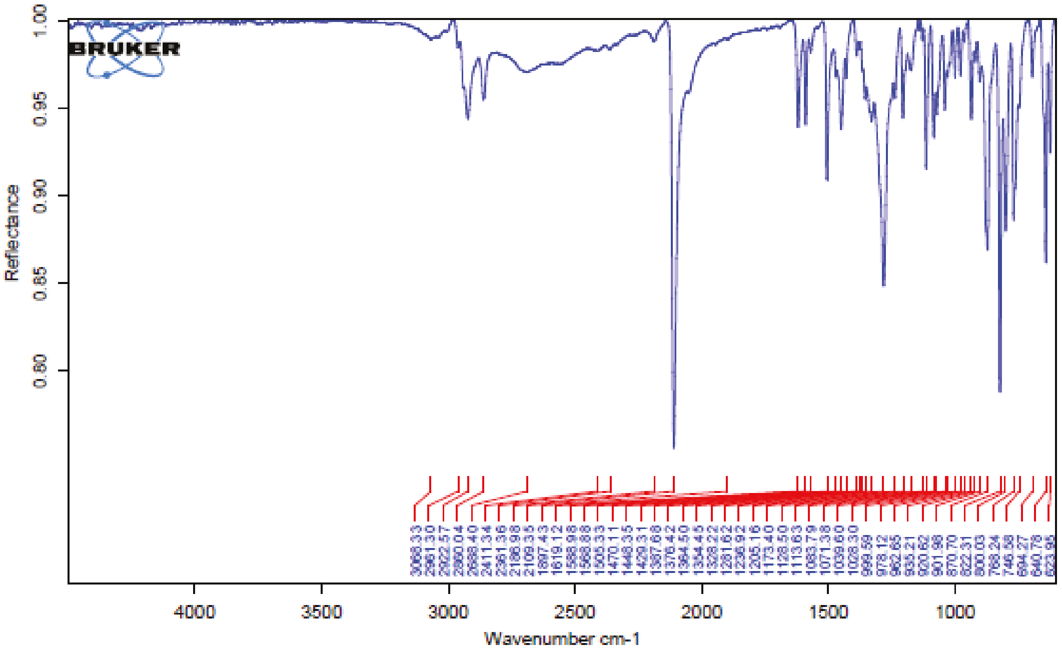

IR spectrogram for (S)-(6-azidoquinolin-4-yl)((1S,2R,4S,5S)-5-ethynylquinuclidin-2-yl)methanol (**12**):

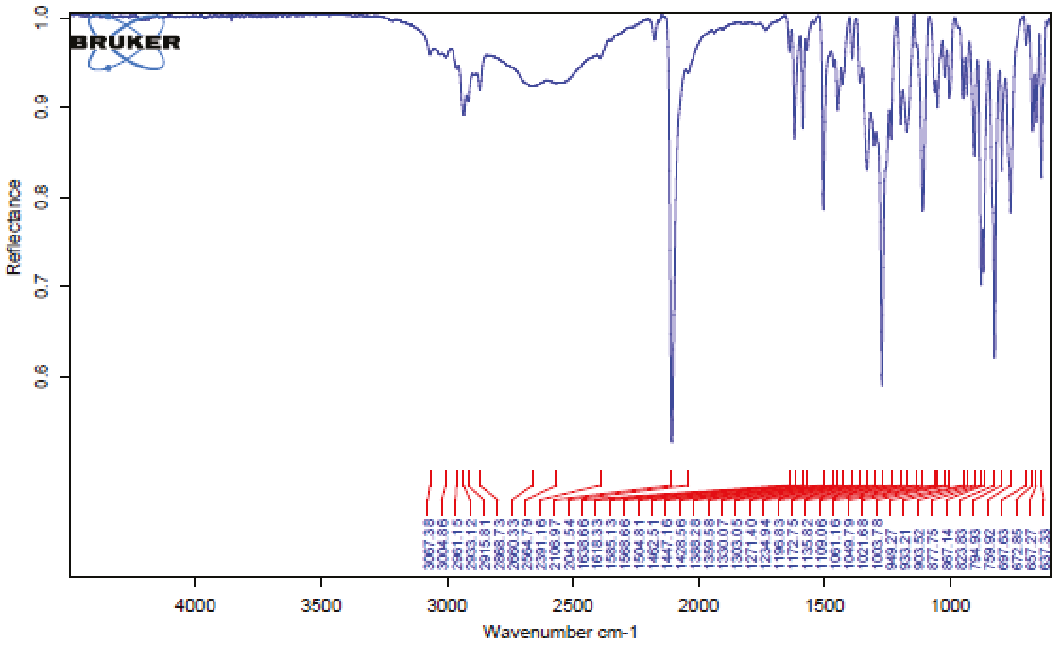

IR spectrogram for (R)-(6-azidoquinolin-4-yl)((1S,2S,4S,5S)-5-ethynylquinuclidin-2-yl)methanol (**13**):

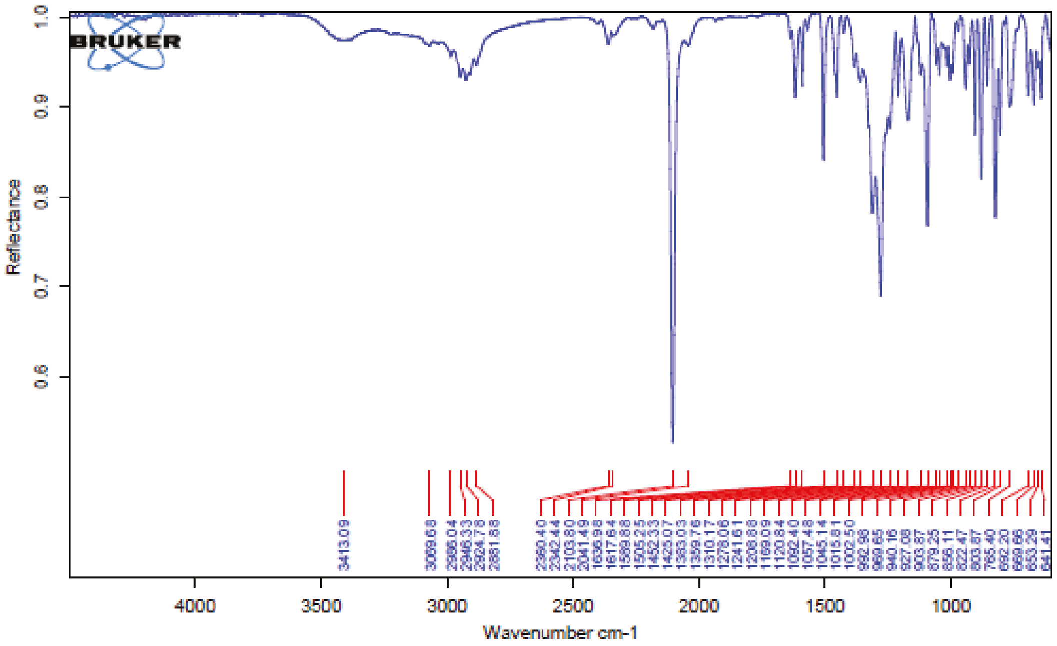

IR spectrogram for (S)-(6-(4-(trimethylsilyl)-1H-1,2,3-triazol-1-yl)quinolin-4-yl)((1S,2R,4S,5R)-5-vinylquinuclidin-2-yl)methanol (**14**):

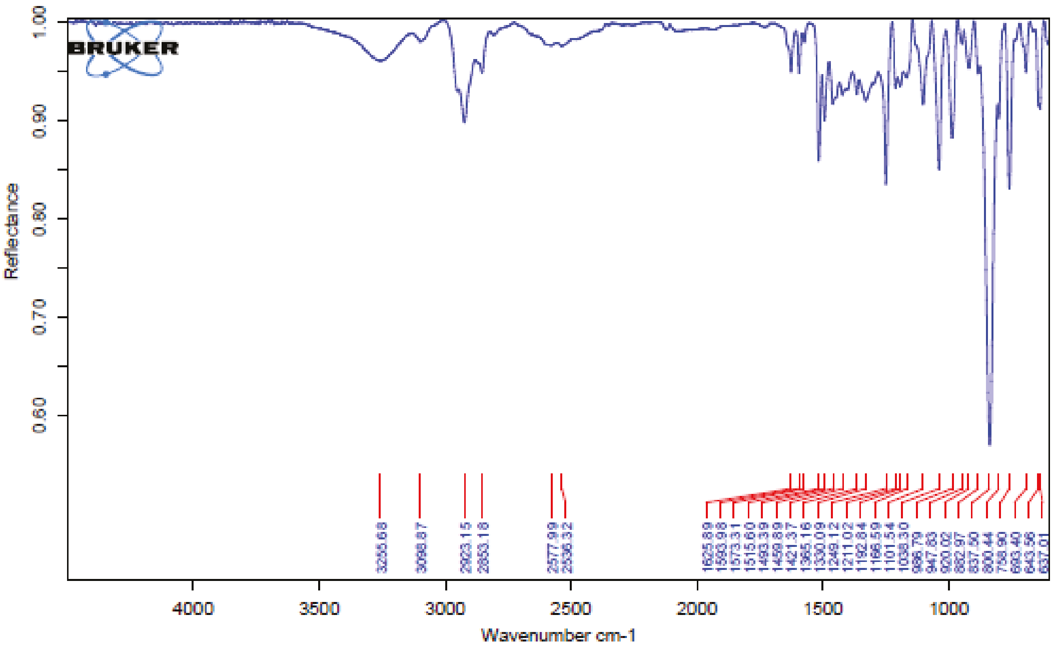

IR spectrogram for (S)-(6-(1H-1,2,3-triazol-1-yl)quinolin-4-yl)((1S,2R,4S,5R)-5-vinylquinuclidin-2-yl)methanol (**15**):

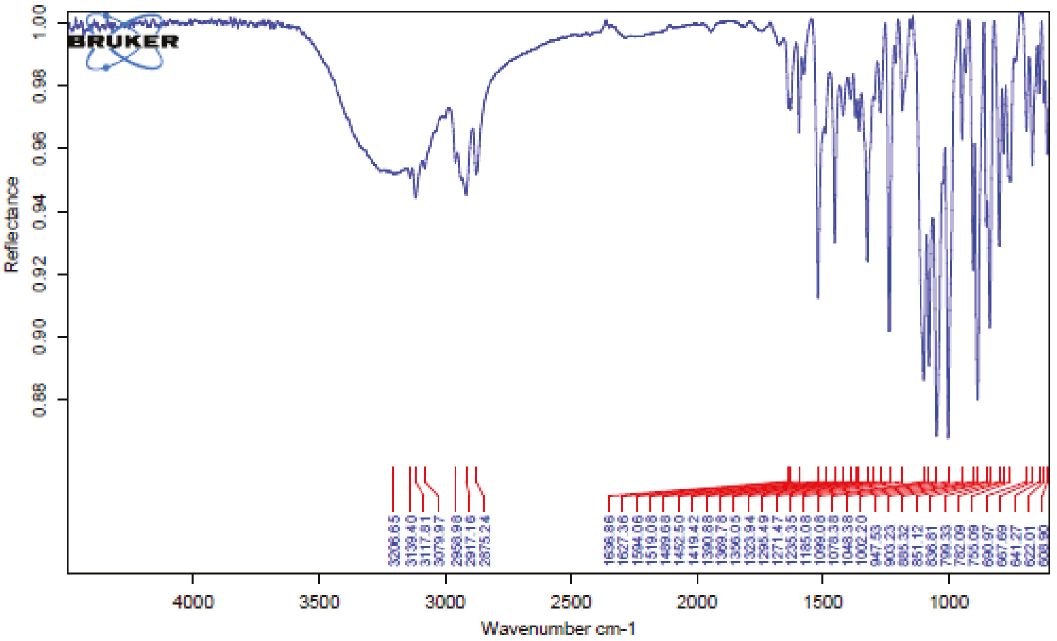

IR spectrogram for (R)-(6-(4-(trimethylsilyl)-1H-1,2,3-triazol-1-yl)quinolin-4-yl)((1S,2S,4S,5R)-5-vinylquinuclidin-2-yl)methanol (**16**):

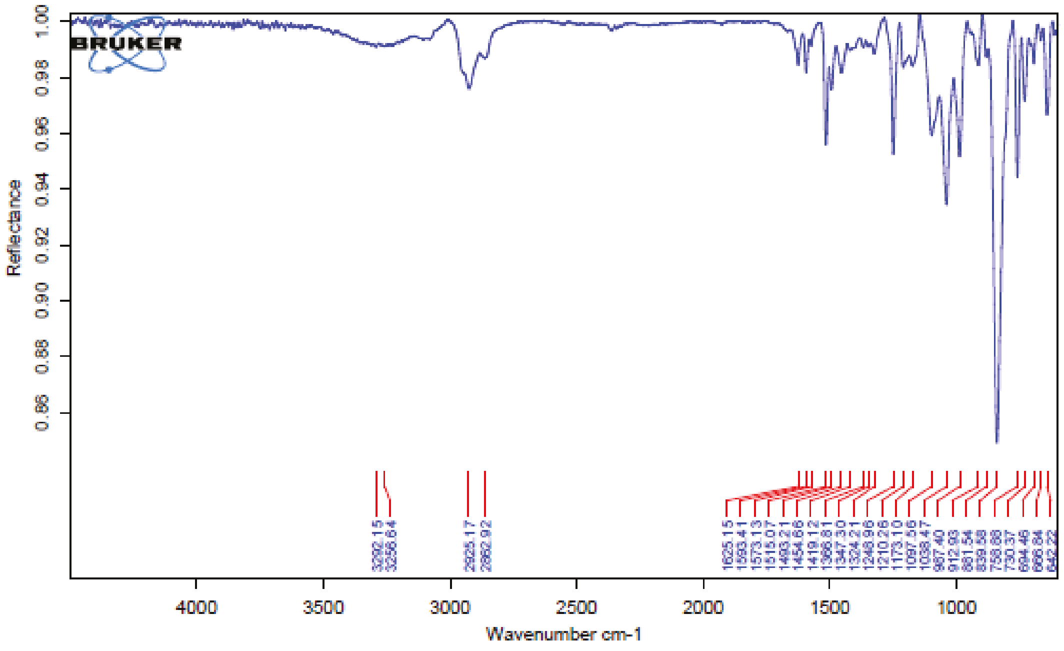

IR spectrogram for (R)-(6-(1H-1,2,3-triazol-1-yl)quinolin-4-yl)((1S,2S,4S,5R)-5-vinylquinuclidin-2-yl)methanol (**17**):

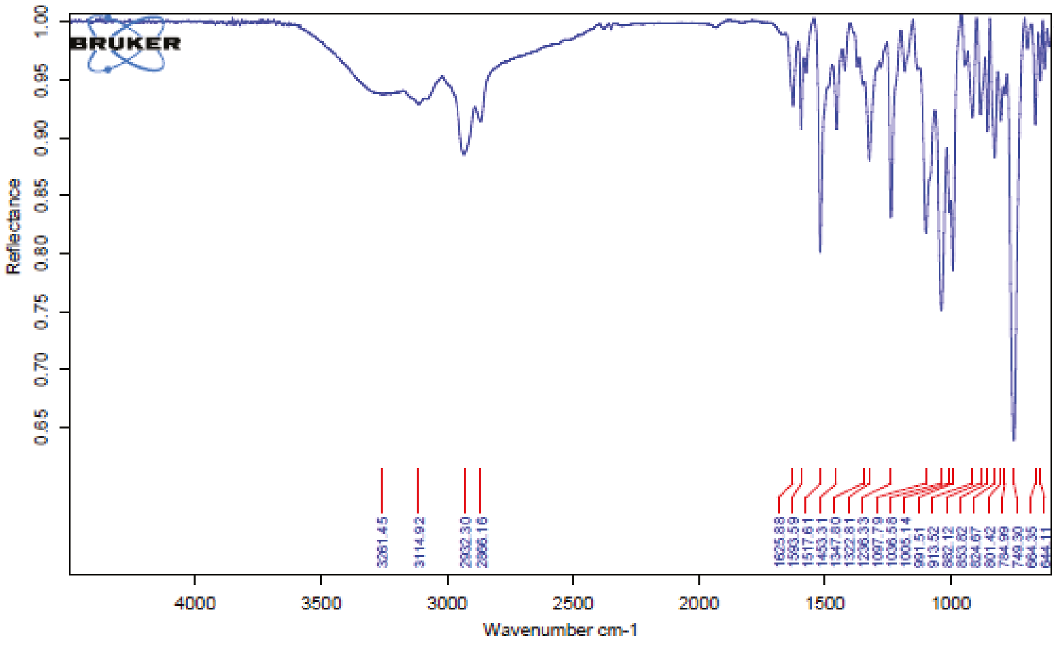

IR spectrogram for (S)-(6-(4-(trimethylsilyl)-1H-1,2,3-triazol-1-yl)quinolin-4-yl)((1S,2R,4S,5R)-5-ethylquinuclidin-2-yl)methanol (**18**):

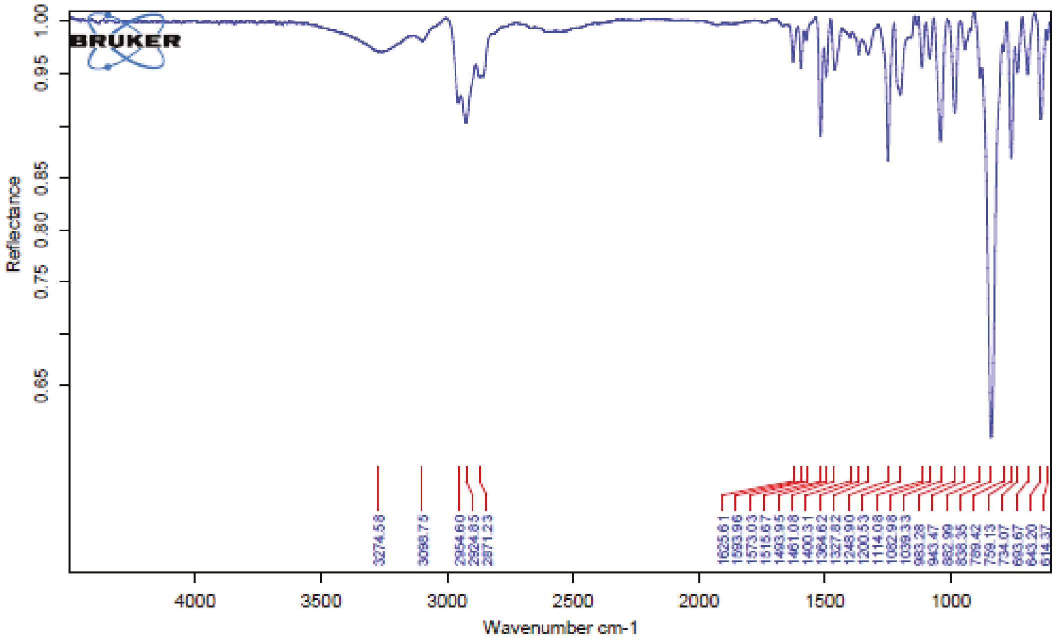

IR spectrogram for (S)-(6-(1H-1,2,3-triazol-1-yl)quinolin-4-yl)((1S,2R,4S,5R)-5-ethylquinuclidin-2-yl)methanol (**19**):

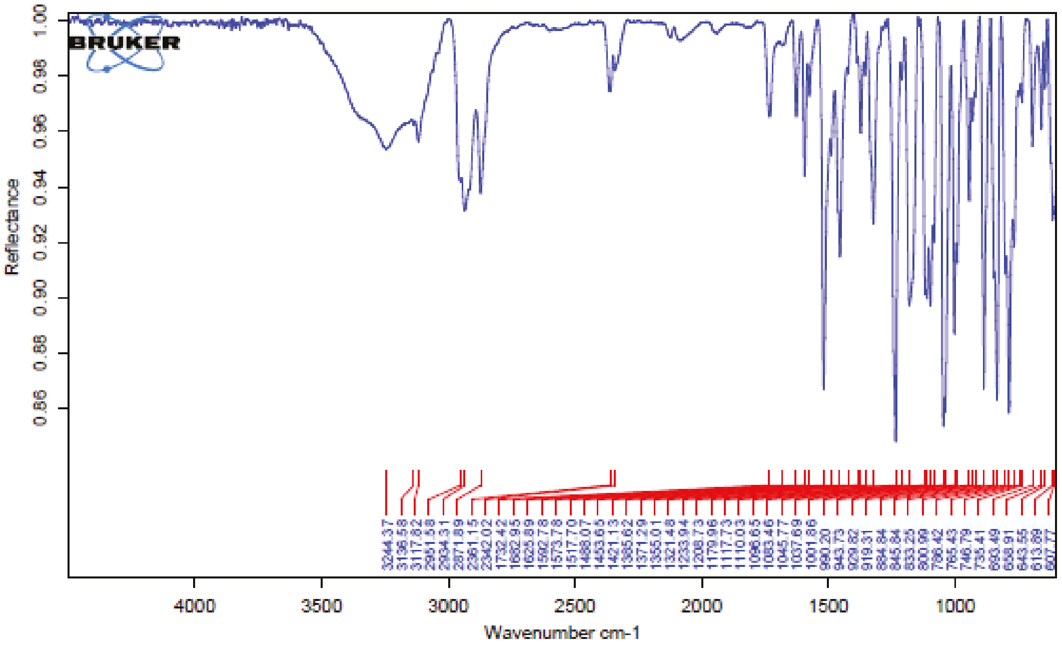

IR spectrogram for (R)-(6-(4-(trimethylsilyl)-1H-1,2,3-triazol-1-yl)quinolin-4-yl)((1S,2S,4S,5R)-5-ethylquinuclidin-2-yl)methanol (**20**):

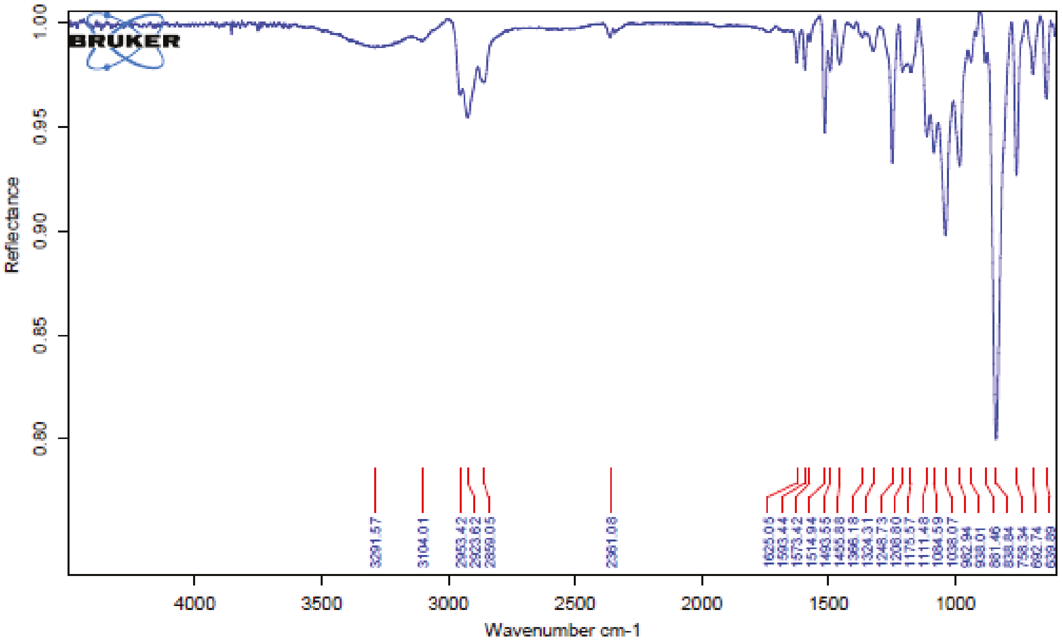

IR spectrogram for (R)-(6-(1H-1,2,3-triazol-1-yl)quinolin-4-yl)((1S,2S,4S,5R)-5-ethylquinuclidin-2-yl)methanol (**21**):

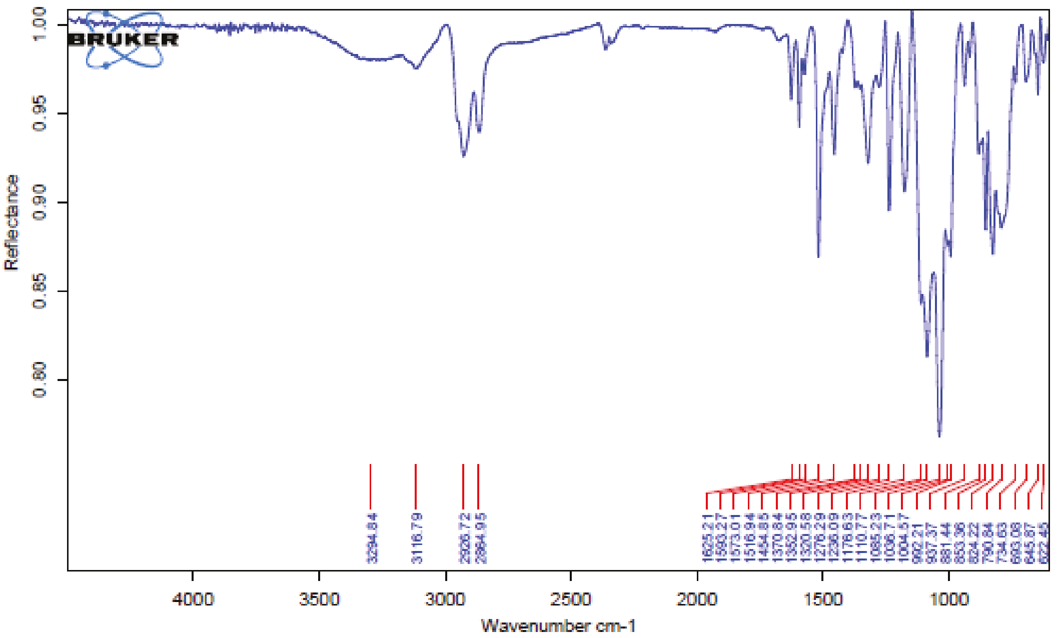

IR spectrogram for (S)-(6-(4-((1S,3R,4S,6R)-6-(hydroxymethyl)quinuclidin-3-yl)-1H-1,2,3-triazol-1-yl)quinolin-4-yl)((1S,2R,4S,5R)-5-vinylquinuclidin-2-yl)methanol (**24**):

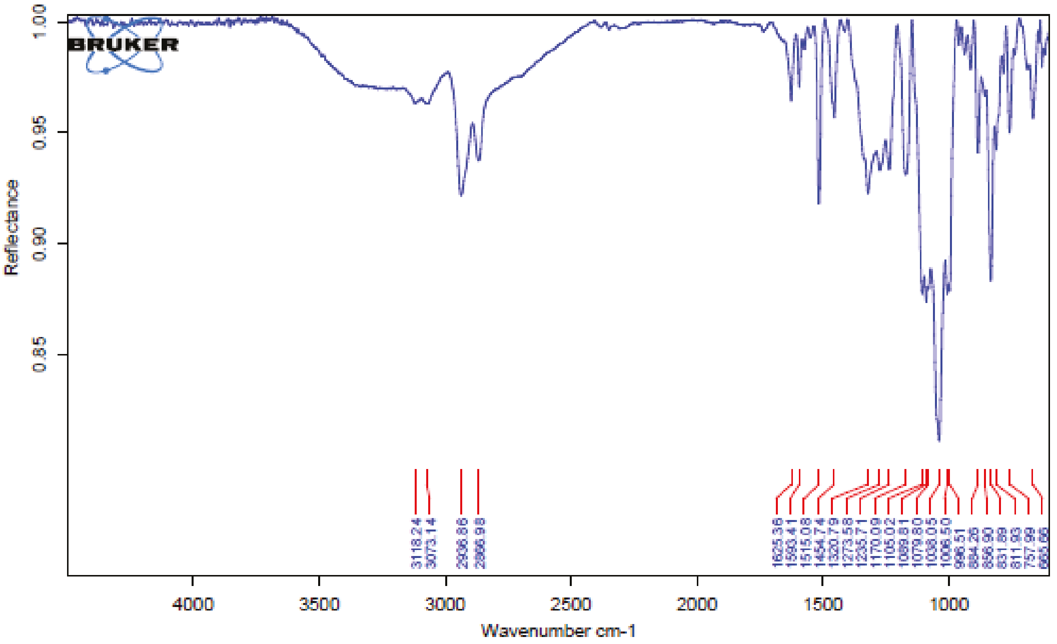

IR spectrogram for (S)-(6-(4-((1S,3R,4S,6S)-6-(hydroxymethyl)quinuclidin-3-yl)-1H-1,2,3-triazol-1-yl)quinolin-4-yl)((1S,2R,4S,5R)-5-vinylquinuclidin-2-yl)methanol (**25**):

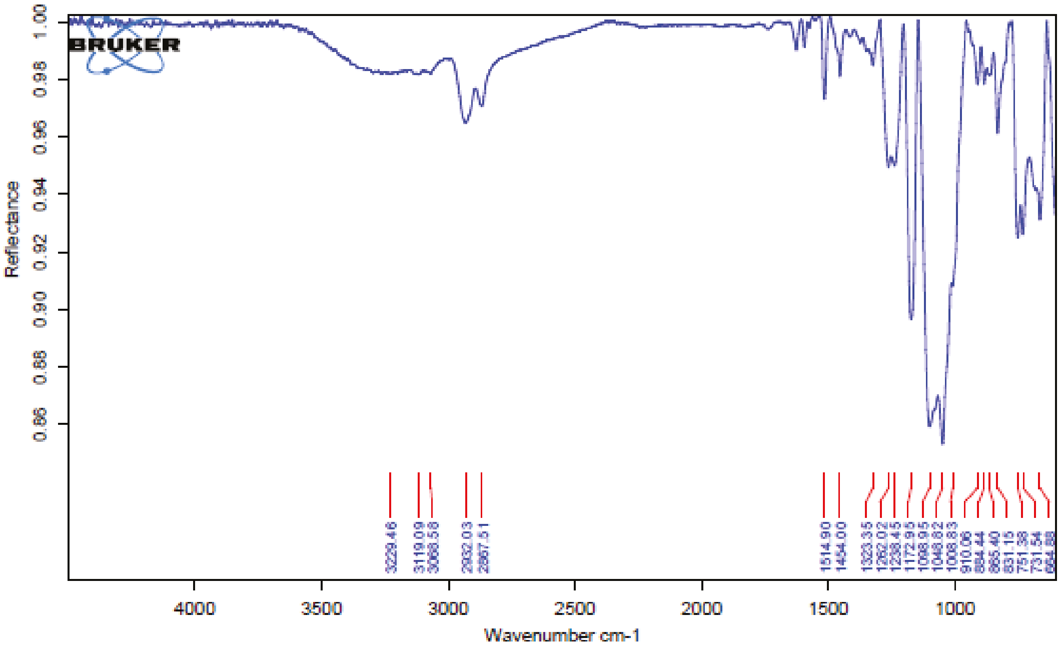

IR spectrogram for (R)-(6-(4-((1S,3R,4S,6R)-6-(hydroxymethyl)quinuclidin-3-yl)-1H-1,2,3-triazol-1-yl)quinolin-4-yl)((1S,2S,4S,5R)-5-vinylquinuclidin-2-yl)methanol (**26**):

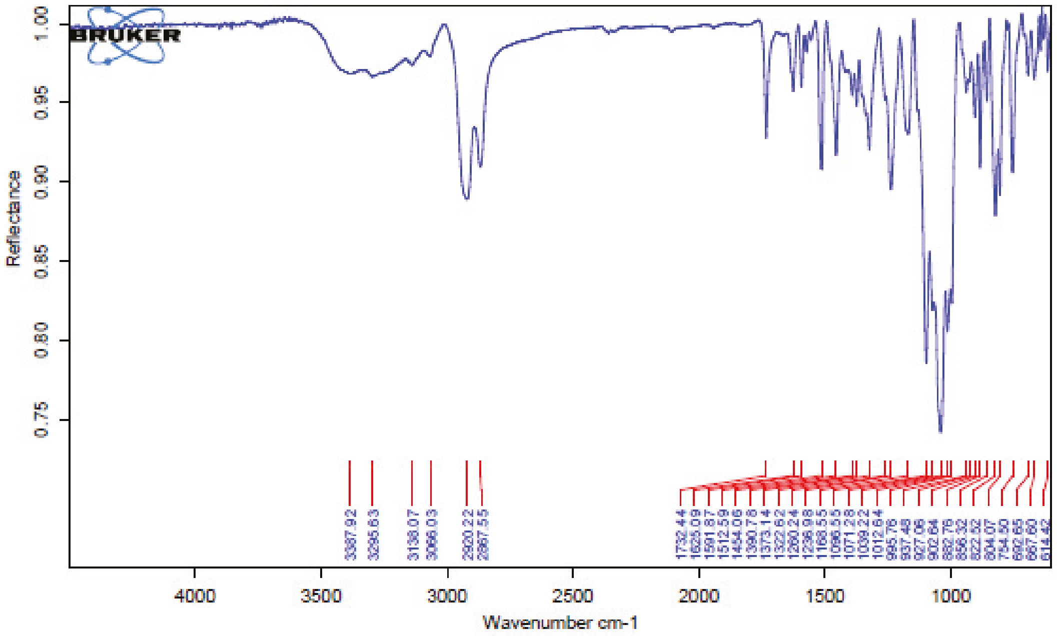

IR spectrogram for (R)-(6-(4-((1S,3R,4S,6S)-6-(hydroxymethyl)quinuclidin-3-yl)-1H-1,2,3-triazol-1-yl)quinolin-4-yl)((1S,2S,4S,5R)-5-vinylquinuclidin-2-yl)methanol (**27**):

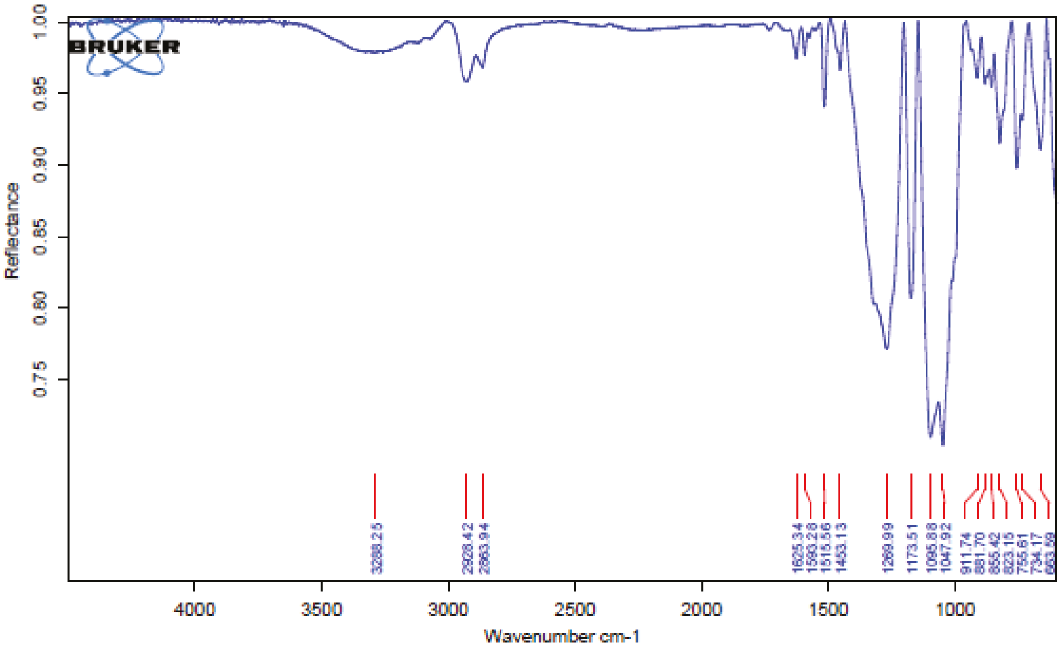

IR spectrogram for (S)-(6-(4-((1S,3R,4S,6R)-6-(hydroxymethyl)quinuclidin-3-yl)-1H-1,2,3-triazol-1-yl)quinolin-4-yl)((1S,2R,4S,5R)-5-ethylquinuclidin-2-yl)methanol (**28**):

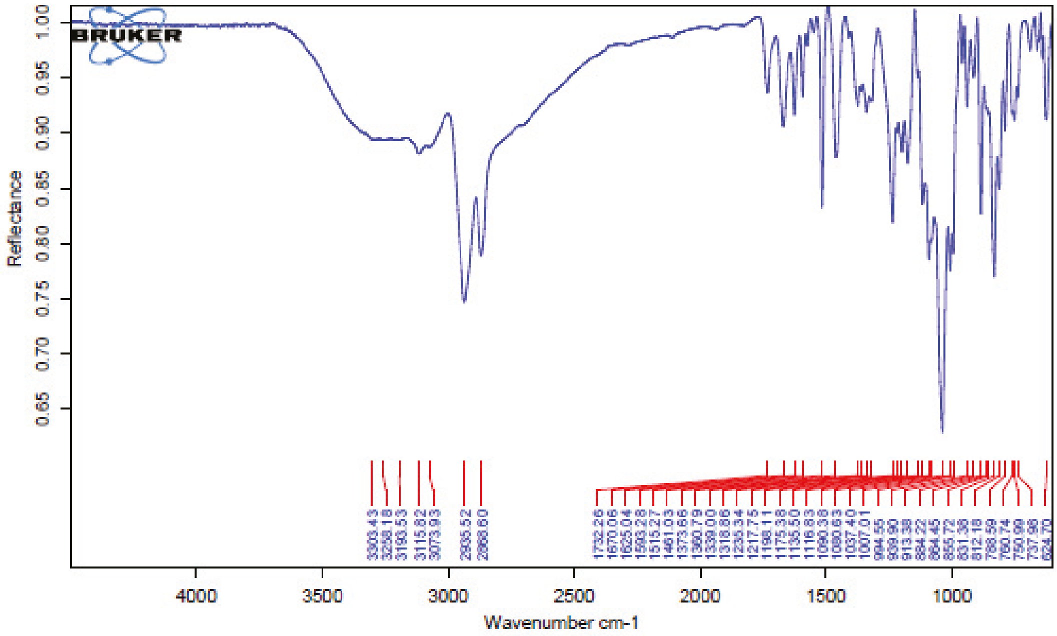

IR spectrogram for (S)-(6-(4-((1S,3R,4S,6S)-6-(hydroxymethyl)quinuclidin-3-yl)-1H-1,2,3-triazol-1-yl)quinolin-4-yl)((1S,2R,4S,5R)-5-ethylquinuclidin-2-yl)methanol (**29**):

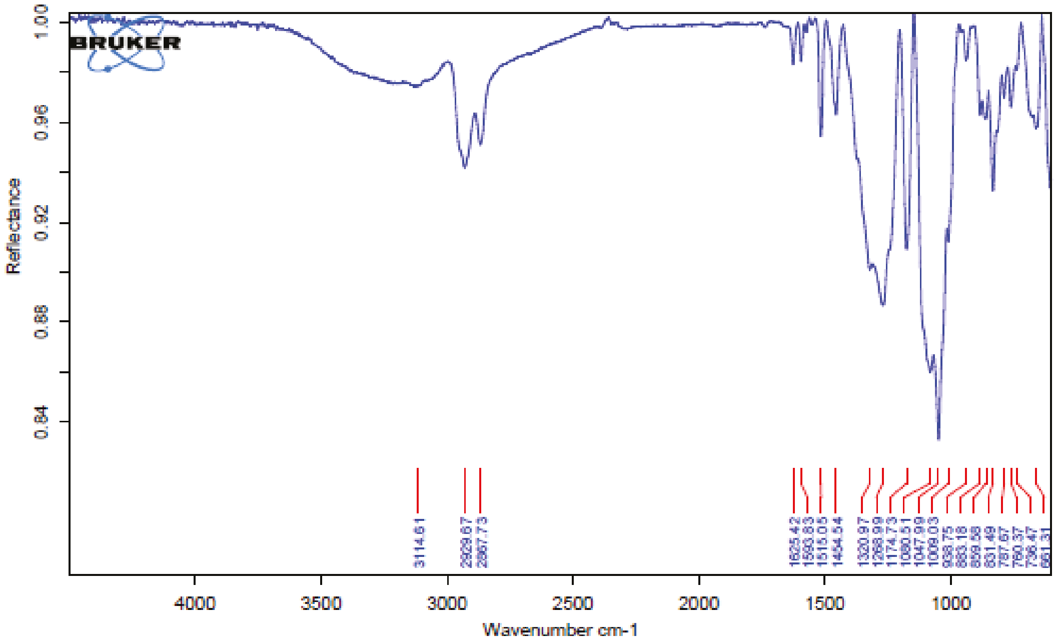

IR spectrogram for (R)-(6-(4-((1S,3R,4S,6R)-6-(hydroxymethyl)quinuclidin-3-yl)-1H-1,2,3-triazol-1-yl)quinolin-4-yl)((1S,2S,4S,5R)-5-ethylquinuclidin-2-yl)methanol (**30**):

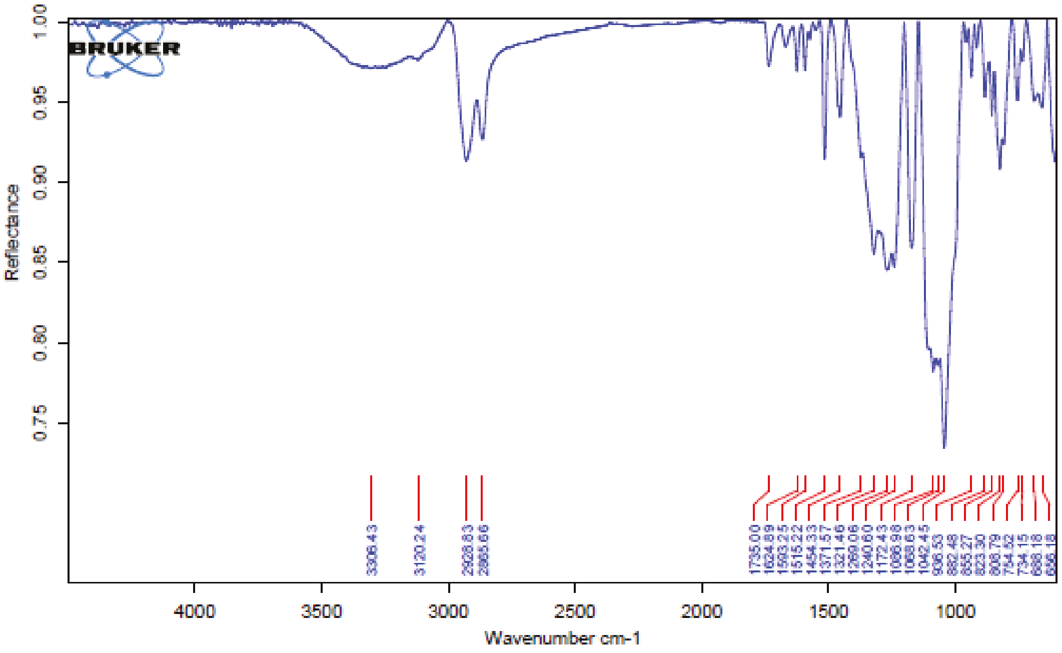

IR spectrogram for (R)-(6-(4-((1S,3R,4S,6S)-6-(hydroxymethyl)quinuclidin-3-yl)-1H-1,2,3-triazol-1-yl)quinolin-4-yl)((1S,2S,4S,5R)-5-ethylquinuclidin-2-yl)methanol (**31**):

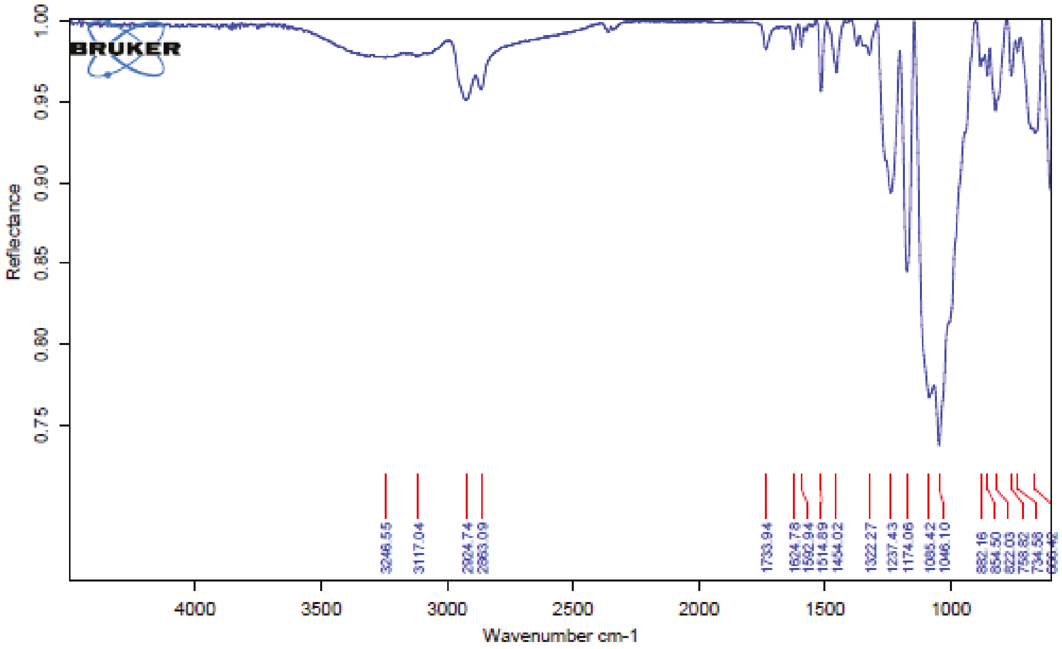

X ray

Table S1. Crystallographic data collection parameter of **10**, **11** and **28**

Compound	**10**	**11**	**28**
Formula	$C_{19}H_{21}N_5O$	$C_{19}H_{23}N_5O_1$, ½ H_2O	$C_{29}H_{38}N_6O_2$, 0.81 HCl, 1.76 H_2O
M_r/(g mol^{-1})	335.41	346.43	564.03
crystal shape	lath	fragment	lath
crystal colour	clear colourless	clear light brown	clear colourless
cryst. dim. /mm^3	0.451 × 0.072 × 0.035	0.220 × 0.118 × 0.022	0.379 × 0.121 × 0.047
crystal system	monoclinic	monoclinic	orthorhombic
space group (no.)	$P2_1$ (4)	$C2$ (5)	$C2_12_12_1$ (19)
a /Å	10.4912(3)	23.1964(4)	8.5435(1)
b /Å	7.6623(2)	6.3113(1)	14.1133(2)
c /Å	10.6905(3)	12.0528(3)	24.5445(3)
α	90°	90°	90°
β	101.591(3)°	98.714°	90°
γ	90°	90°	90°
V /Å^3	841.85(4)	1744.16(6)	2959.50(7)
Z, Z′	2, 1	4, 1	4, 1
$D_{calcd.}$ /(g cm^{-3})	1.323	1.319	1.266
μ /mm^{-1} (λ Å)	0.687 (1.54184)	0.698 (1.54184)	1.338 (1.54184)
Absorption corr.	multi-scan	multi-scan	multi-scan
θ range (compl.)	4.22 – 77.93° (98.9%)	3.71 – 77.74° (98.0%)	4.78 – 77.90° (99.8%)
refl. measured	66650	10314	155939
unique (R_{int})	3554 (0.0843)	3147 (0.0177)	6279 (0.0560)
observed[a]	3446	3096	6221
param. / restr.	230 / 1	240 / 1	387 / 9
R_1 (obs. rflns.)[a]	0.0370	0.0249	0.0408
wR_2 (all rflns.)	0.1012	0.0659	0.1086
GooF on F^2	1.025	1.042	1.058
Abs. Struct. Param.	–0.02(12)	–0.02(7)	0.025(3)
max/min ρ /(eÅ^{-3})	0.173 / –0.227	0.167 / –0.147	0.167 / –0.147
CCDC No.	2080994	2080992	2080993

[a] Observation criterion: $I > 2\sigma(I)$.
